# A comprehensive analysis of avian lymphoid leukosis-like lymphoma transcriptomes including identification of LncRNAs and the expression profiles

**DOI:** 10.1371/journal.pone.0272557

**Published:** 2022-08-08

**Authors:** Kunzhe Dong, Mohammad Heidari, Jody Mays, Shuang Chang, Qingmei Xie, Lei Zhang, Yongxing Ai, Huanmin Zhang

**Affiliations:** 1 USDA, Agricultural Research Service, Avian Disease and Oncology Laboratory, East Lansing, Michigan, United States of America; 2 ORISE Fellow, USDA, Agriculture Research Service, Avian Disease and Oncology Laboratory, East Lansing, Michigan, United States of America; 3 College of Veterinary Medicine, Shandong Agricultural University, Tai’an, Shandong, China; 4 College of Animal Science, South China Agricultural University, Guangzhou, China; 5 Institute of Special Wild Economic Animal and Plant Science, Chinese Academy of Agricultural Sciences, Changchun, Jilin, China; 6 College of Animal Science, Jilin University, Changchun, Jilin, China; University of Kentucky, UNITED STATES

## Abstract

Avian lymphoid leukosis-like (LL-like) lymphoma has been observed in some experimental and commercial lines of chickens that are free of exogenous avian leukosis virus. Reported cases of avian lymphoid leukosis-like lymphoma incidences in the susceptible chickens are relatively low, but the apathogenic subgroup E avian leukosis virus (ALV-E) and the Marek’s disease vaccine, SB-1, significantly escalate the disease incidence in the susceptible chickens. However, the underlying mechanism of tumorigenesis is poorly understood. In this study, we bioinformatically analyzed the deep RNA sequences of 6 lymphoid leukosis-like lymphoma samples, collected from susceptible chickens post both ALV-E and SB-1 inoculation, and identified a total of 1,692 novel long non-coding RNAs (lncRNAs). Thirty-nine of those novel lncRNAs were detected with altered expression in the LL-like tumors. In addition, 13 lncRNAs whose neighboring genes also showed differentially expression and 2 conserved novel lncRNAs, *XLOC_001407* and *XLOC_022595*, may have previously un-appreciated roles in tumor development in human. Furthermore, 14 lncRNAs, especially *XLOC_004542*, exhibited strong potential as competing endogenous RNAs via sponging miRNAs. The analysis also showed that ALV subgroup E viral gene *Gag/Gag-pol* and the MD vaccine SB-1 viral gene *R-LORF1* and *ORF413* were particularly detectable in the LL-like tumor samples. In addition, we discovered 982 novel lncRNAs that were absent in the current annotation of chicken genome and 39 of them were aberrantly expressed in the tumors. This is the first time that lncRNA signature is identified in avian lymphoid leukosis-like lymphoma and suggests the epigenetic factor, lncRNA, is involved with the avian lymphoid leukosis-like lymphoma formation and development in susceptible chickens. Further studies to elucidate the genetic and epigenetic mechanisms underlying the avian lymphoid leukosis-like lymphoma is indeed warranted.

## Introduction

Avian lymphoid leukosis (LL) is the most common naturally occurring neoplasm of chicken caused by avian leucosis viruses (ALV) [1, 2]. LL is a B-cell lymphoma and the cells of the bursa of Fabricius are the principal target cells for neoplastic transformation [3, 4]. LL usually appears in chickens of 4 to 8 months post-infection flowing an orderly progression, and finally results in massive lymphomatosis and death of the host [5, 6]. This kind of tumor causes serious economic losses in the poultry industry worldwide [7, 8].

A similar but different lymphoma in chicken is known as avian lymphoid leukosis-like (LL-like) lymphoma. LL-like lymphoma has been reported in chicken flocks with the characteristics of no exogenous ALV to be found [9–11]. Reported cases of avian lymphoid leukosis-like lymphoma incidences in the susceptible chickens are relatively low, but the apathogenic subgroup E avian leukosis virus (ALV-E) and the Marek’s disease vaccine, SB-1, significantly escalate the LL-like lymphoma incidence in the susceptible chickens [11, 12]. The molecular mechanisms underlying the LL, however, is poorly understood.

Diseases induced by herpesvirus-retrovirus interactions are also frequently found in mammalian, including human. One notable example is that infection with human immunodeficiency virus (HIV) augments replication and pathogenesis of the latent varicella-zoster virus, cytomegalovirus (hCMV), Epstein-Barr virus (EBV), Hepatitis C virus (HCV) and Herpes Simplex virus (HSV) [13–18]. For instance, people living with HIV without treatment are less likely to spontaneously clear HCV infection. In fact, they would have higher HCV viral loads and experience more rapid HCV disease progression than those free of HIV infection [19]. Co-infection is very common because it does not require both viruses to enter a host organism coincidentally since most species do carry latent herpesviruses in cells and are also susceptible to retrovirus infection [20, 21]. It is estimated that there are 130 million HCV infections worldwide, with approximately 4–5 million of them being co-infected with HIV [22]. In the USA, a third of HIV-infected individuals reportedly also suffered from HCV infection [23]. Therefore, it is important to understand the molecular mechanism underlying coinfection-induced diseases. A close examination of the serotype 2 MDV (SB-1) and subgroup E ALV escalated LL-like lymphoma of susceptible chickens at genetic and epigenetic signatures would serve as a valuable biological model and should provide novel insights to advance the understanding on this kind of lymphomas.

Long non-coding RNAs (lncRNAs) are emerging as a serious new player in various diseases, including cancers. LncRNAs regulate protein-coding genes (PCGs) through *in-cis* (or cis-acting on neighboring genes) or *in-trans* (on distal genes) mechanism, such as acting to compete endogenous RNAs to sponge miRNAs and suppress the functions of miRNAs [24]. Several studies have been conducted to understand the function of lncRNAs/miRNAs interaction in tumorigenesis induced by ALV or MDV [25–28]. However, a systematical understanding of the molecular events that occur during tumorigenesis in the LL-like lymphomas resulted from co-infections of ALV and MDV remain grossly unexplored. Here, we re-analyzed the deep RNA sequencing data sets of LL-like lymphomas collected from susceptible chickens that were inoculated with a combination of serotype 2 MDV (SB-1) and a subgroup E ALV (AF227) along with two sets of normal control samples of three bursal tissues and B cell samples taken from three normal chickens of the same genetic line as described by Mays et al [11]. Our aims of this study were 1) to identify key factors of MDV that may be interacted with ALV; 2) to characterize novel chicken lncRNAs and to explore key lncRNA signature that may contribute to the tumorigenesis of LL-like lymphoma via in-cis or competing endogenous RNA mechanism.

## Materials and methods

### Samples used for the RNA-Seq

The avian lymphoid leukosis-like lymphoma samples and the normal bursal and splenic B cell control samples subjected to RNA_Seq are detailly described by Mays et al. [11]. Briefly, the lymphoid leukosis-like bursal lymphoma tissues were collected from six specific pathogen-free (SPF) chickens, which were treated with ALV subgroup isolate AF227 at 7 DOE and MDV SB-1 on the day of hatch, during postmortem examination between 32 and 43 weeks of age. The normal control samples of fresh normal bursal tissues from three 3-week-old non-inoculated SPF chickens and splenic B cells from three non-infected and age-matched SPF chickens of the same genetic line were collected. Total RNA samples were extracted for the RNA_Seq analysis [11].

The original bird trial, from which the lymphoid tissue samples were taken from for total RNA sample extraction in the previous study, was preapproved by and conducted in strict accordance with the Institutional Animal Care and Use Committee’s Guidelines (April, 2005) of the USDA-ARS, Avian Disease and Oncology Laboratory, as detailly described by Mays et al [11]. This study itself did not involve any additional bird experiment.

### Analysis of RNA-seq data

The sets of raw reads RNA_Seq data subjected to analysis in this study are identical to the RNA_Seq datasets publicly available in the NCBI SRA database under assigned project accession number PRJNA543277 (https://www.ncbi.nlm.nih.gov/sra/PRJNA543277). The raw reads of the RNA_Seq data were reprocessed to remove adapter sequence, low quality reads and reads shorter than 50 bp using Trimmomatic (v0.33) software. Clean reads were then aligned to chicken galGal 5 reference genome using Tophat v2.1.0 with parameter of–library type fr-firststrand. Transcript assemblies were generated using Cufflinks v2.2.1 in *de novo* mode and subsequently merged with Cuffmerge to generate a consensus transcriptome across samples. FEELnc program [29] were applied to predict long non-coding intergenic RNAs from the merged transcriptome. The coding potential of each obtained lincRNA was further assessed by the coding potential calculator (CPC) [30]. Transcripts with CPC score larger than zero were eliminated. In this reanalysis, HTseq tool [31] was employed to calculate the raw reads count for each known and novel gene and DESeq2 was used for differential expression analysis. Genes with fold change >2 and FDR <0.01 were considered significant. FPKM value of each gene was calculated, which was used in Principal Component Analysis (PCA) and clustering analysis. Gene function enrichment analysis was conducted using g:Profilier tool [32] and FDR < 0.05 was used as the threshold to determine statistical significance.

For each sample, the unmapped reads were mapped to AF227 and MDV2 SB-1 genome using Bowtie v1.0.0 with default parameters. The genome sequence of AF227 was generated in our lab and deposited in NCBI under the accession number MF817820, and that of MDV2 (SB-1) was downloaded from NCBI (Accession number: HQ840738). CPM (Counts per million mapped reads) were calculated for virus SB-1 and AF227 in each of the 6 tumor samples to identify significantly positively or negatively correlated DEGs with virus abundance (Pearson correlation, *p* <0.05).

### Conservation analysis of chicken lncRNAs

Sequences of human lncRNAs were downloaded from LNCipedia (version 5.2) [33]. NCBI BLASTn was used to identify the sequence homology of all expressed chicken lncRNAs including both known and novel lncRNAs. Human PhastCon conservation data were downloaded from UCSC database and the coordinates of conserved regions between human and chicken genome were converted using UCSC LiftOver tool [34]. PhastCon conservation plots were generated using in custom R scripts.

### Construction of lncRNA-miRNA-mRNA regulatory network

A list of 167 miRNAs that were reported to be differentially expressed in spleens following ALV-J infection [27]. The seed sequence for both -5p and -3p version of these miRNAs were obtained from TargetScan database (https://www.targetscan.org/vert_80/) (S7 Table in S1 File). The 3’UTR sequence for all the DE PCGs and mRNA sequence for all the DE known and novel lncRNAs were retrieved using a custom R script and prediction of the binding sites of the selected miRNAs within these sequences were performed using targetscan_70.pl perl script. Potential competing endogenous lncRNAs were determined by requiring that lncRNAs have at least 10 binding sites for at least one miRNA and opposite change direction in gene expression to that of miRNAs following virus infection. Putative targeted mRNAs of these miRNAs were selected by if the 3’UTR region contains at least one binding site for a miRNA and their regulation upon virus infection is different. Cytoscape was used for visualization of the constructed lncRNA-miRNA-mRNA regulatory network (https://cytoscape.org/).

### Droplet DigitalTM PCR validation of gene expression

To spottily validate the expression of genes determined by RNA-Seq, 8 lncRNA/protein-coding gene pairs showing significantly differential expression from each of the treatment groups were selected and re-evaluated on a Droplet Digital™ PCR (QX200™ ddPCR system; Bio-Rad Laboratories, Inc., Hercules, CA, USA). The ddPCR primers for each of the selected genes were designed with Primer3Plus (http://www.bioinformatics.nl/cgi-bin/primer3plus/primer3plus.cgi/), and are listed in S8 Table in S1 File.

Individual RNA samples were reversely transcribed to cDNA samples used in ddPCR validation (the same samples pooled in preparation of the standard cDNA libraries for RNA-Seq) using the iScriptTM RT Supermix Kit (Cat No. 170–8841) and following the manufacturer’s instructions (Rio-Rad). A ddPCR reaction mixture of 25 μL in final volume was initially prepared per gene per biological sample including 2 μL of cDNA, 12.5 μL of EvaGreen Supermix (Cat No. 1864034), 0.5 μL of each of the forward and reverse primers (200 nM; synthesized by Eurofins Genomics, Huntsville, AL), and 9.5 μL of nuclease-free water. Of these, 20 μL were loaded into one of 8 sample channels of a DG8TM cartridge (Cat No. 1864008, Bio-Rad). Each well was then loaded with 70 μL of droplet generating oil (Cat No. 1864006, Bio-Rad). The loaded DG8TM cartridges were placed on a QX200TM droplet generator (Bio-Rad) to generate the digital droplets. Forty μL of the generated droplet emulsion for each sample were transferred to a well in a 96-well PCR plate followed by polymerase chain reaction with EvaGreen on a C1000TM Thermal Cycler (Bio-Rad). The cycling conditions were 95 °C for 5 min, followed by 40 cycles of 95 °C for 15 s, 58 °C for 60 s, and a final extension step of 98 °C for 10 min. The droplets post PCR were read well by well on a QX200TM droplet reader (Bio-Rad). PCR-positive and PCR-negative droplets in each of the wells were counted and analyzed with the QuantaSoft software (version 1.7, Bio-Rad).

## Results

### Statistics of RNA sequence analyses

The deep RNA sequences (NCBI SRA database Project Accession Number: PRJNA543277) of both the avian lymphoid leukosis-like lymphomas and normal controls were described and published for the transcriptomic variation and differential expression of coding genes by Mays et al [11]. To assess the lncRNAs, the same datasets have been subjected to a reanalysis. The statistics of the RNA sequences from this reanalysis is as follows. A total of about 661 million raw reads pairs were generated from the 12 libraries, which resulted in 427 million clean reads pairs after quality filter and were used in subsequent analyses of this study (S1 Table in S1 File). The clean reads were re-mapped to chicken reference genome galGal5 (S1 Fig in S1 File), which led to mapping rates ranging from 83.8% to 89.0% among individual samples (S1 Table in S1 File). These reads mapped to chicken genome were used for de novo reconstruction of chicken transcriptomes, identification of un-annotated novel lncRNAs, and differential and functional analysis as illustrated in S1 Fig in S1 File. The remaining un-mapped reads were then mapped against AF227 and SB-1 virus genome respectively to examine viral gene expression in the LL-like lymphoma (S1 Fig in S1 File).

### Viral gene expression

**Fig 1:**
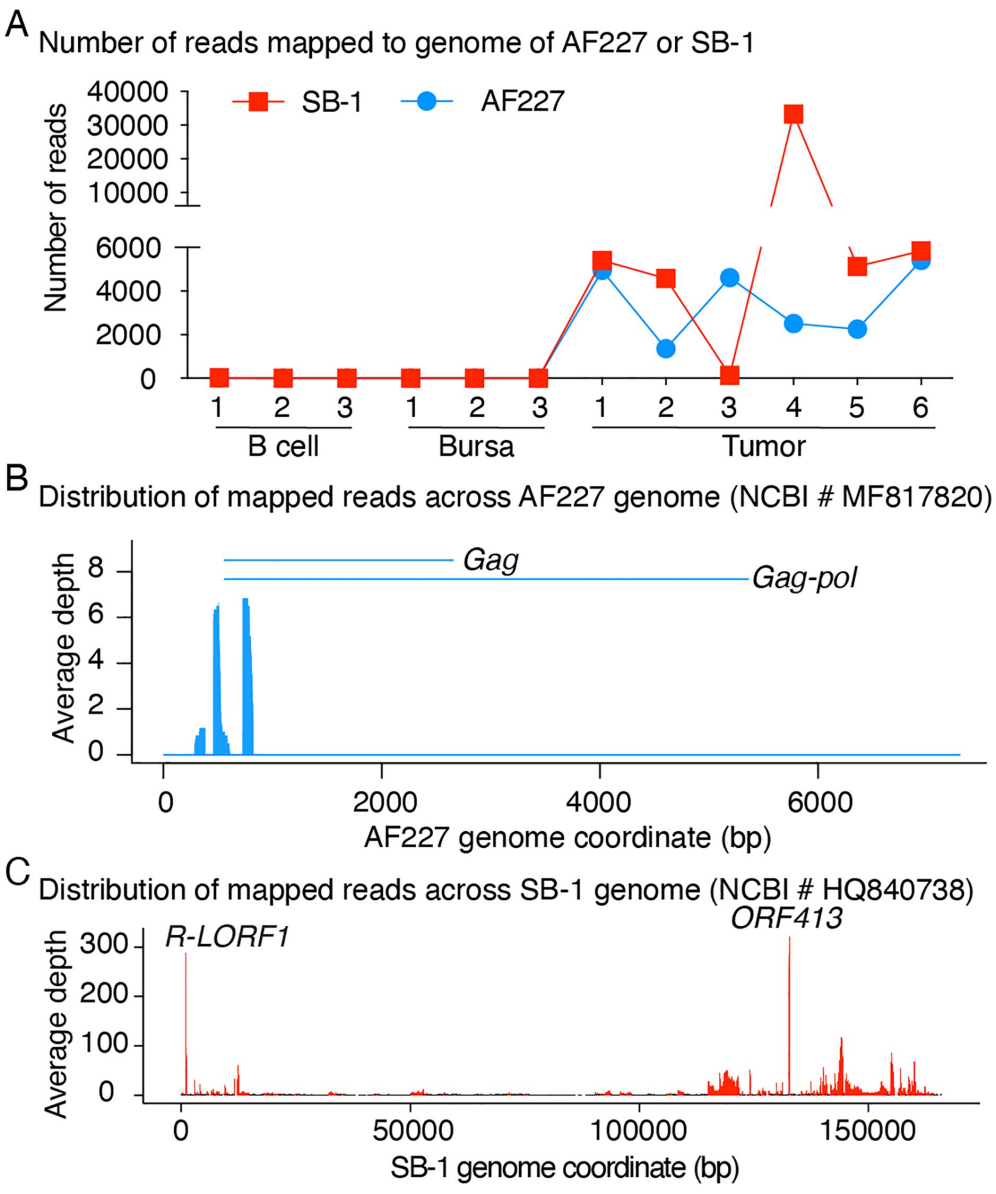
RNA-seq reads mapped to genomes of AF227 and SB-1 viruses. **(A)** Clean RNA-seq reads that failed mapping to the chicken reference genome were further mapped to the genomes of AF227 (NCBI # MF817820) and/or SB-1 (NCBI # HQ840738) viruses. The total number of mapped reads were counted for each virus in each sample. **(B)** Distribution of mapped reads of the LL-like tumor samples across the genome of AF227 and **(C)** SB-1 virus. The Y axis indicates the average depth of each location of the six tumor samples.

A total of 21,116 and 54,408 reads of the six tumor samples were mapped to AF227 and SB-1 genome, respectively, while no or negligible number of reads were mapped to control bursal samples nor splenic B cell samples as expected (Fig 1A and S2 Table in S1 File).

The average numbers of reads per-base across the viral genomes were plotted to examine the viral gene activity in detail. The results showed that nearly all reads mapped to AF227 sequence clustered within a small region of the genome, which overlapped with *Gag* and *Gag-pol* gene (Fig 1B). For SB-1, the mapped reads covered the whole genome. Two obvious peaks, however, were identified within regions of the *R-LORF1* and *ORF413* gene locus (Fig 1C).

### Cluster analysis of samples

**Fig 2:**
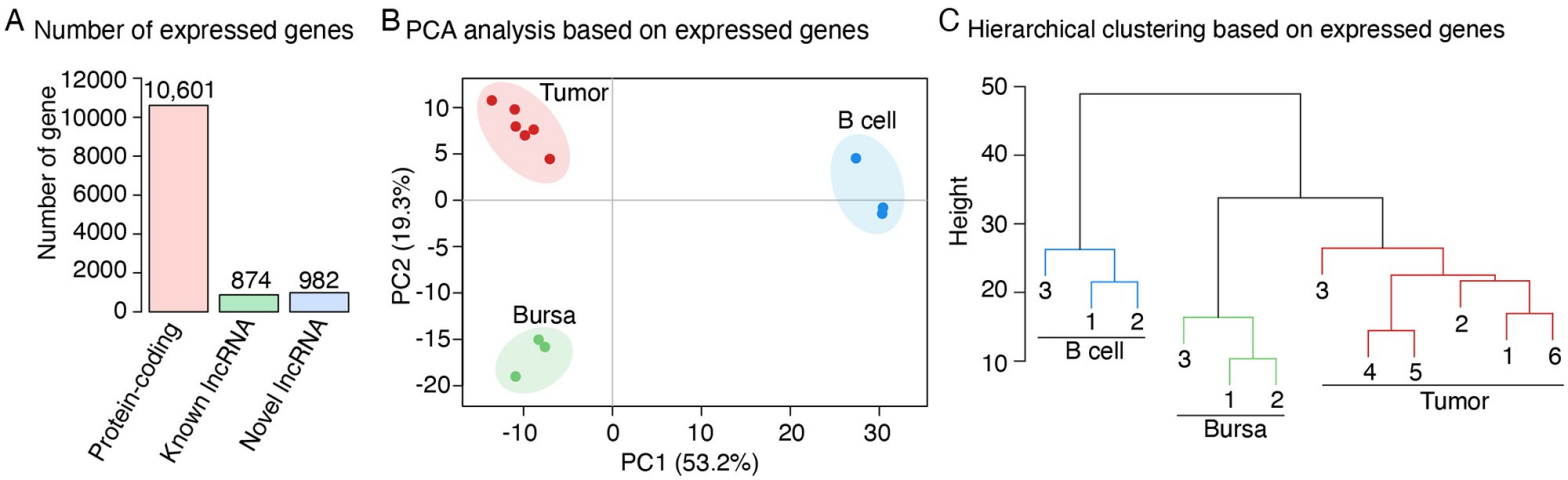
Cluster analysis of the samples. **(A)** A total of 12,457 genes including 10,601 protein-coding genes, 874 known lncRNAs and 982 novel lncRNAs with raw reads counts >10 in at least 4 samples from at least one group were used for clustering and differential expression analysis. **(B)** Principal component analysis (PCA) of all samples based on the 12,457 expressed genes. Samples from the same group are indicated by a same color. **(C)** Hierarchical clustering results of all samples based on the 12,457 expressed genes. Spearman’s correlation was used as distance metric with the average linkage algorithm.

To comprehensively explore the transcriptome in our samples, we first applied a stringent filtering pipeline to identify lncRNAs that were not reported in current annotation of the chicken genome (S1 Fig in S1 File). This analysis led to identification of 1,692 novel lncRNAs. After removing lowly abundant genes with the threshold of raw reads count number >10 in at least four samples in either tumor or control group samples, a total of 10,601, 874 and 982 protein-coding genes, known and novel lncRNAs were kept as expressed genes and used for subsequent analysis, respectively (Fig 2A).

To investigate the relationships in gene expression among samples, a Principal Components Analysis (PCA) was performed for the 12,457 expressed (coding, known and novel lncRNA) genes. The results showed that the samples from each same treatment group clustered together (Fig 2B), with the largest source of variability, PC1, which explained 53.2% of the total transcriptional variation. The LL-like samples were separated from the normal controls, splenic B cell samples and the bursal samples. The PC2 (19.2% of the total variation) also split the tumor samples away from the controls. This result was further confirmed by a following hierarchical clustering analysis (Fig 2C).

#### Differentially expressed protein-coding genes in tumors

**Fig 3:**
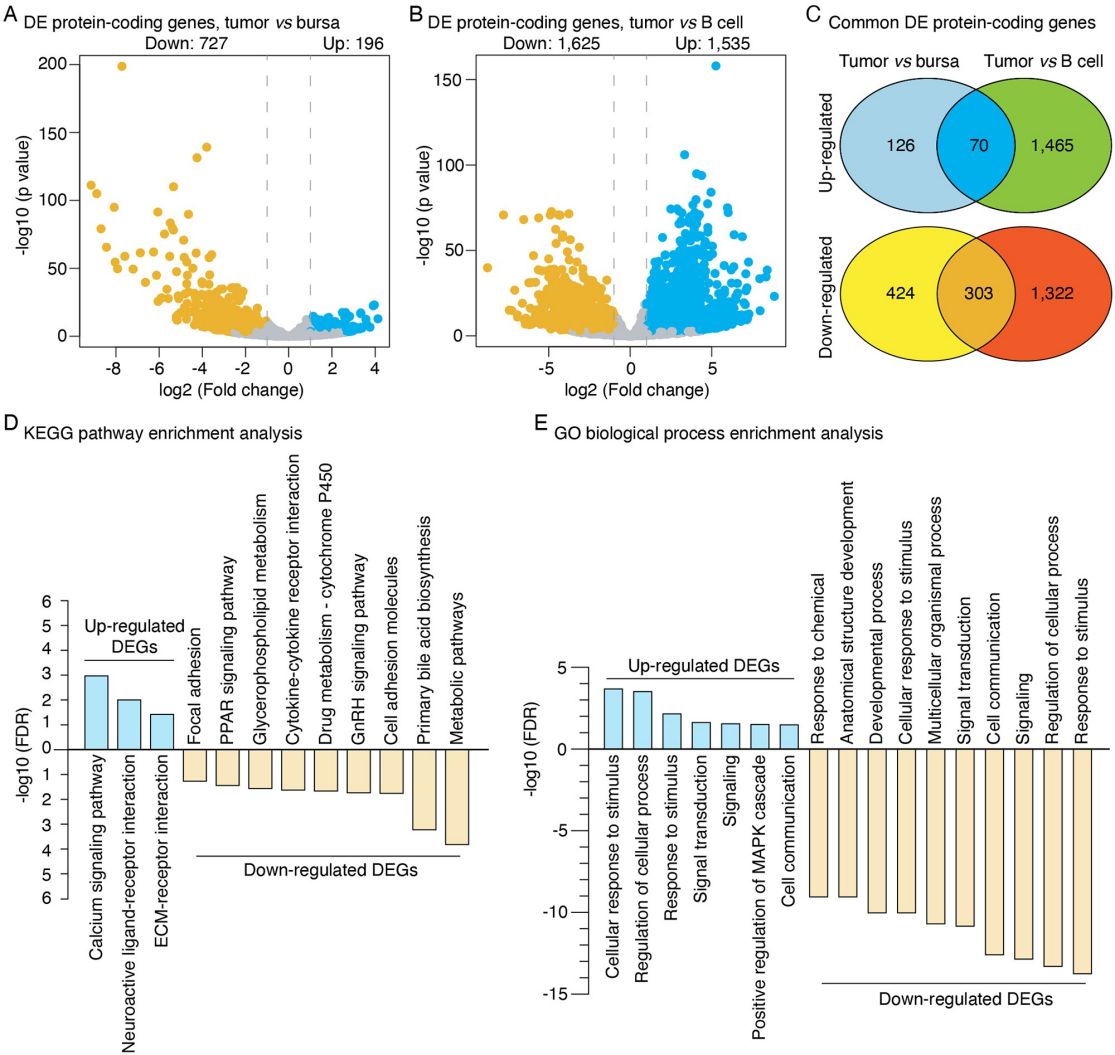
Differentially expressed protein-coding genes and function enrichment analysis. **(A)** Volcano plot showing the numbers of significantly up- and down-regulated protein-coding genes in tumor samples as compared to normal bursal and **(B)** splenic B cell controls. **(C)** Venn diagram showing the number of common significantly up- and down-regulated protein-coding genes identified by both comparison pairs of tumor samples *vs* normal bursal controls and tumor samples *vs* splenic B cell controls. **(D)** Significantly enriched KEGG pathways and **(E)** GO terms of biological process for significant up- and down-regulated PCGs. The top 10 most significantly enriched GO terms of biological processes for down-regulated protein-coding genes were shown.

Of the expressed protein-coding genes, a total of 923 (727 down-regulated and 196 up-regulated) and 3,160 (1,625 down-regulated and 1,535 up-regulated) PCGs were identified from this analysis that were differentially expressed between the LL-like tumor samples and the normal controls, bursal and splenic B cells, respectively (Fig 3A and 3B, and S3 Table in S1 File). Among those, 70 significantly up-regulated and 303 down-regulated genes were identified in both LL-like lymphoma and normal control comparisons (Fig 3C). Thus, those differentially expressed genes (DEGs), perhaps, might be considered to be reliable and relevant DEGs potentially involved with LL-like tumorigenesis.

To gain insights into the biological events that these DEGs were involved with the tumorigenesis, gene function enrichment analysis for the up- and down-regulated DEGs were separately conducted. The up-regulated DEGs were significantly enriched in three KEGG pathways including pathways of “Calcium signaling pathway”, “neuroactive ligand-receptor interaction” and “ECM-receptor interaction”, while the down-regulated DEGs were involved in pathways such as “cell adhesion molecules” and “cytokine-cytokine receptor interaction” (Fig 3D). Interestingly, both up- and down-regulated DEGs were significantly over-presented in GO terms related to immune response, such as “response to stimulus”, “signal transduction” and “cell communication” (Fig 3E and S4 Table in S1 File).

### Differentially expressed known and novel lncRNAs identified in the LL-like lymphomas

Compared to protein-coding genes and currently annotated lncRNAs, a total of 982 novel lncRNAs exhibited fewer exon numbers (S2 Fig in S1 File), shorter transcript length (S2 Fig in S1 File) and lower expression level (S2 Fig in S1 File). Supporting the notion that one of the major functions of lncRNAs is to regulate expression of their neighboring protein-coding genes *in-cis* [35, 36], we observed that these expressed lncRNAs tends to be co-expressed with their nearest protein-coding partners. A sum of 427 and 25 lncRNA/protein-coding gene pairs was detected with correlation of expression > 0.7 and < -0.7 respectively (S3 Fig in S1 File). To gain insights into the potential biological function of these lncRNAs, gene function enrichment analysis was performed for the protein-coding partners. The results indicated that these genes were involved in a broad spectrum of biological process, which included tumorigenesis-related functional categories, such as immune response, cell death and immune cell differentiation (S3 Fig in S1 File). Furthermore, homologous analysis with human lncRNAs suggested that a total of 221 non-coding transcripts derived from 97 non-coding genes were found having human orthologues of lncRNAs (S3 Fig and S5 Table in S1 File). Among them, 60.63% of those lncRNAs have been annotated in chicken genome and 39.37% were identified for the first time in this study (S3 Fig in S1 File).

**Fig 4:**
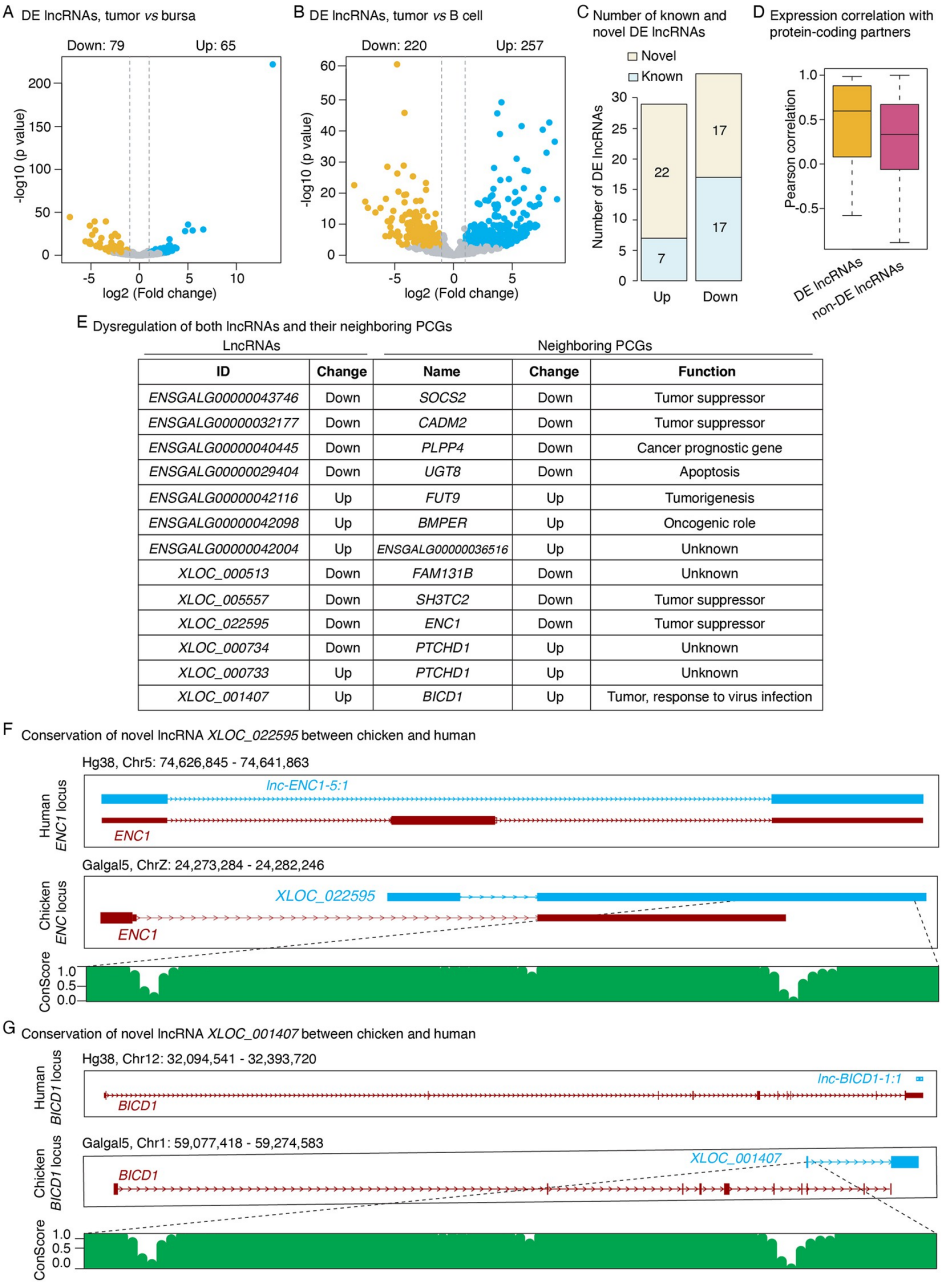
Analysis of differentially expressed lncRNAs. **(A)** Volcano plots showing the numbers of significantly up- and down-regulated lncRNAs in the LL-like tumor samples as compared to normal bursal and **(B)** splenic B cell controls. **(C)** Number of significantly up- and down-regulated commonly known/novel lncRNAs identified by both comparisons between the LL-like tumor samples and normal bursal controls, and LL-like tumor samples and splenic B cell controls. **(D)** Correlation analysis between the expression of significantly differentially expressed (DE) or non-DE lncRNAs and their protein-coding gene partners. **(E)** Significantly dysregulated lncRNA with their protein-coding gene partners exhibited significantly differential expression in the LL-like tumors as compared to the normal controls as well. **(F)** Synteny and sequence conservation of the novel lncRNA *XLOC_0022595* and **(G)** *XLOC_001407*. The PhastCon plot is relative to the loci of human genome.

Differential analysis revealed that the expression of 144 (79 down-regulated and 65 up-regulated) and 477 (220 down-regulated and 257 up-regulated) lncRNAs was altered in the LL-like tumor samples in contrast to the normal controls of bursa and splenic B cell samples, respectively (Fig 4A and 4B and S6 Table in S1 File). There were 29 and 34 lncRNAs that were consistently up-regulated and down-regulated in the LL-like tumor samples as compared to both the bursa and B cell normal controls, respectively (Fig 4C). Among them, 22 up-regulated and 17 down-regulated DE lncRNAs were novel lncRNAs that are absent in the current annotation of chicken genome (Fig 4C).

Analysis of expressional correlation revealed that differentially expressed lncRNAs exhibited higher correlation (Pearson correlation = 0.9) in gene expression with their closest protein-coding partners than those of non-differentially expressed lncRNAs (Pearson correlation = 0.3) (Fig 4D). Interestingly, the nearest protein-coding genes of 13 differentially expressed lncRNAs, which include 6 novel lncRNAs, also showed significant oscillation of expression in tumor samples as compared to and control samples (Fig 4E). Of note, most of these protein-coding genes are previously implicated in tumorigenesis, such as tumor suppression and apoptosis, etc. (Fig 4E).

Of the differentially expressed lncRNA/PCG pairs, two novel differential lncRNAs, *XLOC_022595* and *XLOC_001407*, were found to be conserved in human (Fig 4F and 4G). *XLOC_022595* resides downstream of *ENC1* gene and shows high sequence conservation on part of the first exon (Fig 4F). Similarly, *XLOC_001407* is located nearly downstream of *BICD1* gene on the chicken genome, and the first exon sequence is highly conserved among species (Fig 4G). Their human orthologues, *lnc-ENC1-5*:*1* for *XLOC_022595* and *lnc-FGD4-3*:*1* for *XLOC_001407*, are embodied in *BICD1* and *ENC1* gene, respectively (Fig 4F and 4G).

### Identification of potential competing endogenous lncRNAs

**Fig 5:**
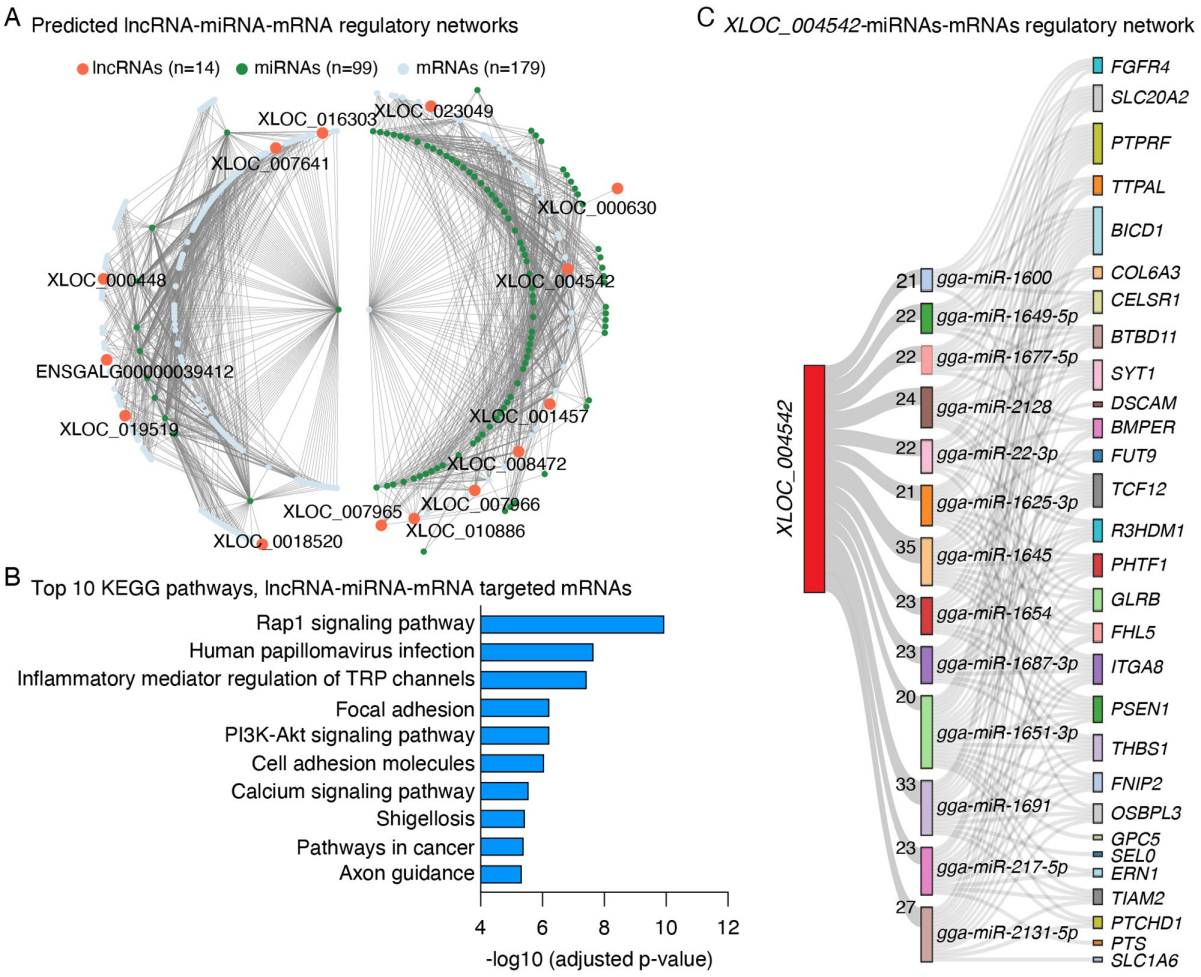
Construction of lncRNA-miRNA-mRNA regulatory network. **(A)** Predicted lncRNA-miRNA-mRNA regulatory network. LncRNAs with at least 10 predicted binding sites for at least 1 miRNA associated with ALV invasion were included. **(B)** Top 10 most significantly enriched KEGG pathways for the 178 differentially expressed protein coding genes identified in the lymphoma samples and potentially regulated by lncRNA-miRNA axis. **(C)** Sankey diagram showing the regulatory network mediated by the novel lncRNA *XLOC_004542* that harbors the largest number of predicted miRNA binding sites. The width of lines linking *XLOC_004542* and miRNAs is proportional to the putative number of miRNA binding sites, which is labeled on the lines.

In an effort to screen for DE lncRNAs that may function as competing endogenous RNAs, we predicted the binding potential of these lncRNAs for a list of previously reported miRNAs implicated in ALV-induced tumorigenesis [27]. By integrating DE PCGs that are putatively targeted by these miRNAs, two separate lncRNA-miRNA-mRNA regulatory networks which involve 14 lncRNAs, 99 miRNAs and 179 PCGs were obtained (Fig 5A). These targeted PCGs were significantly enriched for pathways related to tumorigenesis and virus infection such as “Human papillomavirus infection” and “Pathways in cancer” (Fig 5B). Among the predicted competing endogenous lncRNAs, the novel lncRNA *XLOC_00452* is of particular interest as it harbors more than 20 putative binding sites for multiple miRNAs (Fig 5C), which indicates strong potential as a lncRNA sponge.

### DEGs highly correlated with virus expression

**Fig 6:**
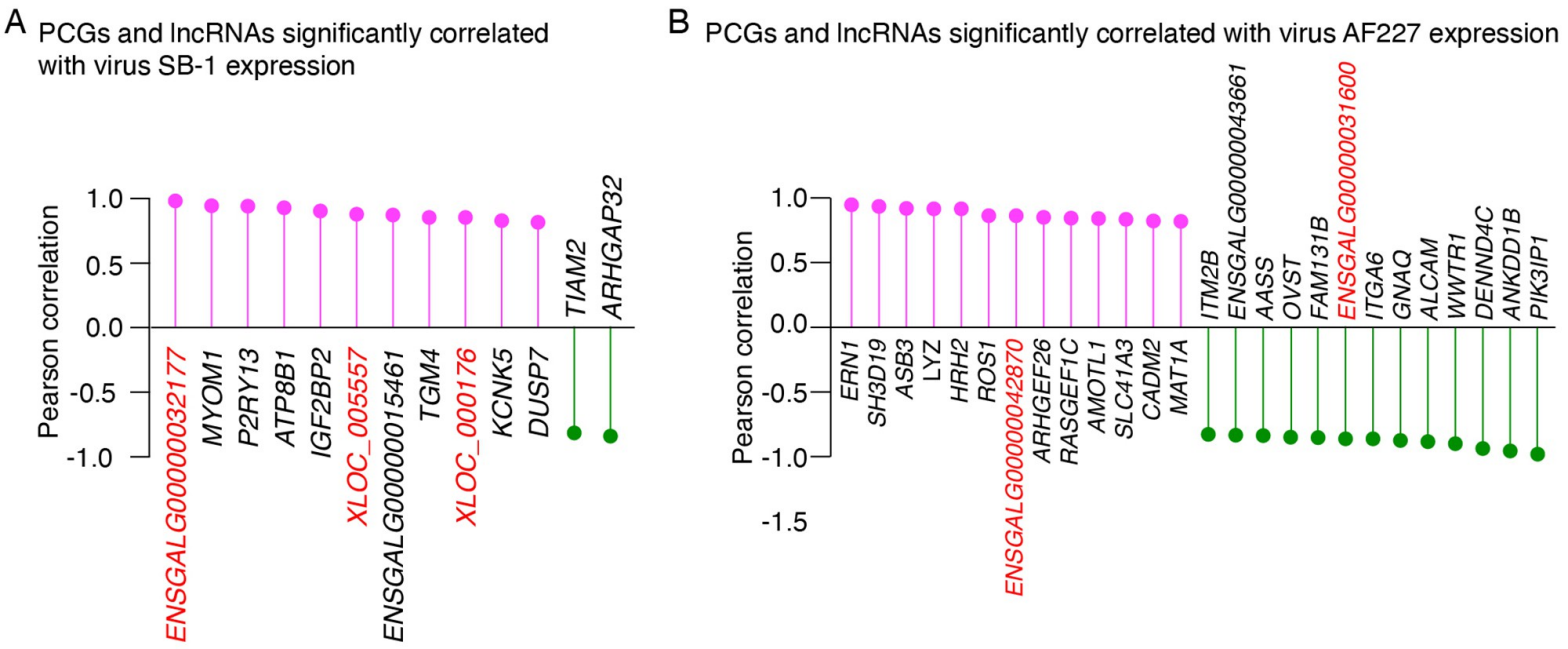
Protein coding genes and lncRNAs that were significantly correlated with virus (A) SB-1 or (B) AF227 expression. The number of RNA-seq reads mapped to each virus genome in each of the tumor samples were normalized to CPM and Pearson correlation between SB-1 and AF227 abundance and all the significantly differentially expressed genes was calculated in 6 tumor samples. *p* <0.05 was used as the threshold to determine statistical significance. The lncRNAs are highlighted in red.

We next sought to identify DE PCGs and lncRNAs with expression correlated virus expression in the 6 tumors. This analysis revealed 11 and 2 DEGs whose expression are significantly positively and negatively correlated with virus SB-1 expression, respectively (Fig 6A). For AF227, 13 DEGs exhibited co-expression pattern, and 13 DEGs showed reverse expression pattern with virus abundance (Fig 6B). Multiple lncRNAs were found among these DEGs correlated with SB-1 or AF227 expression. However, no common DEGs exhibited significant correlation with expression of both viruses.

### Validation of differentially expressed lncRNA/protein-coding gene pairs by ddPCR

**Fig 7:**
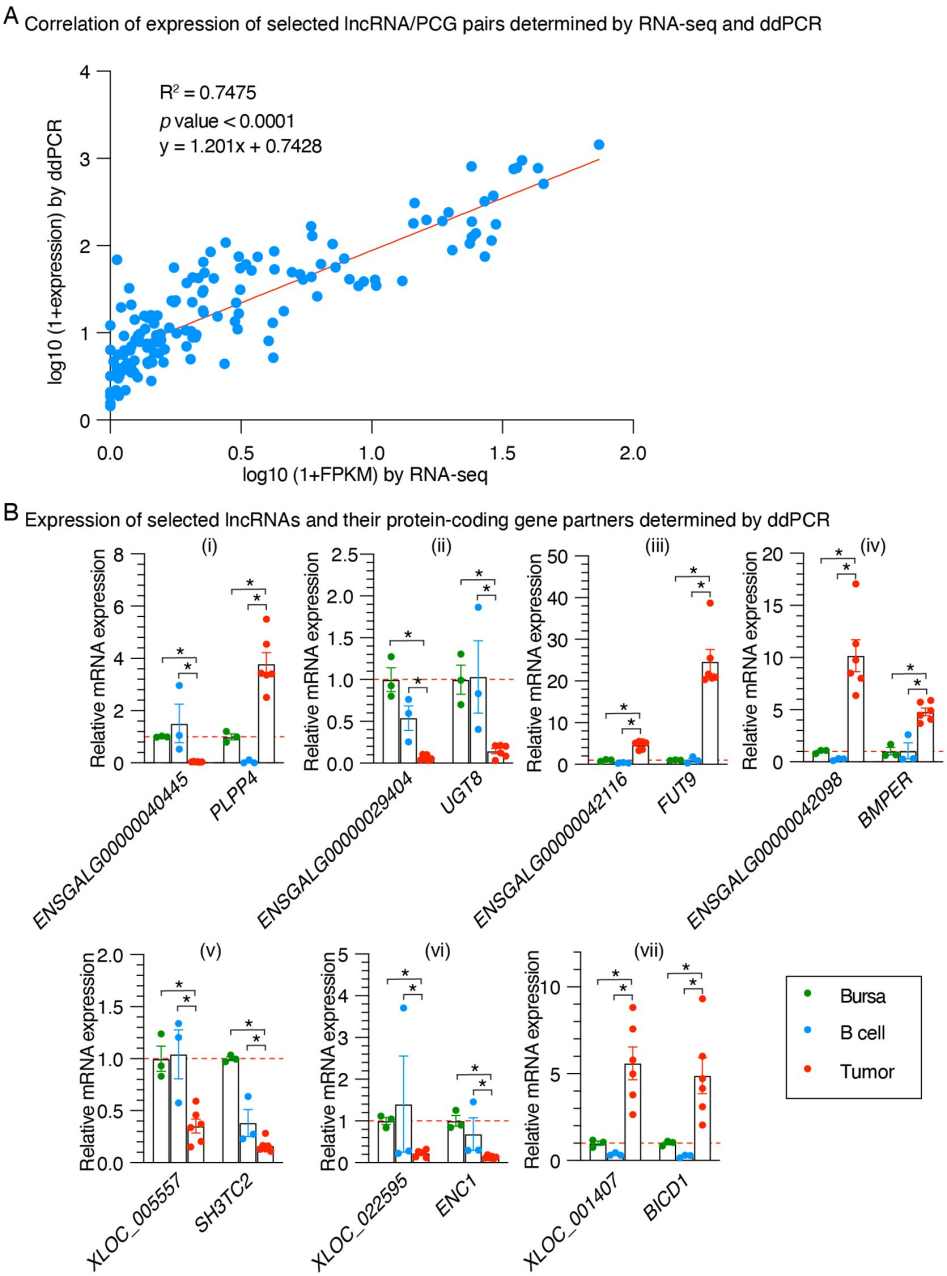
Validation of RNA-seq results by ddPCR. **(A)** Correlation analysis indicating that the expression levels of the 7 selected lncRNA/protein-coding gene pairs identified by RNA-seq and ddPCR were significantly positively correlated with a R^2^ = 0.7475 and *p* value < 0.0001. **(B)** The abundance of the selected lncRNAs and their protein-coding gene partners in individual samples from each of the bursa (n = 3), B cell (n = 3) and the LL-like tumor (n = 3) groups were confirmed by ddPCR (*p < 0.05 in an un-paired *t* test).

To confirm the quality of RNA-seq analysis, we selected 8 significantly expressed lncRNA/PCG pairs, of which the protein-coding gene partners are reportedly to have active function in tumor development, to subject to ddPCR analysis in the individual samples (S8 Table in S1 File). These selected genes included 4 known and 4 novel lncRNAs. One of the pairs (*ENSGALG00000043746/SOCS2*) was excluded from subsequent analysis due to failure of ddPCR analysis for the lncRNA *ENSGALG00000043746* in the tumor samples. The results showed that the gene abundance determined by ddPCR and RNA-seq were highly and positively correlated (R^2^ = 0.7475; *p* value < 0.0001, Fig 7A). Specifically, the expression changes of all the analyzed lncRNAs and their protein-coding partners in tumor samples identified by ddPCR are consistent with RNA-seq results (Fig 7B). These results collectively suggest that our RNA-seq data and differential analysis results should be trustworthy.

## Discussion

In the present study, we used transcriptomic sequence data and generated a broad map of viral and host gene expression profiles that took place in six LL-like lymphoma samples of susceptible chickens. Commonly, exogenous ALV-induced lymphoma is believed to develop following an orderly progression and appears in bursa during 10 to 14 weeks post exposure to the viruses [5]. However, bursa of chickens during that age range usually atrophy naturally [37], which put an obstacle for the study design. The LL-like tumor samples used in this study were collected from chickens aged 32–43 weeks [11]. In the effort to have the most possible appropriate controls, the normal bursa tissues and splenic B cells were collected from 3-week-old and age-matched chickens of the same genetic line, respectively [11]. Since LL is a B cell tumor, the latter age-matched control was expected to help excluding the age effect on gene expressional profiles of this analysis. Use of these controls enabled us to capture both disease causal genes responded to SB-1 and AF227 infection as well as non-causal genes. qRT-PCR and RNA-seq analyses of the samples infected with AFF227 and SB-1 helped in narrowing down the list of causal genes. Cluster analyses suggested that the global gene expression profile of normal bursa control samples was relatively closer to that of tumor samples (Fig 2B and 2C), which is in good agreement with the findings of the previous reports that gene expression patterns of tissues of the same biological origin are similar [11, 38]. To improve the reliability of identification of the tumorigenesis-associated genes, we performed two separate differential expression analyses between the LL-like tumors and each of the normal controls, normal bursa and splenic B cells, and only considered genes that showed significantly differential expression change in both comparisons in subsequent analyses. This stringent strategy may have caused us to miss some authentic genes associated with tumorigenesis but should have improved the reliability of the identified candidates.

Previous studies on human have demonstrated that herpesviruses produce transcription factors capable of transactivating retroviral long-term repeat (LTR) promoters, leading to augmented progression of diseases [15, 16, 39]. Consistent with these reports, emerging evidence has shown that the co-infection of ALV (retrovirus) and non-oncogenic MDV strains (herpesvirus) could increase the incidence of LLs than those birds exposed ALV alone [39–45], suggesting that herpesviruses may serve as important cofactors escalating some retroviral-induced tumor incidence. One of the possible mechanisms underlying this augmentation is that MDV could facilitate the replication of ALV [43, 46] possibly through transactivating the LTR promoter [47]. However, it is still unclear which viral gene or genes of MDV may play a role(s) in the interaction. By mapping the reads that were not derived from chicken genome against virus genomes, we found a large number of the reads that failed to be mapped to chicken genome were derived from SB-1 genome and presented in the LL-like tumor samples, which suggest there may be viral genes somehow involved in the LL-like tumor formation, a finding that is in good agreement with a previous report [48]. Further examination revealed that the depth of coverage at *R-LORF1* and *ORF413* loci of SB-1 was extremely high (Fig 1C), indicating a potentially active stage of these two genes in the LL-like tumors. R-LORF1 of MDV is an Arg-rich protein with unknown function. However, it has high homology with HSV ICP0 [49]. Early transfection studies showed that ICP0 is capable to increase the expression of a wide variety of genes in co-transfected cells, and this regulatory function is likely executed by interacting with other gene-coded proteins since ICP0 does not bind directly to DNA [50]. Recent studies have proposed a number of possible interactions of ICP0 with host proteins, including USP7 [51], cyclin D3 [52], cdk4 [53] and the transcription factor BMAL1 [54]. Interestingly, it also has been reported that HSV ICP0 could strongly enhance expression of a retrovirus [55]. Therefore, we speculated that SB-1 *R-LORF1* gene is a predominant factor enhancing tumorigenesis by interacting with either host genes or ALV. *ORF413* encodes a protein, which has identical sequence with virion coat protein (ORF3) of Gallid alphaherpesvirus 3 (GenBank: AEI00280). Although this analysis of viral gene expression by RNA-seq was through a relatively unbiased and high-throughput manner, in-depth qRT-PCR or ddPCR analyses are to further validate these findings might be warranted in studies.

A total of 373 protein-coding genes were identified showing significant expression change in the LL-like tumor samples in contrast to the normal control samples. Strikingly, the number of down-regulated genes (n = 303, Fig 3C) was observed higher than that of up-regulated genes (n = 70, Fig 3C), which indicates that suppression of host gene expression was likely an important mechanism in subgroup E ALV-induced tumorigenesis. The up-regulated genes were enriched significantly with three KEGG pathways (Fig 3D). One of them was the pathway of extracellular matrix (ECM)-receptor interaction, which has been reportedly shown with a significant increase in invasive non-small cell lung carcinoma [56]. The ECM provides signaling cues that regulate cell behavior and orchestrate functions of cells in tissue formation and homeostasis [57]. We hypothesized that the dysregulation of ECM-related genes may reflect a massive EMC remodeling in lymphoma. Another noticeable enriched-pathway for the up-regulated genes was Calcium signaling pathway (P = 1.92E-02). Calcium-dependent signaling mechanisms are frequently remodeled in cancer cells [58–60]. Besides, many evidence suggest that cytosolic calcium was induced following virus infection and facilitates virus replication [61, 62]. Taken together, the activation of calcium signaling pathway observed here may have contributed to the LL-like tumorigenesis by promoting the duplication of subgroup E ALV and/or SB-1, as well as regulating the tumor cell cycle progression. Evasion of immune system and apoptosis are two prominent characteristics of cancer [63, 64]. Previous studies have shown that upon infection of ALV, the immune cell counts, the level of antibodies and mitogens phytohemagglutinin (PHA) response were decreased in host cells [65, 66]. Also, it has been reported that apoptosis is blocked in MDV-augmented LL-like tumors [67]. Consistent with these reports, our results revealed that the down-regulated genes identified in this study were significantly enriched in functional categories associated with immune system and programmed cell death (Fig 3E). The genes involved in these categories likely represent the genetic basis underlying the immunosuppression and anti-apoptosis observed in MDV-enhanced LL-like lymphoma and contribute to the tumorigenesis.

Emerging evidence showed that lncRNAs play important roles in a wide range of biological processes, including cancer development [68, 69]. Previous studies reported that the expressions of lncRNAs have been altered in various types of human cancers and dysregulated lncRNAs’ function as suppressors and oncogenes [70–74]. Recent reports have increasingly suggested that lncRNAs may be essential actors in infection biology owing to their dysregulation capability during infection processes mostly in response to viral pathogens [75–77]. In this study, we, using a stringent pipeline, have characterized 982 novel lncRNAs that are absent in the current annotation of chicken genome. These newly identified lncRNAs have basic genomic features of shorter transcript size, fewer exon number and lower expression level than that of protein coding genes (S2 Fig in S1 File), which are in good agreement with reported lncRNAs discovered in other species [78–80]. The discovery of these novel lncRNAs also suggested that saturation of lncRNAs in the chicken genome annotation has not been reached and these novel lncRNAs will expand knowledge of the chicken transcriptome. Orthologous analysis indicated that most of the chicken lncRNAs lack detectable homology with human, indicating a poor sequence conservation of lncRNAs between the two species. Differential analysis revealed that 63 lncRNAs, which included 22 up-regulated and 17 down-regulated novel lncRNAs, showed altered expression in tumor samples (Fig 4D), implying the association of lncRNAs with the LL-like tumorigenesis. As one of major role of lncRNAs is to modulate the expression of their neighbor genes via *cis-*action [24], we next sought to identify the key DE lncRNAs with potential roles in expression regulation. We found 13 DE lncRNAs, including 7 known and 6 novel lncRNAs, with the nearest PCGs that also showed significant expression change in the LL-like tumors. Strikingly, most of these PCGs have known functional relevance to cancers. For instance, several down-regulated PCGs in the LL-like tumor samples, including *SOCS2* [81], *CADM2* [82, 83], *SH3TC* [84] and *ENC1* [85], are previously reported as potential tumor suppressors. While, two up-regulated genes, including *FUT9* and *BMPER*, have potential functions in promoting tumorigenesis. *FUT9* is one of the members of α1,3-fucosyltransferases family [86]. Although the function of *FUT9* in tumors not perfectly clear, a previous study showed that suppression of its two gene family orthologs, *FUT1* and *FUT4*, greatly impeded the tumor development [87], suggesting a potential oncogenic role of this gene. *BMPER* is a conserved regulator of hematopoietic and vascular development [88]. It is well known that new blood vessel formation is a fundamental event in the process of tumor growth [89]. Therefore, the elevation of *BMPER* expression would likely facilitate tumorigenesis. More interestingly, we found two differentially expressed novel lncRNAs, *XLOC_001407* and *XLOC_022595*, which displayed both synteny and sequence conservation with two human lncRNAs, *lnc-FGD4* and *lnc-ENC1*, respectively. The closest PCGs of these two lncRNAs are *BICD1* and *ENC1*, respectively. *BICD1* encode a protein Bicaudal-D homolog 1 [90] and has been reported with association of telomere length variation [91], which is implemented in pancreatic cancer cells [92–94]. In addition, a genome-wide association study (GWAS) reported that a SNP located at the second intron of *BICD1* is significantly associated with susceptibility to pancreatic cancer [95]. Another gene, *ENC1*, encodes a BTB/Kelch domain-containing protein and originally cloned in a study searching for transcripts in a p53-induced apoptosis model [96]. The expression of *ENC1* has been shown declined in melanoma cell lines [97] and nervous system tumors [85]. Furthermore, inhibition of *ENC1* could attenuate apoptosis induced by DNA-damage signals [98]. These observations indicated that *ENC1* is a potential tumor suppressor. Collectively, the downregulation of *XLOC_022595* and up-regulation of *XLOC_001407* could probably contribute to the tumorigenesis of LL-like lymphoma in susceptible chickens by *cic*- regulation of their neighbor genes. Currently, the functions of the *lnc-FGD4* and *lnc-ENC1* are poorly understood. However, their sequence conservation with chicken lncRNAs (*XLOC_001407* and *XLOC_022595*) suggest they may play vital roles in cancer development in human.

Another major functional mechanism of lncRNAs is to act as competing endogenous RNAs via sponging miRNAs and inhibiting the function miRNAs [24]. By constructing a lncRNA-miRNA-mRNA regulatory network, our results revealed that 14 lncRNAs mediate 2 networks involving with almost half of the significant DE PCGs (48%, 179/303), which are over-represented in pathways related to virus infections and tumorigenesis (Fig 5). Specifically, a novel lncRNA, namely *XLOC_004542*, exhibited the strongest potential to sponge multiple ALV infection-related miRNAs (Fig 5C). However, it should be noted that the candidate miRNAs used for this analysis were DE miRNAs in spleens following ALV-J infection obtained from a previous study [27], which are different from the lncRNAs and PCGs identified in this study that were differentially expressed in SB-1/AF227 co-infection induced bursal lymphomas. Therefore, functional assays are warranted to validate these findings. In addition, we identified multiple DEGs including several lncRNAs that are highly positively or negatively correlated with expression of virus SB-1 or AF227 (Fig 6). These results provide a candidate list for genes boosting or inhibiting virus activity during tumorigenesis, in spite of the limitation that the sample size for correlation analysis is relatively small.

## Conclusions

In summary, we found that in the subgroup E ALV and SB-1 MDV-induced LL-like lymphomas, several viral genes were activated in both viruses, while a few of immunity- and apoptosis-related protein-coding host genes were suppressed. Furthermore, we identified aberrantly expressed lncRNAs with potential functions in regulating tumorigenesis-related PCGs via *in-cis* or sponging miRNAs in the LL-like tumorigenesis. Two novel lncRNAs, *XLOC_001407* and *XLOC_022595*, may have a role or roles to play in tumorigenesis in human. These findings provide important insights into the molecular mechanisms of tumorigenesis free of detectable exogenous oncogenic viruses. This study may serve as a valuable model for investigating genomic and epigenomic mechanism of cancerous diseases.

## Supporting information

S1 File (DOCX)
