# Supplementary material for: A comprehensive analysis of avian lymphoid leukosis-like lymphoma transcriptomes including identification of LncRNAs and the expression profiles
Source: PLoS One. 2022 Aug 8;17(8):e0272557. doi: 10.1371/journal.pone.0272557 (PMC9359530; doi:10.1371/journal.pone.0272557)
Supplement: S1 File — (DOCX) [file pone.0272557.s001.docx]

**A comprehensive analysis of avian lymphoid leukosis-like lymphoma transcriptomes including identification of LncRNAs and the expression profiles**

Kunzhe Dong^1,2^, Mohammad Heidari^1^, Jody Mays^1^, Shuang Chang^3^, Qingmei Xie^4^, Lei Zhang^5^, Yongxing Ai^6^, Huanmin Zhang^1*^

^1^USDA, Agricultural Research Service, Avian Disease and Oncology Laboratory, East Lansing, MI 48823, U.S.A.

^2^ORISE Fellow, USDA, Agriculture Research Service, Avian Disease and Oncology Laboratory, East Lansing, MI 48823, U.S.A.

^3^College of Veterinary Medicine, Shandong Agricultural University, Tai’an, Shandong 271018, China

^4^College of Animal Science, South China Agricultural University, Guangzhou 510642, China

^5^Institute of Special Wild Economic Animal and Plant Science, Chinese Academy of Agricultural Sciences, Changchun, Jilin 130112, China

^6^College of animal Science, Jilin University, Changchun, Jilin 130062, China

* Correspondence author

E-Mail: Huanmin.Zhang@USDA.GOV

**S1 Table. Sample information and summary of reads and matches**

| **Sample** | **Treatment** | **Tissue** | **Age (week)** | **PF clusters** | **#Clean pairs** | **#Mapped reads** | **Mapping rate (%)** |
| --- | --- | --- | --- | --- | --- | --- | --- |
| Tumor 1 | Tumor | Bursa lymphoma | 16-24 | 49,249,019 | 34,626,934 | 30,582,855 | 87.3 |
| Tumor 2 | Tumor | Bursa lymphoma |  | 37,972,904 | 27,281,873 | 24,104,219 | 88.1 |
| Tumor 3 | Tumor | Bursa lymphoma |  | 93,542,998 | 65,571,649 | 57,483,454 | 86.6 |
| Tumor 4 | Tumor | Bursa lymphoma |  | 43,807,926 | 31,228,679 | 27,616,225 | 88.1 |
| Tumor 5 | Tumor | Bursa lymphoma |  | 38,709,590 | 24,199,444 | 21,466,016 | 88.2 |
| Tumor 6 | Tumor | Bursa lymphoma |  | 90,339,233 | 55,959,683 | 49,283,849 | 87.3 |
| B cell 1 | Control | B cell of spleen | 16-24 | 52,096,499 | 32,413,281 | 27,574,836 | 84.7 |
| B cell 2 | Control | B cell of spleen |  | 40,697,155 | 23,301,720 | 19,683,597 | 83.8 |
| B cell 3 | Control | B cell of spleen |  | 72,903,292 | 45,128,329 | 39,037,549 | 85.9 |
| Bursa 1 | Control | Bursa | 3 | 76,772,884 | 46,573,568 | 41,630,821 | 88.9 |
| Bursa 2 | Control | Bursa |  | 34,194,285 | 21,864,422 | 19,526,392 | 89.0 |
| Bursa 3 | Control | Bursa |  | 30,856,288 | 19,330,017 | 17144211 | 88.3 |

| **Strain** | **Bursa** | | | | **B cell** | | | | **Tumor** | | | | | | |
| --- | --- | --- | --- | --- | --- | --- | --- | --- | --- | --- | --- | --- | --- | --- | --- |
|  | **1** | **2** | **3** | **Total#** | **1** | **2** | **3** | **Total#** | **1** | **2** | **3** | **4** | **5** | **6** | **Total#** |
| AF227 | 0 | 0 | 0 | 0 | 6 | 5 | 0 | 11 | 4,946 | 1,364 | 4,622 | 2,524 | 2,258 | 5,402 | 21,116 |
| SB1 | 0 | 0 | 0 | 0 | 19 | 6 | 1 | 26 | 5,399 | 4,576 | 140 | 33,311 | 5,130 | 5,852 | 54,408 |

**S2 Table. Number of reads mapped to genome of AF227 and SB-1**

**S3 Table. Common significantly up- and down-regulated protein-coding genes in tumor samples as compared to both bursa and B cell controls**

| **ID** | **Name** | **Chr** | **Start** | **End** | **Strand** | **Tumor *vs* Bursa** | | **Tumor *vs* B cell** | |
| --- | --- | --- | --- | --- | --- | --- | --- | --- | --- |
|  |  |  |  |  |  | **Log_2_FC** | **FDR** | **Log_2_FC** | **FDR** |
| ENSGALG00000023933 | G0S2 | 26 | 3140221 | 3141106 | + | 4.13 | 1.46E-13 | 3.42 | 3.88E-10 |
| ENSGALG00000016138 | DSCAM | 1 | 108236302 | 108412621 | - | 3.96 | 8.72E-24 | 6.88 | 9.12E-59 |
| ENSGALG00000014847 | MYOM1 | 2 | 101194874 | 101273319 | + | 3.91 | 3.58E-23 | 5.46 | 7.98E-19 |
| ENSGALG00000042388 | LAMA2 | 3 | 58293669 | 58553018 | - | 3.60 | 4.78E-10 | 7.15 | 3.05E-11 |
| ENSGALG00000030025 | FABP4 | 2 | 121975264 | 121978486 | - | 3.39 | 1.05E-06 | 4.95 | 7.69E-09 |
| ENSGALG00000001416 | ADRA1B | 13 | 7992487 | 8008603 | - | 3.35 | 2.48E-07 | 2.74 | 5.80E-04 |
| ENSGALG00000007868 | BCO2 | 24 | 6110301 | 6130965 | - | 3.34 | 6.90E-18 | 7.26 | 6.68E-44 |
| ENSGALG00000016902 | GPC5 | 1 | 147664506 | 148027057 | - | 3.29 | 4.09E-06 | 3.26 | 2.73E-04 |
| ENSGALG00000006374 | TBX6 | 15 | 8285250 | 8296169 | + | 3.17 | 5.18E-06 | 3.76 | 1.51E-05 |
| ENSGALG00000005995 | SLC13A5 | 19 | 9796159 | 9813490 | + | 3.17 | 2.20E-06 | 5.94 | 1.31E-07 |
| ENSGALG00000009395 | GLRB | 4 | 21510760 | 21553448 | + | 3.11 | 2.52E-06 | 7.20 | 7.85E-11 |
| ENSGALG00000007681 | HTR2B | 9 | 15205069 | 15214169 | - | 3.03 | 6.16E-07 | 6.01 | 1.75E-12 |
| ENSGALG00000041094 | SYT1 | 1 | 39230582 | 39452545 | + | 3.01 | 3.73E-05 | 6.40 | 1.93E-21 |
| ENSGALG00000036516 | ENSGALG00000036516 | 1 | 108608380 | 108614937 | - | 2.97 | 3.79E-08 | 6.50 | 1.10E-09 |
| ENSGALG00000007959 | SLC26A3 | 1 | 14833201 | 14843801 | - | 2.95 | 1.50E-04 | 3.80 | 6.45E-04 |
| ENSGALG00000007314 | DSCAML1 | 24 | 5290222 | 5370245 | - | 2.92 | 6.39E-06 | 5.64 | 1.08E-12 |
| ENSGALG00000002556 | NGF | 26 | 4027896 | 4047968 | - | 2.82 | 3.99E-04 | 4.04 | 4.52E-04 |
| ENSGALG00000020386 | BDKRB1 | 5 | 46461641 | 46462717 | + | 2.65 | 6.40E-07 | 6.38 | 9.57E-17 |
| ENSGALG00000010259 | COL28A1 | 2 | 24735576 | 24796883 | - | 2.60 | 1.51E-04 | 6.27 | 4.48E-12 |
| ENSGALG00000010402 | HPGDS | 4 | 37449944 | 37475275 | - | 2.55 | 3.50E-06 | 5.09 | 1.13E-09 |
| ENSGALG00000036005 | TIAM2 | 3 | 50653163 | 50738444 | + | 2.49 | 3.38E-18 | 3.23 | 7.56E-35 |
| ENSGALG00000012934 | BICD1 | 1 | 59164015 | 59224210 | - | 2.43 | 7.07E-05 | 3.49 | 2.85E-06 |
| ENSGALG00000029811 | BMPER | 2 | 47688331 | 47834840 | - | 2.43 | 1.16E-04 | 3.44 | 5.67E-05 |
| ENSGALG00000000619 | ANGPTL4 | 28 | 838935 | 846067 | - | 2.28 | 9.97E-04 | 4.01 | 4.49E-06 |
| ENSGALG00000013804 | SRD5A3 | 4 | 65542108 | 65548565 | - | 2.21 | 3.39E-05 | 2.58 | 5.88E-06 |
| ENSGALG00000003923 | COL6A3 | 7 | 4808259 | 4861524 | + | 2.18 | 3.88E-05 | 5.63 | 1.31E-16 |
| ENSGALG00000020615 | STK32C | 6 | 34928043 | 34988049 | - | 2.14 | 9.58E-04 | 4.79 | 1.73E-10 |
| ENSGALG00000016358 | PTCHD1 | 1 | 118434845 | 118464479 | - | 1.96 | 3.10E-08 | 3.99 | 1.65E-23 |
| ENSGALG00000015583 | FUT9 | 3 | 73170207 | 73171334 | - | 1.92 | 4.29E-08 | 8.41 | 3.23E-39 |
| ENSGALG00000020251 | FNIP2 | 4 | 22123132 | 22155070 | + | 1.85 | 1.37E-14 | 1.77 | 1.44E-17 |
| ENSGALG00000017031 | ENSGALG00000017031 | 1 | 170492649 | 170509421 | + | 1.83 | 7.54E-09 | 4.63 | 1.44E-38 |
| ENSGALG00000015573 | FHL5 | 3 | 73034632 | 73062348 | - | 1.82 | 7.86E-11 | 7.16 | 8.16E-29 |
| ENSGALG00000016125 | ENSGALG00000016125 | 1 | 108011974 | 108023346 | - | 1.81 | 1.16E-04 | 4.82 | 1.85E-22 |
| ENSGALG00000014011 | LRMP | 1 | 67203135 | 67215800 | + | 1.79 | 5.27E-14 | 1.78 | 1.28E-10 |
| ENSGALG00000009320 | PSEN1 | 5 | 26365084 | 26386235 | - | 1.69 | 3.19E-06 | 1.36 | 1.80E-06 |
| ENSGALG00000024171 | HRH2 | 13 | 10466445 | 10469938 | - | 1.69 | 3.39E-07 | 1.26 | 2.41E-04 |
| ENSGALG00000037366 | GRID1 | 6 | 1638930 | 1943696 | - | 1.65 | 7.42E-04 | 4.95 | 1.50E-14 |
| ENSGALG00000001845 | PHTF1 | 26 | 3719995 | 3729665 | - | 1.64 | 3.83E-10 | 1.15 | 1.16E-05 |
| ENSGALG00000027592 | ERN1 | 14 | 6942340 | 6951189 | - | 1.56 | 1.05E-09 | 1.94 | 1.60E-14 |
| ENSGALG00000009626 | THBS1 | 5 | 29636380 | 29652162 | - | 1.52 | 4.95E-04 | 4.87 | 2.62E-15 |
| ENSGALG00000000558 | SLC1A6 | 28 | 682646 | 704983 | - | 1.52 | 1.26E-06 | 1.93 | 4.52E-04 |
| ENSGALG00000011007 | OSBPL3 | 2 | 31704354 | 31771000 | - | 1.51 | 5.09E-06 | 1.64 | 4.18E-08 |
| ENSGALG00000041854 | ENSGALG00000041854 | 4 | 76488439 | 76523293 | + | 1.46 | 2.39E-04 | 3.20 | 1.33E-15 |
| ENSGALG00000033470 | ADGRA3 | 4 | 74924347 | 74979186 | + | 1.46 | 4.24E-08 | 2.65 | 4.16E-28 |
| ENSGALG00000038467 | SELO | 1 | 20290347 | 20303283 | + | 1.43 | 5.03E-04 | 1.37 | 9.60E-04 |
| ENSGALG00000004304 | TCF12 | 10 | 7176016 | 7515965 | - | 1.34 | 9.74E-06 | 2.15 | 4.49E-48 |
| ENSGALG00000007045 | ENSGALG00000007045 | 6 | 21623563 | 21878905 | - | 1.33 | 6.96E-04 | 3.98 | 1.72E-11 |
| ENSGALG00000012021 | ENSGALG00000012021 | 5 | 55080812 | 55090827 | + | 1.29 | 2.23E-04 | 7.97 | 2.11E-15 |
| ENSGALG00000032440 | QPCT | 3 | 33675543 | 33688162 | + | 1.28 | 5.01E-04 | 3.25 | 2.07E-12 |
| ENSGALG00000027056 | ENSGALG00000027056 | 1 | 59476229 | 59483268 | - | 1.28 | 4.09E-04 | 1.44 | 1.84E-04 |
| ENSGALG00000028543 | FGFR4 | 13 | 10261730 | 10269384 | - | 1.25 | 3.99E-04 | 1.56 | 4.67E-06 |
| ENSGALG00000008747 | ITGA8 | 2 | 20364201 | 20463104 | + | 1.24 | 1.15E-04 | 3.44 | 2.50E-06 |
| ENSGALG00000016636 | ARSH | 1 | 128491915 | 128504486 | + | 1.22 | 8.95E-04 | 4.79 | 8.41E-10 |
| ENSGALG00000012246 | R3HDM1 | 7 | 30736705 | 30778328 | + | 1.21 | 1.70E-04 | 1.30 | 3.38E-05 |
| ENSGALG00000036234 | ENSGALG00000036234 | 11 | 1929764 | 1944429 | - | 1.21 | 3.69E-07 | 1.48 | 4.59E-09 |
| ENSGALG00000038336 | SLC20A2 | 4 | 34431805 | 34488498 | + | 1.17 | 2.31E-04 | 2.23 | 1.05E-12 |
| ENSGALG00000017024 | NEK3 | 1 | 170393901 | 170406389 | - | 1.16 | 1.49E-04 | 3.06 | 3.91E-21 |
| ENSGALG00000016569 | FANCB | 1 | 122344605 | 122359048 | + | 1.15 | 2.86E-05 | 3.68 | 2.83E-37 |
| ENSGALG00000015461 | ENSGALG00000015461 | 1 | 91922427 | 91932287 | + | 1.14 | 1.64E-04 | 1.50 | 9.07E-08 |
| ENSGALG00000006123 | ENSGALG00000006123 | 20 | 9857387 | 9895824 | - | 1.13 | 5.25E-06 | 1.54 | 4.33E-10 |
| ENSGALG00000008176 | ASB3 | 3 | 3040531 | 3066219 | + | 1.11 | 4.99E-04 | 2.76 | 1.29E-19 |
| ENSGALG00000007181 | PGS1 | 18 | 9912347 | 9929719 | - | 1.11 | 9.04E-08 | 1.41 | 3.21E-11 |
| ENSGALG00000012223 | ZRANB3 | 7 | 30677308 | 30704598 | - | 1.08 | 8.70E-05 | 2.56 | 1.11E-15 |
| ENSGALG00000039595 | BTBD11 | 1 | 53427816 | 53497435 | - | 1.06 | 8.54E-05 | 1.91 | 4.10E-04 |
| ENSGALG00000007848 | PTS | 24 | 6106022 | 6108646 | - | 1.05 | 5.97E-05 | 2.70 | 7.99E-20 |
| ENSGALG00000004110 | ENSGALG00000004110 | 20 | 5370082 | 5373738 | + | 1.04 | 6.88E-04 | 1.28 | 1.14E-05 |
| ENSGALG00000004244 | TTPAL | 20 | 5549544 | 5557118 | - | 1.03 | 5.59E-04 | 2.05 | 6.46E-10 |
| ENSGALG00000040298 | TPK1 | 2 | 52848375 | 53196795 | - | 1.02 | 5.11E-05 | 1.11 | 5.54E-05 |
| ENSGALG00000010053 | PTPRF | 8 | 19988821 | 20225336 | + | 1.01 | 3.37E-04 | 5.33 | 8.79E-31 |
| ENSGALG00000043355 | CELSR1 | 1 | 15740825 | 15899595 | - | 1.01 | 2.92E-04 | 5.97 | 1.23E-75 |
| ENSGALG00000010211 | ALDH6A1 | 5 | 37778732 | 37788095 | - | -1.00 | 3.36E-05 | 5.29 | 6.07E+00 |
| ENSGALG00000004618 | TAX1BP3 | 19 | 6641025 | 6644584 | + | -1.01 | 4.79E-11 | 39.32 | 4.05E+01 |
| ENSGALG00000015083 | DENND4C | Z | 33679446 | 33728466 | + | -1.01 | 1.94E-05 | 2.76 | 4.94E+00 |
| ENSGALG00000004974 | PPARG | 12 | 4859854 | 4880521 | + | -1.02 | 1.45E-04 | 4.81 | 5.11E+00 |
| ENSGALG00000009222 | FNDC3B | 9 | 19398182 | 19545817 | - | -1.03 | 1.18E-06 | 2.38 | 2.34E+00 |
| ENSGALG00000015780 | PNRC1 | 3 | 76102961 | 76105220 | - | -1.04 | 4.87E-06 | 112.63 | 1.49E+02 |
| ENSGALG00000029571 | TMEM55A | 2 | 125284297 | 125307712 | - | -1.05 | 3.40E-05 | 3.95 | 6.05E+00 |
| ENSGALG00000015682 | UGCG | Z | 66254769 | 66279863 | - | -1.05 | 2.04E-04 | 7.05 | 8.79E+00 |
| ENSGALG00000042803 | ENSGALG00000042803 | 15 | 555264 | 562933 | + | -1.06 | 1.93E-04 | 49.97 | 3.66E+01 |
| ENSGALG00000004948 | GLA | 4 | 2034276 | 2039395 | + | -1.07 | 1.48E-04 | 2.67 | 3.74E+00 |
| ENSGALG00000001944 | TBC1D2 | Z | 70527243 | 70548597 | - | -1.09 | 8.30E-04 | 0.84 | 8.87E-01 |
| ENSGALG00000011162 | CYP46A1 | 5 | 48244148 | 48259476 | + | -1.09 | 2.52E-04 | 1.91 | 3.53E+00 |
| ENSGALG00000007346 | KIAA1462 | 2 | 14907168 | 14932071 | - | -1.10 | 8.24E-04 | 1.33 | 1.32E+00 |
| ENSGALG00000002187 | HSD17B4 | Z | 71546997 | 71602452 | - | -1.11 | 3.94E-04 | 21.28 | 3.73E+01 |
| ENSGALG00000012659 | CKAP4 | 1 | 53953941 | 53959022 | + | -1.12 | 1.71E-05 | 18.40 | 1.49E+01 |
| ENSGALG00000002606 | CLSTN1 | 21 | 3489330 | 3523996 | - | -1.13 | 2.36E-08 | 4.41 | 6.24E+00 |
| ENSGALG00000012338 | BDH2 | 4 | 61363428 | 61376731 | - | -1.13 | 7.79E-07 | 13.97 | 1.82E+01 |
| ENSGALG00000012552 | TOM1 | 1 | 52167132 | 52187809 | - | -1.15 | 1.65E-04 | 1.40 | 1.42E+00 |
| ENSGALG00000032413 | NADK | 21 | 1970069 | 1990004 | + | -1.15 | 2.82E-08 | 63.39 | 6.56E+01 |
| ENSGALG00000000681 | PAK1 | 1 | 193148137 | 193175592 | + | -1.15 | 3.74E-09 | 3.59 | 2.70E+00 |
| ENSGALG00000005430 | ALS2CL | 2 | 3259507 | 3285387 | - | -1.15 | 7.78E-05 | 2.54 | 1.96E+00 |
| ENSGALG00000003670 | MAFB | 20 | 4836734 | 4839401 | - | -1.15 | 2.49E-07 | 11.12 | 1.47E+01 |
| ENSGALG00000014958 | ANKDD1B | Z | 23845453 | 23867229 | - | -1.16 | 7.43E-04 | 1.23 | 2.37E+00 |
| ENSGALG00000039432 | P2RY13 | 9 | 23629503 | 23631209 | + | -1.17 | 8.22E-04 | 5.80 | 3.82E+00 |
| ENSGALG00000016834 | MCF2L | 1 | 137606558 | 137750320 | - | -1.17 | 3.64E-04 | 3.25 | 3.82E+00 |
| ENSGALG00000011499 | SCARB2 | 4 | 50249539 | 50270243 | + | -1.18 | 4.00E-07 | 10.01 | 1.20E+01 |
| ENSGALG00000026188 | ENSGALG00000026188 | 14 | 12176496 | 12188297 | + | -1.19 | 3.88E-06 | 4.19 | 2.81E+00 |
| ENSGALG00000011291 | NR1D2 | 2 | 37345920 | 37368540 | + | -1.20 | 8.90E-04 | 2.68 | 2.51E+00 |
| ENSGALG00000005065 | PLA2G4A | 8 | 9797493 | 9862645 | - | -1.21 | 1.56E-06 | 4.93 | 4.47E+00 |
| ENSGALG00000027483 | GLRX | Z | 57343843 | 57352236 | + | -1.21 | 8.71E-06 | 7.69 | 1.16E+01 |
| ENSGALG00000002105 | FOXI1 | 13 | 3949132 | 3951294 | - | -1.22 | 2.14E-04 | 4.21 | 5.05E+00 |
| ENSGALG00000015297 | FOXO3 | 3 | 67450300 | 67533242 | - | -1.22 | 4.13E-05 | 0.84 | 1.00E+00 |
| ENSGALG00000030005 | IGSF1 | 20 | 65206 | 68264 | + | -1.23 | 7.99E-05 | 4.50 | 4.20E+00 |
| ENSGALG00000002655 | ITGAV | 7 | 1319204 | 1364895 | - | -1.23 | 3.69E-05 | 16.49 | 1.66E+01 |
| ENSGALG00000015020 | TMEM171 | Z | 24907673 | 24918495 | - | -1.23 | 6.58E-05 | 1.76 | 2.22E+00 |
| ENSGALG00000034528 | SNTB1 | 2 | 137075115 | 137183189 | - | -1.24 | 7.65E-04 | 5.85 | 6.76E+00 |
| ENSGALG00000031284 | PTPRM | 2 | 99160698 | 99619495 | - | -1.26 | 9.30E-04 | 1.05 | 7.48E-01 |
| ENSGALG00000016419 | ASAP2 | 3 | 96512108 | 96596917 | + | -1.27 | 7.13E-04 | 0.89 | 1.21E+00 |
| ENSGALG00000012074 | ALPK1 | 4 | 57505958 | 57539448 | - | -1.27 | 1.20E-04 | 0.55 | 6.11E-01 |
| ENSGALG00000009031 | SESTD1 | 7 | 15097026 | 15133589 | + | -1.29 | 2.59E-04 | 5.77 | 5.89E+00 |
| ENSGALG00000010081 | SH3D19 | 4 | 33497816 | 33524141 | - | -1.30 | 5.79E-07 | 5.68 | 3.06E+00 |
| ENSGALG00000011278 | KAT2B | 2 | 35988108 | 36027652 | + | -1.30 | 1.56E-06 | 5.14 | 5.17E+00 |
| ENSGALG00000013780 | AIG1 | 3 | 52657464 | 52777747 | - | -1.32 | 8.94E-07 | 5.84 | 5.44E+00 |
| ENSGALG00000009240 | NFE2L2 | 7 | 15867686 | 15883497 | + | -1.32 | 2.02E-05 | 12.67 | 1.03E+01 |
| ENSGALG00000013624 | FAM65B | 2 | 90472344 | 90506067 | + | -1.33 | 8.33E-04 | 0.69 | 1.44E+00 |
| ENSGALG00000007522 | ABCC3 | 18 | 10231908 | 10275081 | - | -1.33 | 3.37E-05 | 3.92 | 2.29E+00 |
| ENSGALG00000023517 | AGPAT2 | 17 | 8222165 | 8229069 | + | -1.34 | 3.31E-07 | 7.10 | 4.97E+00 |
| ENSGALG00000005958 | TRIM66 | 5 | 9747967 | 9778578 | + | -1.35 | 2.10E-04 | 0.47 | 4.90E-01 |
| ENSGALG00000017199 | MAML2 | 1 | 184996598 | 185204663 | + | -1.36 | 3.28E-04 | 0.29 | 3.29E-01 |
| ENSGALG00000016833 | F7 | 1 | 137595069 | 137603553 | - | -1.36 | 6.63E-04 | 0.74 | 5.01E-01 |
| ENSGALG00000002529 | ENSGALG00000002529 | Z | 73104188 | 73137042 | + | -1.37 | 2.31E-04 | 3.85 | 6.01E+00 |
| ENSGALG00000016736 | CYFIP1 | 1 | 130435494 | 130485774 | + | -1.38 | 8.45E-11 | 8.63 | 1.11E+01 |
| ENSGALG00000017150 | ENSGALG00000017150 | 1 | 179517182 | 179576104 | + | -1.39 | 3.48E-04 | 0.88 | 2.07E+00 |
| ENSGALG00000016996 | ITM2B | 1 | 168710780 | 168736807 | + | -1.42 | 4.34E-07 | 29.61 | 5.04E+01 |
| ENSGALG00000031495 | TRIO | 2 | 76545808 | 76794884 | - | -1.43 | 1.15E-09 | 1.79 | 9.65E-01 |
| ENSGALG00000001231 | SAMHD1 | 20 | 632272 | 658022 | - | -1.45 | 2.28E-21 | 23.28 | 2.13E+01 |
| ENSGALG00000012763 | GNPTAB | 1 | 55573823 | 55614936 | + | -1.46 | 4.64E-04 | 6.46 | 5.34E+00 |
| ENSGALG00000006407 | ENSGALG00000006407 | 5 | 13059342 | 13065622 | + | -1.46 | 3.83E-04 | 7.53 | 5.28E+00 |
| ENSGALG00000002055 | HES4 | 21 | 2797270 | 2799502 | + | -1.47 | 2.77E-05 | 2.15 | 1.58E+00 |
| ENSGALG00000030866 | CPQ | 2 | 127544122 | 127708621 | + | -1.47 | 4.05E-06 | 2.41 | 3.37E+00 |
| ENSGALG00000010412 | WWTR1 | 9 | 23898460 | 23938545 | + | -1.47 | 1.37E-06 | 1.14 | 2.04E+00 |
| ENSGALG00000016971 | TSC22D1 | 1 | 167406458 | 167484232 | - | -1.48 | 1.17E-09 | 6.88 | 5.35E+00 |
| ENSGALG00000026970 | ENSGALG00000026970 | 5 | 1549219 | 1550524 | + | -1.48 | 1.84E-06 | 26.78 | 3.36E+01 |
| ENSGALG00000029533 | LRP5 | 5 | 16375981 | 16504146 | + | -1.51 | 1.42E-16 | 1.76 | 1.97E+00 |
| ENSGALG00000011446 | TNFAIP2 | 5 | 50222277 | 50231944 | + | -1.51 | 1.88E-04 | 1.65 | 1.47E+00 |
| ENSGALG00000006503 | VDAC1 | 13 | 16119877 | 16134982 | + | -1.52 | 1.37E-06 | 8.28 | 7.08E+00 |
| ENSGALG00000040770 | TNRC18 | 14 | 4101243 | 4145400 | - | -1.54 | 1.45E-05 | 1.12 | 6.07E-01 |
| ENSGALG00000028924 | S100A16 | 25 | 1867721 | 1868128 | + | -1.54 | 8.86E-06 | 3.63 | 2.20E+00 |
| ENSGALG00000009655 | AADAT | 4 | 25423919 | 25438950 | + | -1.55 | 1.84E-06 | 1.02 | 1.23E+00 |
| ENSGALG00000003939 | ENSGALG00000003939 | 28 | 4398876 | 4401204 | - | -1.56 | 2.50E-07 | 1.26 | 1.56E+00 |
| ENSGALG00000014998 | ENSGALG00000014998 | 2 | 103216424 | 103248307 | + | -1.56 | 5.68E-05 | 0.67 | 4.89E-01 |
| ENSGALG00000038043 | HECTD2 | 6 | 19794881 | 19823165 | + | -1.57 | 1.62E-04 | 0.39 | 5.04E-01 |
| ENSGALG00000008631 | TYRO3 | 5 | 24921251 | 24960510 | + | -1.58 | 2.34E-16 | 2.98 | 2.15E+00 |
| ENSGALG00000012017 | DAAM1 | 5 | 54906321 | 54984749 | - | -1.58 | 8.40E-06 | 0.62 | 3.72E-01 |
| ENSGALG00000034289 | SLC41A3 | 12 | 10499369 | 10520263 | - | -1.58 | 1.27E-06 | 1.53 | 7.23E-01 |
| ENSGALG00000010158 | KANK1 | Z | 25816644 | 25934004 | + | -1.60 | 2.11E-07 | 0.49 | 5.75E-01 |
| ENSGALG00000016480 | SDC1 | 3 | 102005547 | 102025143 | - | -1.61 | 1.79E-05 | 12.55 | 6.49E+00 |
| ENSGALG00000029102 | PXYLP1 | 9 | 6796763 | 6897692 | + | -1.61 | 1.21E-08 | 0.67 | 1.13E+00 |
| ENSGALG00000006942 | PIK3IP1 | 15 | 9093095 | 9095888 | + | -1.61 | 4.75E-05 | 3.30 | 1.24E+01 |
| ENSGALG00000003855 | SRC | 20 | 5212145 | 5229299 | + | -1.61 | 1.60E-07 | 0.60 | 5.41E-01 |
| ENSGALG00000002038 | DENND2C | 26 | 3872935 | 3887414 | - | -1.61 | 8.42E-11 | 1.07 | 8.55E-01 |
| ENSGALG00000013594 | PARD6G | 2 | 90091922 | 90150000 | - | -1.61 | 2.47E-12 | 3.43 | 2.68E+00 |
| ENSGALG00000030276 | ENSGALG00000030276 | 6 | 29009732 | 29063676 | - | -1.62 | 5.97E-04 | 1.03 | 4.28E-01 |
| ENSGALG00000001617 | CAMKK1 | 19 | 3300331 | 3382167 | + | -1.62 | 1.70E-06 | 1.21 | 7.91E-01 |
| ENSGALG00000023689 | ASS1 | 17 | 6346895 | 6367468 | + | -1.62 | 4.50E-08 | 13.76 | 9.73E+00 |
| ENSGALG00000008930 | B3GNT2 | 3 | 9067300 | 9084370 | + | -1.63 | 5.29E-04 | 6.33 | 5.03E+00 |
| ENSGALG00000002294 | SEMA6A | Z | 72560203 | 72672912 | + | -1.65 | 5.59E-04 | 0.14 | 1.71E-01 |
| ENSGALG00000009898 | GAB1 | 4 | 30725096 | 30824645 | + | -1.66 | 1.13E-16 | 0.84 | 1.08E+00 |
| ENSGALG00000003144 | TRIM25 | 18 | 6353980 | 6361372 | - | -1.67 | 5.34E-10 | 3.14 | 3.81E+00 |
| ENSGALG00000003136 | IKZF2 | 7 | 3645916 | 3763784 | - | -1.70 | 9.55E-04 | 0.25 | 2.15E-01 |
| ENSGALG00000001331 | ST14 | 24 | 1562614 | 1582952 | + | -1.70 | 1.03E-07 | 2.44 | 2.50E+00 |
| ENSGALG00000003767 | NKD1 | 11 | 6364520 | 6468658 | - | -1.70 | 7.21E-05 | 0.36 | 2.18E-01 |
| ENSGALG00000014689 | FAM131B | 1 | 77586269 | 77592786 | + | -1.70 | 2.05E-04 | 0.21 | 6.82E-01 |
| ENSGALG00000032640 | CRIM1 | 3 | 32366696 | 32528059 | - | -1.72 | 1.55E-08 | 1.09 | 1.63E+00 |
| ENSGALG00000034493 | DCLK3 | 2 | 46064049 | 46088568 | - | -1.72 | 4.71E-07 | 1.82 | 1.90E+00 |
| ENSGALG00000033195 | IGF2BP2 | 9 | 4414719 | 4430179 | + | -1.72 | 2.59E-06 | 0.67 | 8.99E-01 |
| ENSGALG00000006805 | ENSGALG00000006805 | 9 | 11576135 | 11588043 | - | -1.72 | 1.09E-08 | 13.72 | 1.46E+01 |
| ENSGALG00000038933 | FABP7 | 3 | 61382563 | 61385979 | - | -1.73 | 6.44E-09 | 3.69 | 2.15E+00 |
| ENSGALG00000041255 | ADAM12 | 6 | 32619954 | 32790667 | - | -1.74 | 9.00E-05 | 0.16 | 2.79E-01 |
| ENSGALG00000007744 | CASS4 | 20 | 12201193 | 12217115 | - | -1.75 | 9.20E-04 | 0.39 | 2.51E+00 |
| ENSGALG00000016224 | MAOA | 1 | 111259525 | 111332024 | - | -1.76 | 4.55E-07 | 2.24 | 3.77E+00 |
| ENSGALG00000016486 | HS1BP3 | 3 | 102322246 | 102366030 | - | -1.76 | 2.39E-15 | 0.74 | 8.89E-01 |
| ENSGALG00000011717 | ENSGALG00000011717 | 1 | 47884753 | 47909098 | + | -1.76 | 1.13E-06 | 0.35 | 3.06E-01 |
| ENSGALG00000011982 | PDE5 | 4 | 55000129 | 55059970 | + | -1.76 | 8.35E-10 | 1.77 | 2.39E+00 |
| ENSGALG00000014914 | SNX18 | Z | 16452976 | 16470369 | + | -1.76 | 7.07E-06 | 0.96 | 1.61E+00 |
| ENSGALG00000033416 | HIC2 | 15 | 239932 | 308559 | - | -1.76 | 1.07E-04 | 0.40 | 4.45E-01 |
| ENSGALG00000026600 | C1orf198 | 3 | 40659667 | 40679553 | - | -1.76 | 3.66E-04 | 1.06 | 1.25E+00 |
| ENSGALG00000027345 | SLC46A2 | Z | 65849819 | 65854890 | + | -1.76 | 1.16E-06 | 1.48 | 1.77E+00 |
| ENSGALG00000031425 | PMP22 | 18 | 2352300 | 2365897 | + | -1.77 | 7.43E-07 | 1.30 | 2.26E+00 |
| ENSGALG00000023780 | TREM-B2 | 26 | 4755757 | 4761750 | - | -1.79 | 1.19E-04 | 0.66 | 9.22E-01 |
| ENSGALG00000043128 | ARHGEF10L | 21 | 117333 | 138142 | - | -1.82 | 8.61E-12 | 1.12 | 7.89E-01 |
| ENSGALG00000041650 | DUSP7 | 12 | 254374 | 261731 | + | -1.82 | 1.02E-16 | 15.23 | 1.31E+01 |
| ENSGALG00000003507 | TECPR1 | 14 | 1112494 | 1131672 | - | -1.83 | 9.20E-17 | 1.09 | 1.23E+00 |
| ENSGALG00000014824 | PTGER4 | Z | 12839413 | 12850084 | + | -1.83 | 3.20E-04 | 0.18 | 2.10E-01 |
| ENSGALG00000009480 | DOCK4 | 1 | 27184787 | 27262852 | + | -1.85 | 2.16E-07 | 0.75 | 1.09E+00 |
| ENSGALG00000037203 | CTNNB1 | 2 | 43601202 | 43622659 | + | -1.85 | 4.03E-08 | 13.31 | 2.72E+01 |
| ENSGALG00000042259 | DOCK1 | 6 | 32938446 | 33212336 | + | -1.85 | 3.70E-12 | 0.77 | 7.41E-01 |
| ENSGALG00000013134 | ENSGALG00000013134 | 1 | 64403222 | 64534173 | + | -1.85 | 1.46E-06 | 0.98 | 7.21E-01 |
| ENSGALG00000005714 | PKD1 | 14 | 6339857 | 6422473 | - | -1.87 | 1.54E-05 | 0.17 | 2.74E-01 |
| ENSGALG00000005849 | PPDPF | 20 | 9221057 | 9223364 | - | -1.87 | 1.46E-06 | 1.77 | 2.68E+00 |
| ENSGALG00000025898 | SMAD6 | 10 | 18699479 | 18727091 | + | -1.88 | 1.36E-05 | 1.08 | 3.57E-01 |
| ENSGALG00000034007 | ITGA6 | 7 | 17799837 | 17838429 | - | -1.88 | 2.43E-10 | 3.39 | 8.76E+00 |
| ENSGALG00000030275 | ENSGALG00000030275 | 3 | 61364647 | 61379970 | - | -1.88 | 5.60E-10 | 2.73 | 2.32E+00 |
| ENSGALG00000016776 | TBC1D8 | 1 | 133434844 | 133489335 | - | -1.89 | 2.85E-08 | 1.07 | 9.14E-01 |
| ENSGALG00000015177 | GNAQ | Z | 37785034 | 37901983 | - | -1.89 | 1.32E-08 | 1.23 | 3.44E+00 |
| ENSGALG00000009809 | MAML3 | 4 | 29615758 | 29825230 | - | -1.89 | 4.09E-07 | 0.22 | 2.19E-01 |
| ENSGALG00000010579 | STON2 | 5 | 40913516 | 40968803 | - | -1.92 | 4.94E-08 | 0.59 | 7.30E-01 |
| ENSGALG00000033286 | ARSJ | 4 | 56815153 | 56854281 | + | -1.92 | 1.35E-08 | 0.49 | 3.93E-01 |
| ENSGALG00000008542 | ADD3 | 6 | 25742152 | 25827920 | + | -1.92 | 5.42E-15 | 16.06 | 1.02E+01 |
| ENSGALG00000011104 | GCG | 7 | 21234151 | 21245324 | + | -1.92 | 2.80E-08 | 0.91 | 2.13E+00 |
| ENSGALG00000037413 | IL7 | 2 | 120829374 | 120835622 | - | -1.94 | 1.78E-04 | 0.27 | 6.37E-01 |
| ENSGALG00000043142 | PCDHA12 | 13 | 704847 | 869303 | - | -1.95 | 3.78E-04 | 0.11 | 1.20E-01 |
| ENSGALG00000005042 | IQSEC1 | 12 | 5275752 | 5383940 | - | -1.95 | 1.52E-07 | 1.15 | 1.33E+00 |
| ENSGALG00000003601 | ATP2B4 | 26 | 5209970 | 5248800 | + | -1.95 | 5.39E-16 | 1.08 | 8.68E-01 |
| ENSGALG00000009012 | ZNF385B | 7 | 14980081 | 15028066 | + | -1.96 | 4.77E-04 | 0.11 | 2.35E-01 |
| ENSGALG00000021451 | ENSGALG00000021451 | 11 | 1103753 | 1106406 | - | -1.97 | 4.08E-06 | 1.06 | 6.92E-01 |
| ENSGALG00000011190 | ENSGALG00000011190 | 4 | 46742443 | 46743346 | - | -1.97 | 9.26E-04 | 1.13 | 1.35E+00 |
| ENSGALG00000016412 | MBOAT2 | 3 | 96358528 | 96449224 | - | -1.97 | 1.12E-04 | 0.45 | 1.56E+00 |
| ENSGALG00000040557 | TFEC | 1 | 25289184 | 25351175 | + | -2.00 | 6.44E-05 | 0.63 | 3.34E-01 |
| ENSGALG00000043618 | SLC40A1 | 7 | 353851 | 368636 | + | -2.00 | 3.47E-04 | 14.20 | 4.89E+01 |
| ENSGALG00000015969 | FAM135A | 3 | 82962404 | 83039760 | - | -2.00 | 1.10E-07 | 0.92 | 8.39E-01 |
| ENSGALG00000006598 | SORL1 | 24 | 3488615 | 3526611 | - | -2.00 | 2.56E-16 | 0.28 | 2.19E-01 |
| ENSGALG00000017302 | SLCO2B1 | 1 | 196123765 | 196155122 | - | -2.01 | 2.78E-07 | 2.17 | 2.72E+00 |
| ENSGALG00000016886 | FARP1 | 1 | 144902425 | 145084130 | - | -2.01 | 8.49E-19 | 1.41 | 1.01E+00 |
| ENSGALG00000007012 | ARRDC4 | 10 | 16471589 | 16478235 | + | -2.01 | 2.17E-05 | 2.36 | 2.23E+00 |
| ENSGALG00000015464 | PTGFRN | 1 | 91994160 | 92037790 | + | -2.02 | 1.04E-04 | 0.45 | 1.11E+00 |
| ENSGALG00000038136 | ENSGALG00000038136 | 11 | 100104 | 101117 | - | -2.02 | 5.52E-10 | 13.35 | 2.07E+01 |
| ENSGALG00000010352 | ARHGEF26 | 9 | 23309859 | 23353078 | - | -2.03 | 1.51E-04 | 1.78 | 2.81E-01 |
| ENSGALG00000008912 | ABCB1 | 2 | 20693000 | 20781828 | - | -2.04 | 3.27E-04 | 0.22 | 1.20E+00 |
| ENSGALG00000032736 | MGLL | 12 | 9622177 | 9678239 | + | -2.04 | 2.90E-04 | 0.60 | 6.62E-01 |
| ENSGALG00000009901 | DYRK2 | 1 | 34899306 | 34909399 | + | -2.05 | 2.46E-05 | 0.57 | 4.70E-01 |
| ENSGALG00000013605 | LRRC16A | 2 | 90220685 | 90394975 | - | -2.05 | 3.71E-06 | 0.73 | 1.08E+00 |
| ENSGALG00000010505 | BEND5 | 8 | 23084517 | 23113939 | - | -2.06 | 1.43E-06 | 0.47 | 1.03E+00 |
| ENSGALG00000015307 | ABI3BP | 1 | 84985337 | 85117092 | - | -2.06 | 2.45E-05 | 0.45 | 2.47E-01 |
| ENSGALG00000001483 | ENSGALG00000001483 | 21 | 2097421 | 2103217 | + | -2.07 | 1.62E-15 | 2.44 | 3.05E+00 |
| ENSGALG00000023120 | SULT1B1 | 4 | 52137022 | 52139535 | - | -2.07 | 5.58E-13 | 1.49 | 2.10E+00 |
| ENSGALG00000038652 | ENSGALG00000038652 | 3 | 88760377 | 88772621 | + | -2.08 | 7.91E-12 | 5.53 | 4.03E+00 |
| ENSGALG00000043661 | ENSGALG00000043661 | 5 | 48297347 | 48396584 | + | -2.09 | 1.19E-04 | 0.06 | 2.41E-01 |
| ENSGALG00000029175 | EFNB2 | 1 | 141049589 | 141090557 | + | -2.09 | 5.83E-06 | 1.69 | 6.97E-01 |
| ENSGALG00000006502 | PCGF5 | 6 | 19720931 | 19745244 | + | -2.09 | 9.52E-07 | 0.72 | 8.75E-01 |
| ENSGALG00000005024 | EFCC1 | 12 | 5149129 | 5193490 | - | -2.10 | 5.92E-04 | 0.13 | 4.00E-01 |
| ENSGALG00000006325 | ENSGALG00000006325 | 12 | 10728663 | 10770062 | + | -2.12 | 1.75E-05 | 0.28 | 3.87E-01 |
| ENSGALG00000011295 | SOCS2 | 1 | 44892298 | 44893378 | + | -2.13 | 8.64E-05 | 0.96 | 4.56E-01 |
| ENSGALG00000012816 | SLC22A23 | 2 | 66392888 | 66487217 | + | -2.13 | 1.87E-06 | 0.50 | 2.25E-01 |
| ENSGALG00000009926 | HAAO | 3 | 24178579 | 24209441 | - | -2.14 | 3.37E-05 | 1.68 | 1.12E+00 |
| ENSGALG00000040651 | IGF1R | 10 | 16697772 | 16838212 | - | -2.17 | 2.40E-18 | 0.44 | 4.94E-01 |
| ENSGALG00000013803 | GPR126 | 3 | 53020301 | 53127121 | - | -2.18 | 9.67E-07 | 3.32 | 1.31E+00 |
| ENSGALG00000014930 | ENC1 | Z | 24273768 | 24275537 | + | -2.19 | 7.03E-04 | 0.14 | 3.03E-02 |
| ENSGALG00000014346 | TSPAN9 | 1 | 75380490 | 75521578 | - | -2.20 | 5.19E-10 | 1.25 | 1.12E+00 |
| ENSGALG00000004593 | SULF2 | 20 | 6110103 | 6166886 | - | -2.20 | 7.26E-11 | 2.80 | 2.80E+00 |
| ENSGALG00000039735 | ENSGALG00000039735 | 5 | 48398823 | 48416449 | + | -2.21 | 6.10E-08 | 0.46 | 1.37E+00 |
| ENSGALG00000002326 | ENSGALG00000002326 | Z | 8885737 | 8943793 | + | -2.27 | 6.68E-17 | 1.31 | 1.52E+00 |
| ENSGALG00000015770 | APP | 1 | 102610038 | 102809834 | - | -2.27 | 4.65E-14 | 31.62 | 3.04E+01 |
| ENSGALG00000043511 | ARHGAP32 | 24 | 1149631 | 1160969 | - | -2.28 | 8.13E-09 | 0.13 | 1.47E-01 |
| ENSGALG00000030966 | MAPK13 | 26 | 164121 | 175093 | + | -2.28 | 8.95E-07 | 0.38 | 7.64E-01 |
| ENSGALG00000006602 | SYT8 | 5 | 14108059 | 14112076 | - | -2.29 | 6.36E-12 | 2.35 | 1.14E+00 |
| ENSGALG00000008886 | ZNF618 | 17 | 1550305 | 1694877 | - | -2.30 | 3.10E-05 | 0.16 | 1.13E-01 |
| ENSGALG00000005305 | ACKR2 | 2 | 2096559 | 2103345 | + | -2.30 | 1.34E-10 | 1.20 | 9.17E-01 |
| ENSGALG00000026677 | F10 | 1 | 137579845 | 137592401 | - | -2.31 | 9.54E-08 | 2.96 | 6.23E-01 |
| ENSGALG00000030065 | TENM3 | 4 | 40334424 | 40645412 | - | -2.32 | 1.53E-04 | 0.13 | 4.07E-01 |
| ENSGALG00000028928 | LCAT | 11 | 1094450 | 1100820 | - | -2.32 | 6.64E-06 | 0.98 | 1.11E+00 |
| ENSGALG00000011078 | PTPN13 | 4 | 46127455 | 46159442 | - | -2.32 | 9.40E-12 | 0.52 | 8.95E-01 |
| ENSGALG00000019489 | CHST9 | 2 | 104761260 | 104847802 | - | -2.33 | 6.36E-06 | 0.37 | 4.01E-01 |
| ENSGALG00000003802 | OTUD7A | 10 | 5449521 | 5483094 | + | -2.33 | 1.65E-04 | 0.17 | 1.61E-01 |
| ENSGALG00000016781 | MAP4K4 | 1 | 133742880 | 133800008 | + | -2.36 | 1.09E-12 | 2.19 | 1.57E+00 |
| ENSGALG00000001314 | PTGS1 | 17 | 9247984 | 9255289 | + | -2.38 | 5.30E-06 | 0.09 | 2.79E-01 |
| ENSGALG00000009252 | ENSGALG00000009252 | 9 | 19600701 | 19636193 | + | -2.39 | 5.37E-20 | 0.42 | 3.52E-01 |
| ENSGALG00000008701 | XDH | 3 | 4482183 | 4522989 | + | -2.39 | 1.48E-36 | 2.22 | 2.97E+00 |
| ENSGALG00000008518 | ENSGALG00000008518 | 17 | 2429856 | 2435388 | + | -2.39 | 4.00E-18 | 2.58 | 6.08E+00 |
| ENSGALG00000009803 | MGST2 | 4 | 29603081 | 29611243 | + | -2.39 | 5.04E-10 | 1.36 | 1.04E+00 |
| ENSGALG00000023089 | SSBP2 | Z | 63521369 | 63687448 | + | -2.41 | 7.63E-08 | 0.29 | 6.09E-01 |
| ENSGALG00000016059 | ETS2 | 1 | 107802189 | 107815381 | + | -2.48 | 4.55E-20 | 3.94 | 3.71E+00 |
| ENSGALG00000043688 | SH3TC2 | 13 | 8592131 | 8600567 | + | -2.48 | 6.63E-16 | 0.38 | 2.97E-01 |
| ENSGALG00000015056 | ENSGALG00000015056 | 2 | 103493136 | 103529171 | + | -2.50 | 4.21E-30 | 1.31 | 2.24E+00 |
| ENSGALG00000009759 | SLC16A7 | 1 | 32086287 | 32120482 | + | -2.51 | 1.81E-06 | 0.05 | 2.63E-01 |
| ENSGALG00000007841 | DNAH7 | 7 | 9683028 | 9793346 | - | -2.51 | 9.39E-06 | 0.00 | 1.80E-02 |
| ENSGALG00000029270 | GATA3 | 1 | 4402132 | 4591692 | + | -2.51 | 3.75E-04 | 0.07 | 0.00E+00 |
| ENSGALG00000007218 | MB21D2 | 9 | 13300318 | 13348379 | + | -2.52 | 3.07E-04 | 0.03 | 3.64E-02 |
| ENSGALG00000005805 | PLCD1 | 2 | 4635397 | 4674388 | - | -2.54 | 1.21E-16 | 1.85 | 1.31E+00 |
| ENSGALG00000041717 | KLF3 | 4 | 69980272 | 70000045 | - | -2.56 | 2.88E-07 | 0.22 | 8.07E-01 |
| ENSGALG00000002479 | MAT1A | 6 | 4436646 | 4453363 | + | -2.57 | 1.04E-07 | 0.69 | 3.57E-01 |
| ENSGALG00000043044 | IL1R1 | 1 | 133879807 | 133899318 | + | -2.59 | 8.06E-17 | 0.91 | 1.71E+00 |
| ENSGALG00000016602 | ENSGALG00000016602 | 1 | 124175179 | 124319202 | + | -2.63 | 1.15E-08 | 0.15 | 7.46E-01 |
| ENSGALG00000028466 | ENSGALG00000028466 | 11 | 1776707 | 1780905 | - | -2.64 | 4.25E-07 | 0.47 | 1.90E-01 |
| ENSGALG00000029940 | il-1beta | 22 | 4616890 | 4618940 | - | -2.64 | 2.57E-07 | 0.56 | 3.40E-01 |
| ENSGALG00000005266 | BEAN1 | 11 | 11619893 | 11657333 | - | -2.66 | 1.36E-11 | 0.44 | 3.15E-01 |
| ENSGALG00000002553 | PCDH1 | 13 | 2037709 | 2061000 | + | -2.67 | 2.51E-11 | 0.11 | 1.39E-01 |
| ENSGALG00000004376 | KCNJ2 | 18 | 8259055 | 8265799 | + | -2.68 | 2.41E-08 | 0.78 | 2.53E-01 |
| ENSGALG00000030767 | ENSGALG00000030767 | 2 | 111387914 | 111406662 | - | -2.70 | 8.94E-07 | 0.06 | 1.68E-01 |
| ENSGALG00000027036 | PKIB | 3 | 61397818 | 61403187 | - | -2.71 | 5.56E-11 | 0.66 | 6.78E-01 |
| ENSGALG00000007839 | NCAM1 | 24 | 5780707 | 5862536 | - | -2.72 | 2.40E-05 | 0.05 | 2.89E-01 |
| ENSGALG00000023824 | ENSGALG00000023824 | 6 | 19165336 | 19177973 | + | -2.72 | 2.76E-04 | 0.17 | 1.29E-01 |
| ENSGALG00000011686 | SEMA5B | 7 | 26700264 | 26882259 | - | -2.74 | 4.17E-13 | 0.11 | 1.83E-01 |
| ENSGALG00000033171 | TGM4 | 2 | 43317611 | 43328122 | - | -2.75 | 1.80E-04 | 3.48 | 3.11E-01 |
| ENSGALG00000007174 | TNFSF15 | 17 | 3061247 | 3074511 | - | -2.75 | 9.01E-08 | 0.54 | 1.49E-01 |
| ENSGALG00000032363 | GPR141 | 2 | 46412221 | 46429402 | - | -2.75 | 6.55E-09 | 0.26 | 3.33E-01 |
| ENSGALG00000015345 | ENSGALG00000015345 | 3 | 68326420 | 68390064 | - | -2.79 | 1.16E-13 | 0.28 | 3.42E-01 |
| ENSGALG00000004420 | SDK1 | 14 | 3530847 | 3884236 | + | -2.80 | 4.09E-09 | 0.09 | 6.02E-02 |
| ENSGALG00000013785 | GNAL | 2 | 97561465 | 97675905 | - | -2.80 | 1.77E-08 | 0.24 | 7.28E-01 |
| ENSGALG00000039334 | ENSGALG00000039334 | 5 | 689869 | 712052 | - | -2.80 | 2.57E-07 | 0.17 | 3.68E-01 |
| ENSGALG00000008907 | PLCB4 | 3 | 13955750 | 14046844 | - | -2.81 | 1.59E-09 | 0.11 | 3.66E-01 |
| ENSGALG00000011744 | ENSGALG00000011744 | 7 | 27566419 | 28008798 | + | -2.81 | 1.02E-16 | 0.15 | 1.42E-01 |
| ENSGALG00000010934 | ENSGALG00000010934 | 8 | 27404833 | 27470245 | + | -2.82 | 5.27E-08 | 0.21 | 2.30E-01 |
| ENSGALG00000003876 | TIMD4 | 13 | 11469602 | 11484197 | + | -2.82 | 1.50E-11 | 66.64 | 2.06E+01 |
| ENSGALG00000004741 | EPHB2 | 21 | 5954684 | 6063248 | - | -2.85 | 8.61E-24 | 0.31 | 2.98E-01 |
| ENSGALG00000009392 | TLR5 | 3 | 17869807 | 17872392 | + | -2.85 | 4.08E-17 | 0.30 | 1.45E-01 |
| ENSGALG00000007132 | ACOX2 | 12 | 11864373 | 11878164 | - | -2.88 | 5.44E-16 | 0.30 | 3.64E-01 |
| ENSGALG00000035504 | ER81 | 2 | 27328222 | 27392423 | - | -2.89 | 4.25E-12 | 0.37 | 9.70E-01 |
| ENSGALG00000027209 | SPIC | 1 | 55757636 | 55763936 | - | -2.93 | 2.28E-05 | 2.51 | 7.10E-02 |
| ENSGALG00000032534 | OBSCN | 2 | 2110152 | 2290343 | - | -2.96 | 4.23E-06 | 0.00 | 2.10E-03 |
| ENSGALG00000017208 | AMOTL1 | 1 | 185571024 | 185628252 | - | -2.96 | 3.64E-15 | 0.25 | 1.45E-01 |
| ENSGALG00000010316 | FRAS1 | 4 | 35018318 | 35181390 | - | -2.98 | 4.48E-14 | 0.04 | 1.76E-02 |
| ENSGALG00000014267 | RBM47 | 4 | 69357333 | 69363626 | + | -3.02 | 5.45E-22 | 0.53 | 5.99E-01 |
| ENSGALG00000013994 | TAAR1 | 3 | 57019995 | 57020993 | + | -3.10 | 2.33E-12 | 1.40 | 1.13E+00 |
| ENSGALG00000029842 | SLCO5A1 | 2 | 116787853 | 116850398 | - | -3.11 | 2.63E-05 | 0.02 | 0.00E+00 |
| ENSGALG00000003323 | OSGIN1 | 11 | 2658956 | 2694119 | - | -3.14 | 6.77E-26 | 0.16 | 2.72E-01 |
| ENSGALG00000038000 | CX3CR1 | 2 | 44545657 | 44555354 | + | -3.17 | 1.57E-10 | 1.29 | 1.59E+00 |
| ENSGALG00000041978 | k123 | 1 | 3465887 | 3476005 | + | -3.19 | 1.43E-14 | 7.44 | 2.78E+00 |
| ENSGALG00000002888 | CASZ1 | 21 | 3822317 | 3944648 | - | -3.21 | 1.38E-13 | 0.04 | 2.76E-02 |
| ENSGALG00000003742 | CPAMD8 | 28 | 4229272 | 4253848 | + | -3.22 | 8.88E-12 | 0.05 | 7.60E-02 |
| ENSGALG00000003886 | SGCD | 13 | 11524677 | 11663416 | - | -3.25 | 1.01E-08 | 0.08 | 2.74E-01 |
| ENSGALG00000013468 | TLR3 | 4 | 61707202 | 61720669 | + | -3.26 | 1.03E-18 | 0.74 | 1.07E+00 |
| ENSGALG00000015683 | GNG10 | Z | 66386557 | 66392556 | - | -3.27 | 1.78E-25 | 4.01 | 3.95E+00 |
| ENSGALG00000008953 | AASS | 1 | 22867946 | 22895501 | + | -3.27 | 6.61E-28 | 0.48 | 1.02E+00 |
| ENSGALG00000012171 | GPR39 | 7 | 29857698 | 29921974 | + | -3.27 | 6.37E-08 | 0.08 | 4.33E-01 |
| ENSGALG00000040444 | SEMA3C | 1 | 11044256 | 11159489 | + | -3.31 | 2.79E-15 | 0.12 | 5.05E-01 |
| ENSGALG00000020561 | ENSGALG00000020561 | Z | 16555609 | 16594096 | - | -3.32 | 3.91E-12 | 0.08 | 1.36E-01 |
| ENSGALG00000017246 | ME3 | 1 | 188917283 | 189035434 | + | -3.33 | 4.29E-15 | 0.21 | 1.11E-01 |
| ENSGALG00000009805 | PPM1H | 1 | 33089980 | 33218417 | - | -3.33 | 6.08E-14 | 0.27 | 7.01E-02 |
| ENSGALG00000012395 | PLEKHG1 | 3 | 48760267 | 48790770 | + | -3.34 | 3.52E-33 | 0.37 | 5.23E-01 |
| ENSGALG00000010866 | AREG | 4 | 45060436 | 45066903 | + | -3.40 | 9.20E-07 | 0.03 | 0.00E+00 |
| ENSGALG00000042317 | P2RY2 | 1 | 195388851 | 195389927 | + | -3.40 | 1.34E-07 | 0.11 | 9.97E-02 |
| ENSGALG00000000584 | ENSGALG00000000584 | 26 | 375289 | 387140 | + | -3.42 | 4.43E-10 | 0.02 | 8.02E-02 |
| ENSGALG00000011009 | SCN2A | 7 | 19912741 | 19968241 | - | -3.44 | 1.07E-05 | 0.00 | 0.00E+00 |
| ENSGALG00000035845 | JPH1 | 2 | 118543324 | 118621765 | - | -3.45 | 1.62E-16 | 0.06 | 6.59E-02 |
| ENSGALG00000016511 | ADGRG2 | 1 | 120395079 | 120415594 | + | -3.49 | 1.30E-07 | 0.12 | 0.00E+00 |
| ENSGALG00000003129 | ENSGALG00000003129 | 22 | 2247858 | 2251407 | - | -3.49 | 3.74E-13 | 0.14 | 7.57E-02 |
| ENSGALG00000009738 | ENSGALG00000009738 | 6 | 32280594 | 32288663 | - | -3.50 | 1.93E-21 | 0.38 | 6.68E-01 |
| ENSGALG00000011227 | CDS1 | 4 | 47124908 | 47149837 | + | -3.54 | 1.39E-13 | 0.12 | 1.57E-01 |
| ENSGALG00000000695 | MFSD4A | 26 | 2177659 | 2191981 | + | -3.56 | 8.50E-61 | 0.80 | 7.10E-01 |
| ENSGALG00000006076 | RASGEF1C | 13 | 14051308 | 14115158 | + | -3.62 | 2.54E-17 | 0.23 | 8.69E-02 |
| ENSGALG00000037851 | KK34 | 13 | 16816438 | 16818397 | + | -3.63 | 1.27E-07 | 0.00 | 7.51E-02 |
| ENSGALG00000034932 | BACE2 | 1 | 108844363 | 108894770 | + | -3.69 | 5.87E-39 | 0.74 | 7.34E-01 |
| ENSGALG00000017405 | NPR3 | Z | 10035886 | 10086792 | - | -3.72 | 4.34E-09 | 0.03 | 4.99E-02 |
| ENSGALG00000030802 | SHROOM2 | 1 | 125013467 | 125134159 | - | -3.73 | 9.36E-13 | 0.10 | 1.12E-01 |
| ENSGALG00000026768 | SLCO4C1 | Z | 50223340 | 50282675 | + | -3.77 | 1.26E-14 | 0.29 | 5.48E-02 |
| ENSGALG00000031122 | NTNG1 | 8 | 828149 | 965393 | + | -3.89 | 1.87E-07 | 0.00 | 0.00E+00 |
| ENSGALG00000034077 | KCNK5 | 3 | 29274506 | 29304143 | + | -3.89 | 8.03E-14 | 0.06 | 1.61E-01 |
| ENSGALG00000013627 | SLC7A2 | 4 | 63439358 | 63479725 | - | -3.92 | 1.35E-08 | 0.00 | 1.34E-02 |
| ENSGALG00000029020 | PLPP4 | 6 | 30550105 | 30594979 | + | -3.97 | 5.65E-14 | 0.00 | 6.58E-02 |
| ENSGALG00000016324 | GSTA4 | 3 | 88727100 | 88737893 | + | -3.98 | 6.17E-18 | 1.44 | 7.60E-01 |
| ENSGALG00000008554 | IL17REL | 1 | 20036081 | 20070713 | - | -3.98 | 1.56E-10 | 0.21 | 3.98E-02 |
| ENSGALG00000043393 | CADM2 | 1 | 93328062 | 93520050 | - | -4.02 | 6.63E-08 | 0.03 | 0.00E+00 |
| ENSGALG00000029921 | CSRP2 | 1 | 38237620 | 38245641 | - | -4.02 | 1.02E-31 | 3.99 | 2.17E+00 |
| ENSGALG00000011668 | IL8L1 | 4 | 51270481 | 51273558 | + | -4.02 | 4.71E-16 | 0.13 | 8.60E-02 |
| ENSGALG00000020151 | NMU | 4 | 65450209 | 65461844 | + | -4.07 | 2.95E-09 | 0.09 | 0.00E+00 |
| ENSGALG00000013085 | PTPRO | 1 | 62813644 | 62962492 | + | -4.17 | 7.00E-17 | 0.05 | 1.03E-02 |
| ENSGALG00000016251 | ENSGALG00000016251 | 1 | 112766328 | 112855804 | - | -4.25 | 4.70E-13 | 0.04 | 0.00E+00 |
| ENSGALG00000027986 | NIPAL1 | 4 | 66993587 | 66999005 | - | -4.26 | 4.00E-18 | 0.04 | 4.63E-02 |
| ENSGALG00000015164 | ENSGALG00000015164 | Z | 37274817 | 37323179 | + | -4.26 | 2.77E-12 | 0.00 | 1.88E-02 |
| ENSGALG00000008902 | GIPC2 | 8 | 18894852 | 18916774 | - | -4.69 | 1.36E-45 | 0.30 | 3.37E-01 |
| ENSGALG00000014971 | SLC2A9 | 4 | 78949520 | 79033938 | + | -4.75 | 5.75E-18 | 0.05 | 1.35E-01 |
| ENSGALG00000012018 | UGT8 | 4 | 56620972 | 56646024 | - | -4.84 | 8.63E-59 | 0.60 | 2.40E-01 |
| ENSGALG00000028659 | BLEC1 | 16 | 118833 | 121187 | - | -5.01 | 4.59E-18 | 0.00 | 0.00E+00 |
| ENSGALG00000003446 | PRLR | Z | 10611245 | 10678470 | - | -5.09 | 2.28E-28 | 0.03 | 5.59E-02 |
| ENSGALG00000015833 | ENSGALG00000015833 | 3 | 77241401 | 77263253 | - | -5.13 | 1.86E-16 | 0.05 | 0.00E+00 |
| ENSGALG00000014640 | ENSGALG00000014640 | 25 | 2321183 | 2323873 | + | -5.19 | 2.81E-48 | 0.54 | 4.48E-01 |
| ENSGALG00000006394 | ENSGALG00000006394 | 14 | 7373165 | 7375955 | + | -5.20 | 4.60E-15 | 0.00 | 0.00E+00 |
| ENSGALG00000006453 | TF | 9 | 4589480 | 4601249 | - | -5.39 | 1.50E-80 | 10.97 | 6.89E+00 |
| ENSGALG00000015348 | ALCAM | 1 | 86503359 | 86619306 | + | -5.51 | 1.64E-59 | 1.23 | 3.27E+00 |
| ENSGALG00000008599 | ENSGALG00000008599 | 5 | 24652093 | 24652908 | + | -5.87 | 5.33E-29 | 0.15 | 0.00E+00 |
| ENSGALG00000037511 | CNR1 | 3 | 76458678 | 76464108 | + | -5.91 | 7.77E-29 | 0.02 | 0.00E+00 |
| ENSGALG00000034860 | BCHE | 9 | 21061419 | 21088025 | + | -6.05 | 2.07E-26 | 0.00 | 2.60E-02 |
| ENSGALG00000032986 | CFTR | 1 | 24554513 | 24638605 | - | -6.12 | 1.40E-45 | 0.01 | 3.23E-02 |
| ENSGALG00000008552 | MAL | 3 | 16102820 | 16107198 | + | -7.20 | 3.55E-50 | 0.00 | 4.17E-02 |
| ENSGALG00000009963 | LYZ | 1 | 35425914 | 35429601 | + | -7.59 | 1.48E-59 | 1.27 | 9.20E-02 |
| ENSGALG00000011664 | RASSF6 | 4 | 51209468 | 51224086 | - | -7.92 | 2.05E-50 | 0.00 | 0.00E+00 |
| ENSGALG00000004320 | FAT2 | 13 | 12959375 | 12996291 | + | -8.04 | 2.70E-55 | 0.00 | 0.00E+00 |
| ENSGALG00000014759 | STYK1 | 1 | 78365923 | 78371775 | + | -8.08 | 1.27E-95 | 0.03 | 0.00E+00 |
| ENSGALG00000014913 | ROS1 | 3 | 63224022 | 63296367 | - | -8.44 | 3.08E-66 | 0.01 | 0.00E+00 |
| ENSGALG00000030834 | ATP8B1 | Z | 342538 | 365879 | + | -8.69 | 8.00E-80 | 0.01 | 0.00E+00 |
| ENSGALG00000011930 | OVST | 1 | 76345584 | 76430119 | - | -8.89 | 1.04E-105 | 0.06 | 3.65E-01 |
| ENSGALG00000010152 | TSPAN8 | 1 | 36146151 | 36163273 | - | -9.15 | 6.25E-112 | 0.04 | 0.00E+00 |

**S4 Table. A complete list of significantly enriched Gene Ontology biological processes for the down-regulated protein-coding genes**

| **GO term** | **GO ID** | **FDR** | **Gene number** |
| --- | --- | --- | --- |
| response to stimulus | GO:0050896 | 1.16E-14 | 123 |
| regulation of cellular process | GO:0050794 | 3.35E-14 | 145 |
| signaling | GO:0023052 | 9.21E-14 | 101 |
| cell communication | GO:0007154 | 1.68E-13 | 101 |
| signal transduction | GO:0007165 | 9.55E-12 | 92 |
| multicellular organismal process | GO:0032501 | 1.33E-11 | 98 |
| cellular response to stimulus | GO:0051716 | 6.24E-11 | 104 |
| developmental process | GO:0032502 | 6.30E-11 | 89 |
| anatomical structure development | GO:0048856 | 5.95E-10 | 83 |
| response to chemical | GO:0042221 | 6.08E-10 | 64 |
| cell differentiation | GO:0030154 | 1.61E-09 | 65 |
| localization | GO:0051179 | 2.33E-09 | 93 |
| phosphate-containing compound metabolic process | GO:0006796 | 2.71E-09 | 58 |
| locomotion | GO:0040011 | 2.84E-09 | 42 |
| phosphorus metabolic process | GO:0006793 | 3.55E-09 | 58 |
| multicellular organism development | GO:0007275 | 3.91E-09 | 76 |
| cellular developmental process | GO:0048869 | 4.73E-09 | 65 |
| system development | GO:0048731 | 1.07E-08 | 71 |
| regulation of cell communication | GO:0010646 | 1.66E-08 | 58 |
| regulation of signaling | GO:0023051 | 1.96E-08 | 58 |
| regulation of localization | GO:0032879 | 2.24E-08 | 50 |
| chemotaxis | GO:0006935 | 7.01E-08 | 22 |
| taxis | GO:0042330 | 8.38E-08 | 22 |
| intracellular signal transduction | GO:0035556 | 1.56E-07 | 50 |
| anatomical structure morphogenesis | GO:0009653 | 2.12E-07 | 50 |
| organic substance metabolic process | GO:0071704 | 2.25E-07 | 133 |
| movement of cell or subcellular component | GO:0006928 | 3.17E-07 | 42 |
| positive regulation of biological process | GO:0048518 | 3.41E-07 | 83 |
| negative regulation of cell communication | GO:0010648 | 4.04E-07 | 33 |
| negative regulation of signaling | GO:0023057 | 4.45E-07 | 33 |
| regulation of signal transduction | GO:0009966 | 5.44E-07 | 51 |
| metabolic process | GO:0008152 | 5.60E-07 | 136 |
| negative regulation of biological process | GO:0048519 | 6.31E-07 | 73 |
| response to external stimulus | GO:0009605 | 7.34E-07 | 43 |
| neuron projection guidance | GO:0097485 | 8.06E-07 | 14 |
| negative regulation of cellular process | GO:0048523 | 8.82E-07 | 69 |
| negative regulation of response to stimulus | GO:0048585 | 1.23E-06 | 35 |
| positive regulation of cellular process | GO:0048522 | 1.32E-06 | 77 |
| cell migration | GO:0016477 | 1.50E-06 | 33 |
| negative regulation of signal transduction | GO:0009968 | 1.56E-06 | 31 |
| cell morphogenesis involved in differentiation | GO:0000904 | 2.37E-06 | 23 |
| regulation of molecular function | GO:0065009 | 2.80E-06 | 46 |
| positive regulation of molecular function | GO:0044093 | 3.11E-06 | 34 |
| phosphorylation | GO:0016310 | 3.59E-06 | 40 |
| protein phosphorylation | GO:0006468 | 4.42E-06 | 37 |
| regulation of catalytic activity | GO:0050790 | 5.07E-06 | 38 |
| cellular metabolic process | GO:0044237 | 5.21E-06 | 124 |
| regulation of response to stimulus | GO:0048583 | 5.79E-06 | 58 |
| transmembrane receptor protein tyrosine kinase signaling pathway | GO:0007169 | 6.67E-06 | 21 |
| axon guidance | GO:0007411 | 7.00E-06 | 13 |
| primary metabolic process | GO:0044238 | 7.65E-06 | 123 |
| organonitrogen compound metabolic process | GO:1901564 | 8.43E-06 | 87 |
| plasma membrane bounded cell projection morphogenesis | GO:0120039 | 1.13E-05 | 20 |
| cell projection morphogenesis | GO:0048858 | 1.19E-05 | 20 |
| cell development | GO:0048468 | 1.48E-05 | 38 |
| localization of cell | GO:0051674 | 1.79E-05 | 33 |
| cell motility | GO:0048870 | 1.79E-05 | 33 |
| regulation of cell migration | GO:0030334 | 1.80E-05 | 24 |
| cell part morphogenesis | GO:0032990 | 2.38E-05 | 20 |
| cell surface receptor signaling pathway | GO:0007166 | 2.56E-05 | 45 |
| regulation of locomotion | GO:0040012 | 3.11E-05 | 25 |
| nervous system development | GO:0007399 | 3.53E-05 | 39 |
| cellular response to chemical stimulus | GO:0070887 | 4.67E-05 | 45 |
| positive regulation of macromolecule metabolic process | GO:0010604 | 4.84E-05 | 52 |
| regulation of cell motility | GO:2000145 | 5.09E-05 | 24 |
| cell morphogenesis | GO:0000902 | 5.49E-05 | 25 |
| positive regulation of metabolic process | GO:0009893 | 8.48E-05 | 54 |
| neuron development | GO:0048666 | 8.90E-05 | 25 |
| regulation of cellular component movement | GO:0051270 | 1.73E-04 | 24 |
| regulation of macromolecule metabolic process | GO:0060255 | 1.80E-04 | 75 |
| regulation of phosphorylation | GO:0042325 | 1.93E-04 | 27 |
| axon development | GO:0061564 | 2.01E-04 | 16 |
| neuron projection morphogenesis | GO:0048812 | 2.06E-04 | 18 |
| regulation of multicellular organismal process | GO:0051239 | 2.15E-04 | 41 |
| cellular component morphogenesis | GO:0032989 | 2.25E-04 | 20 |
| biological adhesion | GO:0022610 | 2.38E-04 | 28 |
| regulation of metabolic process | GO:0019222 | 2.56E-04 | 79 |
| enzyme linked receptor protein signaling pathway | GO:0007167 | 2.75E-04 | 24 |
| regulation of protein phosphorylation | GO:0001932 | 3.17E-04 | 25 |
| regulation of transferase activity | GO:0051338 | 3.29E-04 | 22 |
| cell-cell signaling | GO:0007267 | 3.42E-04 | 28 |
| neuron projection development | GO:0031175 | 3.52E-04 | 22 |
| regulation of intracellular signal transduction | GO:1902531 | 3.65E-04 | 31 |
| axonogenesis | GO:0007409 | 4.96E-04 | 15 |
| regulation of protein kinase activity | GO:0045859 | 5.23E-04 | 19 |
| immune system process | GO:0002376 | 6.08E-04 | 38 |
| positive regulation of cell migration | GO:0030335 | 6.69E-04 | 16 |
| regulation of kinase activity | GO:0043549 | 6.93E-04 | 20 |
| cell adhesion | GO:0007155 | 7.22E-04 | 27 |
| plasma membrane bounded cell projection organization | GO:0120036 | 9.41E-04 | 28 |
| establishment of localization | GO:0051234 | 1.05E-03 | 63 |
| positive regulation of cell motility | GO:2000147 | 1.09E-03 | 16 |
| regulation of phosphate metabolic process | GO:0019220 | 1.14E-03 | 28 |
| regulation of phosphorus metabolic process | GO:0051174 | 1.16E-03 | 28 |
| MAPK cascade | GO:0000165 | 1.17E-03 | 19 |
| animal organ development | GO:0048513 | 1.18E-03 | 47 |
| cell projection organization | GO:0030030 | 1.25E-03 | 28 |
| positive regulation of cellular component movement | GO:0051272 | 1.29E-03 | 16 |
| response to organic substance | GO:0010033 | 1.31E-03 | 40 |
| transport | GO:0006810 | 1.44E-03 | 61 |
| positive regulation of locomotion | GO:0040017 | 1.53E-03 | 16 |
| generation of neurons | GO:0048699 | 1.64E-03 | 28 |
| nitrogen compound metabolic process | GO:0006807 | 1.78E-03 | 110 |
| response to fluid shear stress | GO:0034405 | 1.85E-03 | 5 |
| cell morphogenesis involved in neuron differentiation | GO:0048667 | 1.89E-03 | 16 |
| regulation of biological quality | GO:0065008 | 1.98E-03 | 50 |
| cell death | GO:0008219 | 2.11E-03 | 31 |
| lipid metabolic process | GO:0006629 | 2.34E-03 | 26 |
| positive regulation of catalytic activity | GO:0043085 | 2.57E-03 | 24 |
| neuron differentiation | GO:0030182 | 2.70E-03 | 26 |
| small molecule metabolic process | GO:0044281 | 2.80E-03 | 30 |
| regulation of cellular metabolic process | GO:0031323 | 3.25E-03 | 70 |
| positive regulation of nitrogen compound metabolic process | GO:0051173 | 3.34E-03 | 44 |
| regulation of developmental process | GO:0050793 | 3.37E-03 | 37 |
| cellular response to organic substance | GO:0071310 | 3.44E-03 | 35 |
| tube development | GO:0035295 | 3.59E-03 | 23 |
| negative regulation of developmental process | GO:0051093 | 4.30E-03 | 20 |
| negative regulation of phosphorylation | GO:0042326 | 4.72E-03 | 13 |
| negative regulation of transferase activity | GO:0051348 | 4.81E-03 | 11 |
| response to stress | GO:0006950 | 5.06E-03 | 46 |
| regulation of body fluid levels | GO:0050878 | 5.11E-03 | 11 |
| negative regulation of catalytic activity | GO:0043086 | 5.42E-03 | 16 |
| regulation of protein modification process | GO:0031399 | 5.44E-03 | 28 |
| tube morphogenesis | GO:0035239 | 5.59E-03 | 20 |
| regulation of MAPK cascade | GO:0043408 | 6.14E-03 | 17 |
| positive regulation of cellular biosynthetic process | GO:0031328 | 6.30E-03 | 32 |
| negative regulation of cellular response to growth factor stimulus | GO:0090288 | 6.37E-03 | 8 |
| negative regulation of protein phosphorylation | GO:0001933 | 7.06E-03 | 12 |
| neurogenesis | GO:0022008 | 7.40E-03 | 28 |
| positive regulation of biosynthetic process | GO:0009891 | 8.55E-03 | 32 |
| positive regulation of cell communication | GO:0010647 | 8.69E-03 | 28 |
| negative regulation of kinase activity | GO:0033673 | 9.68E-03 | 10 |
| epithelium development | GO:0060429 | 9.94E-03 | 23 |
| positive regulation of signaling | GO:0023056 | 9.98E-03 | 28 |
| response to laminar fluid shear stress | GO:0034616 | 1.00E-02 | 3 |
| positive regulation of cytokine production | GO:0001819 | 1.05E-02 | 12 |
| organic acid metabolic process | GO:0006082 | 1.17E-02 | 19 |
| protein modification process | GO:0036211 | 1.32E-02 | 51 |
| cellular protein modification process | GO:0006464 | 1.32E-02 | 51 |
| positive regulation of cellular metabolic process | GO:0031325 | 1.34E-02 | 44 |
| regulation of primary metabolic process | GO:0080090 | 1.35E-02 | 66 |
| defense response | GO:0006952 | 1.35E-02 | 23 |
| negative regulation of phosphate metabolic process | GO:0045936 | 1.42E-02 | 14 |
| negative regulation of phosphorus metabolic process | GO:0010563 | 1.42E-02 | 14 |
| blood vessel development | GO:0001568 | 1.53E-02 | 17 |
| apoptotic process | GO:0006915 | 1.55E-02 | 27 |
| regulation of nitrogen compound metabolic process | GO:0051171 | 2.12E-02 | 64 |
| regulation of protein metabolic process | GO:0051246 | 2.20E-02 | 36 |
| positive regulation of macromolecule biosynthetic process | GO:0010557 | 2.23E-02 | 30 |
| carboxylic acid metabolic process | GO:0019752 | 2.50E-02 | 18 |
| regulation of transport | GO:0051049 | 2.61E-02 | 26 |
| macromolecule modification | GO:0043412 | 2.66E-02 | 52 |
| oxoacid metabolic process | GO:0043436 | 2.71E-02 | 18 |
| programmed cell death | GO:0012501 | 2.76E-02 | 27 |
| tissue development | GO:0009888 | 2.84E-02 | 30 |
| negative regulation of protein kinase activity | GO:0006469 | 3.02E-02 | 9 |
| vasculature development | GO:0001944 | 3.13E-02 | 17 |
| regulation of response to external stimulus | GO:0032101 | 3.22E-02 | 17 |
| animal organ morphogenesis | GO:0009887 | 3.33E-02 | 21 |
| negative regulation of molecular function | GO:0044092 | 3.33E-02 | 19 |
| immune response | GO:0006955 | 3.61E-02 | 24 |
| biosynthetic process | GO:0009058 | 3.69E-02 | 68 |
| cell-cell signaling by wnt | GO:0198738 | 3.70E-02 | 13 |
| positive regulation of transcription by RNA polymerase II | GO:0045944 | 3.71E-02 | 23 |
| regulation of cellular response to growth factor stimulus | GO:0090287 | 4.38E-02 | 11 |
| positive regulation of multicellular organismal process | GO:0051240 | 4.38E-02 | 23 |
| organic substance biosynthetic process | GO:1901576 | 4.39E-02 | 67 |
| canonical Wnt signaling pathway | GO:0060070 | 4.58E-02 | 11 |
| regulation of establishment of cell polarity | GO:2000114 | 4.76E-02 | 4 |
| cellular modified amino acid metabolic process | GO:0006575 | 4.89E-02 | 8 |
| negative regulation of apoptotic process | GO:0043066 | 4.90E-02 | 17 |

Note: The top 10 most significant GO terms were shown in Figure 3E.

**S5 Table. A list of conserved lncRNAs between chicken and human**

| **Transcript ID** | **Gene ID** | **Human ortholog** |
| --- | --- | --- |
| *ENSGALT00000068702* | *ENSGALG00000006584* | *lnc-BLID-5:1* |
| *ENSGALT00000070647* | *ENSGALG00000006584* | *lnc-BLID-5:1* |
| *ENSGALT00000045558* | *ENSGALG00000028647* | *lnc-FAM159A-1:1* |
| *ENSGALT00000063955* | *ENSGALG00000028647* | *lnc-FAM159A-1:1* |
| *ENSGALT00000049219* | *ENSGALG00000028647* | *lnc-FAM159A-1:1* |
| *ENSGALT00000049739* | *ENSGALG00000028647* | *lnc-FAM159A-1:1* |
| *ENSGALT00000046951* | *ENSGALG00000029187* | *lnc-ZNF80-4:7* |
| *ENSGALT00000079620* | *ENSGALG00000029187* | *lnc-ZNF80-4:7* |
| *ENSGALT00000049096* | *ENSGALG00000029460* | *FOXG1-AS1:5* |
| *ENSGALT00000056492* | *ENSGALG00000029460* | *FOXG1-AS1:5* |
| *ENSGALT00000065451* | *ENSGALG00000029733* | *lnc-TLK2-1:1* |
| *ENSGALT00000066196* | *ENSGALG00000029733* | *lnc-TLK2-1:1* |
| *ENSGALT00000053770* | *ENSGALG00000029954* | *CASK-AS1:1* |
| *ENSGALT00000070587* | *ENSGALG00000029954* | *CASK-AS1:1* |
| *ENSGALT00000050772* | *ENSGALG00000030014* | *lnc-RP11-44L9.1.1-6:1* |
| *ENSGALT00000063663* | *ENSGALG00000030014* | *lnc-RP11-44L9.1.1-6:1* |
| *ENSGALT00000067101* | *ENSGALG00000030014* | *lnc-RP11-44L9.1.1-6:1* |
| *ENSGALT00000069636* | *ENSGALG00000030014* | *lnc-RP11-44L9.1.1-6:1* |
| *ENSGALT00000047200* | *ENSGALG00000030257* | *lnc-GPD2-2:3* |
| *ENSGALT00000071215* | *ENSGALG00000030257* | *lnc-GPD2-2:3* |
| *ENSGALT00000058913* | *ENSGALG00000030257* | *lnc-GPD2-2:3* |
| *ENSGALT00000074003* | *ENSGALG00000030257* | *lnc-GPD2-2:3* |
| *ENSGALT00000053517* | *ENSGALG00000030257* | *lnc-GPD2-2:3* |
| *ENSGALT00000070559* | *ENSGALG00000030257* | *lnc-GPD2-2:3* |
| *ENSGALT00000050110* | *ENSGALG00000030422* | *TUNAR:11* |
| *ENSGALT00000064215* | *ENSGALG00000030422* | *TUNAR:11* |
| *ENSGALT00000072177* | *ENSGALG00000030422* | *TUNAR:11* |
| *ENSGALT00000082597* | *ENSGALG00000030422* | *TUNAR:11* |
| *ENSGALT00000065536* | *ENSGALG00000031007* | *lnc-PPAPDC1B-1:1* |
| *ENSGALT00000071794* | *ENSGALG00000031007* | *lnc-PPAPDC1B-1:1* |
| *ENSGALT00000051906* | *ENSGALG00000031040* | *TEX41:59* |
| *ENSGALT00000054503* | *ENSGALG00000031040* | *TEX41:59* |
| *ENSGALT00000049509* | *ENSGALG00000032114* | *DNM3OS:3* |
| *ENSGALT00000083071* | *ENSGALG00000032114* | *DNM3OS:3* |
| *ENSGALT00000047660* | *ENSGALG00000032646* | *lnc-LRRC52-6:1* |
| *ENSGALT00000067126* | *ENSGALG00000032646* | *lnc-LRRC52-6:1* |
| *ENSGALT00000054291* | *ENSGALG00000032696* | *LHX5-AS1:3* |
| *ENSGALT00000064938* | *ENSGALG00000032696* | *LHX5-AS1:3* |
| *ENSGALT00000056824* | *ENSGALG00000032911* | *lnc-MAF-7:2* |
| *ENSGALT00000065198* | *ENSGALG00000032911* | *lnc-MAF-7:2* |
| *ENSGALT00000049316* | *ENSGALG00000032941* | *LINC00599:17* |
| *ENSGALT00000058872* | *ENSGALG00000032941* | *LINC00599:17* |
| *ENSGALT00000061495* | *ENSGALG00000033281* | *lnc-ROGDI-1:1* |
| *ENSGALT00000070039* | *ENSGALG00000033281* | *lnc-ROGDI-1:1* |
| *ENSGALT00000063274* | *ENSGALG00000033306* | *lnc-RP11-552I14.1.1-1:11* |
| *ENSGALT00000067740* | *ENSGALG00000033306* | *lnc-RP11-552I14.1.1-1:11* |
| *ENSGALT00000060509* | *ENSGALG00000033377* | *lnc-RHBDL3-4:2* |
| *ENSGALT00000080729* | *ENSGALG00000033377* | *lnc-RHBDL3-4:2* |
| *ENSGALT00000052555* | *ENSGALG00000033448* | *lnc-GFOD1-2:1* |
| *ENSGALT00000062933* | *ENSGALG00000033448* | *lnc-GFOD1-2:1* |
| *ENSGALT00000050608* | *ENSGALG00000035027* | *lnc-MRPS9-4:1* |
| *ENSGALT00000058491* | *ENSGALG00000035027* | *lnc-MRPS9-4:1* |
| *ENSGALT00000052276* | *ENSGALG00000035027* | *lnc-MRPS9-4:1* |
| *ENSGALT00000076518* | *ENSGALG00000035027* | *lnc-MRPS9-4:1* |
| *ENSGALT00000072804* | *ENSGALG00000035197* | *lnc-AC016251.1-7:2* |
| *ENSGALT00000075644* | *ENSGALG00000035197* | *lnc-AC016251.1-7:2* |
| *ENSGALT00000050406* | *ENSGALG00000035324* | *lnc-CELF2-4:1* |
| *ENSGALT00000069906* | *ENSGALG00000035324* | *lnc-CELF2-4:1* |
| *ENSGALT00000058303* | *ENSGALG00000035371* | *lnc-HECA-1:3* |
| *ENSGALT00000065844* | *ENSGALG00000035371* | *lnc-HECA-1:3* |
| *ENSGALT00000073788* | *ENSGALG00000035778* | *lnc-ERC1-3:5* |
| *ENSGALT00000079244* | *ENSGALG00000035778* | *lnc-ERC1-3:5* |
| *ENSGALT00000069153* | *ENSGALG00000036388* | *lnc-RP3-486B10.1.1-4:15* |
| *ENSGALT00000071119* | *ENSGALG00000036388* | *lnc-RP3-486B10.1.1-4:15* |
| *ENSGALT00000047571* | *ENSGALG00000036388* | *lnc-RP3-486B10.1.1-4:15* |
| *ENSGALT00000055558* | *ENSGALG00000036388* | *lnc-RP3-486B10.1.1-4:15* |
| *ENSGALT00000060880* | *ENSGALG00000036548* | *lnc-OTUD7A-1:1* |
| *ENSGALT00000061189* | *ENSGALG00000036548* | *lnc-OTUD7A-1:1* |
| *ENSGALT00000060502* | *ENSGALG00000036563* | *lnc-LYSMD4-1:2* |
| *ENSGALT00000062057* | *ENSGALG00000036563* | *lnc-LYSMD4-1:2* |
| *ENSGALT00000056974* | *ENSGALG00000036773* | *LINC00581:6* |
| *ENSGALT00000058652* | *ENSGALG00000036773* | *LINC00581:6* |
| *ENSGALT00000048402* | *ENSGALG00000036773* | *LINC00581:6* |
| *ENSGALT00000081415* | *ENSGALG00000036773* | *LINC00581:6* |
| *ENSGALT00000053177* | *ENSGALG00000037049* | *lnc-HELT-2:3* |
| *ENSGALT00000077009* | *ENSGALG00000037049* | *lnc-HELT-2:3* |
| *ENSGALT00000069865* | *ENSGALG00000037574* | *lnc-RC3H1-2:1* |
| *ENSGALT00000070550* | *ENSGALG00000037574* | *lnc-RC3H1-2:1* |
| *ENSGALT00000056471* | *ENSGALG00000037847* | *lnc-NRIP1-2:2* |
| *ENSGALT00000061820* | *ENSGALG00000037847* | *lnc-NRIP1-2:2* |
| *ENSGALT00000046522* | *ENSGALG00000037847* | *lnc-NRIP1-2:10* |
| *ENSGALT00000082243* | *ENSGALG00000037847* | *lnc-NRIP1-2:10* |
| *ENSGALT00000047298* | *ENSGALG00000038169* | *lnc-TSPY10-14:1* |
| *ENSGALT00000047856* | *ENSGALG00000038169* | *lnc-TSPY10-14:1* |
| *ENSGALT00000079592* | *ENSGALG00000038293* | *PRKG1-AS1:4* |
| *ENSGALT00000081081* | *ENSGALG00000038293* | *PRKG1-AS1:4* |
| *ENSGALT00000064142* | *ENSGALG00000038982* | *lnc-HMBOX1-1:3* |
| *ENSGALT00000066618* | *ENSGALG00000038982* | *lnc-HMBOX1-1:3* |
| *ENSGALT00000052538* | *ENSGALG00000039074* | *LINC00682:8* |
| *ENSGALT00000066500* | *ENSGALG00000039074* | *LINC00682:8* |
| *ENSGALT00000063939* | *ENSGALG00000039074* | *LINC00682:8* |
| *ENSGALT00000075050* | *ENSGALG00000039074* | *LINC00682:8* |
| *ENSGALT00000062784* | *ENSGALG00000039267* | *lnc-C12orf45-3:1* |
| *ENSGALT00000065690* | *ENSGALG00000039267* | *lnc-C12orf45-3:1* |
| *ENSGALT00000067557* | *ENSGALG00000039449* | *lnc-LRP12-1:1* |
| *ENSGALT00000079958* | *ENSGALG00000039449* | *lnc-LRP12-1:1* |
| *ENSGALT00000064823* | *ENSGALG00000039962* | *lnc-CCDC89-5:7* |
| *ENSGALT00000079084* | *ENSGALG00000039962* | *lnc-CCDC89-5:7* |
| *ENSGALT00000058239* | *ENSGALG00000040043* | *lnc-FAM212B-3:1* |
| *ENSGALT00000064290* | *ENSGALG00000040043* | *lnc-FAM212B-3:1* |
| *ENSGALT00000067198* | *ENSGALG00000040385* | *lnc-ZFHX4-2:8* |
| *ENSGALT00000082420* | *ENSGALG00000040385* | *lnc-ZFHX4-2:8* |
| *ENSGALT00000049099* | *ENSGALG00000040631* | *UGDH-AS1:10* |
| *ENSGALT00000050207* | *ENSGALG00000040631* | *UGDH-AS1:10* |
| *ENSGALT00000047284* | *ENSGALG00000040767* | *lnc-KIAA1737-1:5* |
| *ENSGALT00000059498* | *ENSGALG00000040767* | *lnc-KIAA1737-1:5* |
| *ENSGALT00000062728* | *ENSGALG00000041015* | *lnc-SIX2-3:12* |
| *ENSGALT00000073995* | *ENSGALG00000041015* | *lnc-SIX2-3:12* |
| *ENSGALT00000048198* | *ENSGALG00000041053* | *lnc-KIDINS220-4:14* |
| *ENSGALT00000069674* | *ENSGALG00000041053* | *lnc-KIDINS220-4:14* |
| *ENSGALT00000063301* | *ENSGALG00000041291* | *lnc-HSD17B12-1:12* |
| *ENSGALT00000073073* | *ENSGALG00000041291* | *lnc-HSD17B12-1:12* |
| *ENSGALT00000065756* | *ENSGALG00000041291* | *lnc-HSD17B12-1:12* |
| *ENSGALT00000068680* | *ENSGALG00000041291* | *lnc-HSD17B12-1:12* |
| *ENSGALT00000064593* | *ENSGALG00000041615* | *lnc-BRAF-1:2* |
| *ENSGALT00000079855* | *ENSGALG00000041615* | *lnc-BRAF-1:2* |
| *ENSGALT00000048812* | *ENSGALG00000041681* | *lnc-C15orf41-18:11* |
| *ENSGALT00000063720* | *ENSGALG00000041681* | *lnc-C15orf41-18:11* |
| *ENSGALT00000055545* | *ENSGALG00000041770* | *FEZF1-AS1:2* |
| *ENSGALT00000059191* | *ENSGALG00000041770* | *FEZF1-AS1:2* |
| *ENSGALT00000057253* | *ENSGALG00000041805* | *lnc-TNR-4:1* |
| *ENSGALT00000063765* | *ENSGALG00000041805* | *lnc-TNR-4:1* |
| *ENSGALT00000056915* | *ENSGALG00000041987* | *lnc-SLC25A21-4:1* |
| *ENSGALT00000070996* | *ENSGALG00000041987* | *lnc-SLC25A21-4:1* |
| *ENSGALT00000064506* | *ENSGALG00000042484* | *LINC01102:2* |
| *ENSGALT00000081282* | *ENSGALG00000042484* | *LINC01102:2* |
| *ENSGALT00000046271* | *ENSGALG00000042517* | *MAGI2-AS3:59* |
| *ENSGALT00000055683* | *ENSGALG00000042517* | *MAGI2-AS3:59* |
| *ENSGALT00000061293* | *ENSGALG00000042805* | *lnc-GFOD1-2:1* |
| *ENSGALT00000072323* | *ENSGALG00000042805* | *lnc-GFOD1-2:1* |
| *ENSGALT00000064039* | *ENSGALG00000042953* | *LINC01572:18* |
| *ENSGALT00000065022* | *ENSGALG00000042953* | *LINC01572:18* |
| *ENSGALT00000059102* | *ENSGALG00000042953* | *LINC01572:18* |
| *ENSGALT00000077023* | *ENSGALG00000042953* | *LINC01572:18* |
| *TCONS_00000430* | *XLOC_000135* | *LINC01392:2* |
| *TCONS_00000431* | *XLOC_000135* | *LINC01392:2* |
| *TCONS_00002345* | *XLOC_000701* | *lnc-UTY-1:52* |
| *TCONS_00002524* | *XLOC_000746* | *lnc-RAI2-5:1* |
| *TCONS_00004797* | *XLOC_001407* | *lnc-BICD1-1:1* |
| *TCONS_00005501* | *XLOC_001612* | *lnc-RCSD1-3:1* |
| *TCONS_00005502* | *XLOC_001612* | *lnc-RCSD1-3:1* |
| *TCONS_00006739* | *XLOC_001963* | *lnc-CT62-3:1* |
| *TCONS_00010803* | *XLOC_004502* | *CRNDE:39* |
| *TCONS_00010804* | *XLOC_004502* | *CRNDE:39* |
| *TCONS_00010805* | *XLOC_004502* | *CRNDE:39* |
| *TCONS_00010806* | *XLOC_004502* | *CRNDE:39* |
| *TCONS_00010807* | *XLOC_004502* | *CRNDE:39* |
| *TCONS_00010808* | *XLOC_004502* | *CRNDE:39* |
| *TCONS_00010809* | *XLOC_004502* | *CRNDE:39* |
| *TCONS_00010810* | *XLOC_004502* | *CRNDE:39* |
| *TCONS_00010811* | *XLOC_004502* | *CRNDE:39* |
| *TCONS_00013427* | *XLOC_005549* | *MIR3142HG:1* |
| *TCONS_00014225* | *XLOC_005787* | *lnc-HNRNPA0-1:1* |
| *TCONS_00015468* | *XLOC_006218* | *lnc-SLC10A6-1:1* |
| *TCONS_00015644* | *XLOC_006274* | *lnc-XYLT1-2:1* |
| *TCONS_00015646* | *XLOC_006274* | *lnc-XYLT1-2:1* |
| *TCONS_00015647* | *XLOC_006274* | *lnc-XYLT1-2:1* |
| *TCONS_00016943* | *XLOC_006714* | *lnc-IFT81-5:1* |
| *TCONS_00018927* | *XLOC_007449* | *lnc-TMC8-1:1* |
| *TCONS_00022010* | *XLOC_008419* | *lnc-HOXA9-1:2* |
| *TCONS_00022310* | *XLOC_008502* | *lnc-HERPUD2-1:2* |
| *TCONS_00022484* | *XLOC_008552* | *CASC15:19* |
| *TCONS_00022485* | *XLOC_008553* | *lnc-SOX4-2:1* |
| *TCONS_00023605* | *XLOC_008869* | *CASC7:1* |
| *TCONS_00023606* | *XLOC_008869* | *CASC7:1* |
| *TCONS_00023607* | *XLOC_008869* | *CASC7:1* |
| *TCONS_00024563* | *XLOC_009145* | *lnc-ADCYAP1R1-1:1* |
| *TCONS_00024734* | *XLOC_009192* | *CASC15:19* |
| *TCONS_00025521* | *XLOC_009402* | *lnc-XKR4-3:1* |
| *TCONS_00027400* | *XLOC_010841* | *lnc-LPIN3-1:1* |
| *TCONS_00027401* | *XLOC_010841* | *lnc-LPIN3-1:1* |
| *TCONS_00027940* | *XLOC_010991* | *lnc-PTPN1-3:1* |
| *TCONS_00027941* | *XLOC_010991* | *lnc-PTPN1-3:1* |
| *TCONS_00032271* | *XLOC_012561* | *lnc-C1orf132-1:14* |
| *TCONS_00032272* | *XLOC_012561* | *lnc-C1orf132-1:14* |
| *TCONS_00032273* | *XLOC_012561* | *lnc-C1orf132-1:14* |
| *TCONS_00032274* | *XLOC_012561* | *lnc-C1orf132-1:14* |
| *TCONS_00032275* | *XLOC_012561* | *lnc-C1orf132-1:14* |
| *TCONS_00032276* | *XLOC_012561* | *lnc-C1orf132-1:14* |
| *TCONS_00032277* | *XLOC_012561* | *lnc-C1orf132-1:14* |
| *TCONS_00032278* | *XLOC_012561* | *lnc-C1orf132-1:14* |
| *TCONS_00033326* | *XLOC_012961* | *lnc-GPX6-3:1* |
| *TCONS_00037028* | *XLOC_014167* | *lnc-MEIS1-3:8* |
| *TCONS_00037029* | *XLOC_014167* | *lnc-MEIS1-3:8* |
| *TCONS_00037030* | *XLOC_014167* | *lnc-MEIS1-3:8* |
| *TCONS_00037031* | *XLOC_014167* | *lnc-MEIS1-3:8* |
| *TCONS_00037132* | *XLOC_014196* | *lnc-TMEM178-6:2* |
| *TCONS_00037133* | *XLOC_014196* | *lnc-TMEM178-6:2* |
| *TCONS_00040042* | *XLOC_015827* | *lnc-IL31RA-2:1* |
| *TCONS_00040494* | *XLOC_015996* | *lnc-RAP2C-2:1* |
| *TCONS_00041856* | *XLOC_016349* | *lnc-DCAF4L1-2:1* |
| *TCONS_00043689* | *XLOC_016829* | *lnc-PDLIM3-2:1* |
| *TCONS_00043690* | *XLOC_016829* | *lnc-PDLIM3-2:1* |
| *TCONS_00043691* | *XLOC_016829* | *lnc-PDLIM3-2:1* |
| *TCONS_00045478* | *XLOC_017890* | *lnc-SLC22A18-2:1* |
| *TCONS_00047245* | *XLOC_018381* | *lnc-RAPSN-2:1* |
| *TCONS_00047247* | *XLOC_018381* | *lnc-RAPSN-2:1* |
| *TCONS_00048747* | *XLOC_019173* | *lnc-WAPAL-2:1* |
| *TCONS_00048748* | *XLOC_019173* | *lnc-WAPAL-2:1* |
| *TCONS_00049629* | *XLOC_019409* | *lnc-MGMT-12:1* |
| *TCONS_00049630* | *XLOC_019409* | *lnc-MGMT-12:1* |
| *TCONS_00049632* | *XLOC_019409* | *lnc-MGMT-12:1* |
| *TCONS_00049889* | *XLOC_019483* | *lnc-TET1-1:2* |
| *TCONS_00049987* | *XLOC_019515* | *lnc-ZNF503-1:4* |
| *TCONS_00049988* | *XLOC_019515* | *lnc-ZNF503-1:4* |
| *TCONS_00049989* | *XLOC_019515* | *lnc-ZNF503-1:4* |
| *TCONS_00049990* | *XLOC_019515* | *lnc-ZNF503-1:4* |
| *TCONS_00049991* | *XLOC_019515* | *lnc-ZNF503-1:4* |
| *TCONS_00049992* | *XLOC_019515* | *lnc-ZNF503-1:4* |
| *TCONS_00049993* | *XLOC_019515* | *lnc-ZNF503-1:4* |
| *TCONS_00049994* | *XLOC_019515* | *lnc-ZNF503-1:4* |
| *TCONS_00049995* | *XLOC_019515* | *lnc-ZNF503-1:4* |
| *TCONS_00049996* | *XLOC_019515* | *lnc-ZNF503-1:4* |
| *TCONS_00051360* | *XLOC_020124* | *lnc-DLX2-4:12* |
| *TCONS_00051361* | *XLOC_020124* | *lnc-DLX2-4:12* |
| *TCONS_00052656* | *XLOC_020477* | *lnc-MGAT5-3:1* |
| *TCONS_00052657* | *XLOC_020477* | *lnc-MGAT5-3:1* |
| *TCONS_00052658* | *XLOC_020477* | *lnc-MGAT5-3:1* |
| *TCONS_00053242* | *XLOC_020875* | *lnc-TEX22-2:2* |
| *TCONS_00053801* | *XLOC_021029* | *lnc-CPT2-3:8* |
| *TCONS_00057644* | *XLOC_022595* | *lnc-ENC1-5:1* |

**S6 Table. Common significantly up- and down-regulated known and novel lncRNAs in tumor samples as compared to both bursa and B cell controls**

| **ID** | **Name** | **Chr** | **Start** | **End** | **Strand** | **Tumor *vs* Bursa** | | **Tumor *vs* B cell** | |
| --- | --- | --- | --- | --- | --- | --- | --- | --- | --- |
|  |  |  |  |  |  | **Log_2_FC** | **FDR** | **Log_2_FC** | **FDR** |
| ENSGALG00000042870 | ENSGALG00000042870 | 1 | 32603425 | 32604254 | - | 6.58 | 9.92E-31 | 8.82 | 9.57E-19 |
| ENSGALG00000042004 | ENSGALG00000042004 | 1 | 108684582 | 108688789 | + | 3.11 | 2.51E-19 | 6.07 | 4.19E-28 |
| ENSGALG00000042098 | ENSGALG00000042098 | 2 | 47835978 | 47837609 | - | 3.09 | 2.94E-08 | 6.11 | 3.44E-10 |
| ENSGALG00000042216 | ENSGALG00000042216 | 1 | 145564574 | 145566571 | - | 2.71 | 1.41E-04 | 2.89 | 4.17E-04 |
| ENSGALG00000034279 | ENSGALG00000034279 | 1 | 112521130 | 112523110 | + | 2.52 | 2.21E-09 | 3.84 | 3.14E-13 |
| ENSGALG00000042116 | ENSGALG00000042116 | 3 | 73147318 | 73160230 | - | 1.67 | 8.32E-08 | 5.80 | 2.74E-42 |
| ENSGALG00000043226 | ENSGALG00000043226 | 12 | 10341365 | 10361566 | - | 1.62 | 2.67E-04 | 2.41 | 1.32E-06 |
| XLOC_000160 | XLOC_000160 | 1 | 32603301 | 32610772 | + | 13.75 | 4.06E-223 | 6.98 | 1.56E-10 |
| XLOC_023049 | XLOC_023049 | Z | 46907306 | 47022617 | - | 5.49 | 1.29E-29 | 8.62 | 2.87E-37 |
| XLOC_007966 | XLOC_007966 | 19 | 2165835 | 2251220 | - | 5.01 | 1.81E-36 | 8.16 | 1.95E-43 |
| XLOC_000733 | XLOC_000733 | 1 | 118409669 | 118420776 | + | 3.82 | 7.10E-09 | 3.54 | 1.58E-05 |
| XLOC_013839 | XLOC_013839 | 3 | 57154744 | 57185172 | + | 3.66 | 8.96E-10 | 7.32 | 3.18E-17 |
| XLOC_010886 | XLOC_010886 | 20 | 5558828 | 5592453 | + | 3.63 | 7.64E-10 | 3.47 | 2.36E-07 |
| XLOC_000176 | XLOC_000176 | 1 | 35044037 | 35048805 | + | 3.38 | 5.13E-06 | 4.82 | 2.80E-06 |
| XLOC_014004 | XLOC_014004 | 3 | 97233339 | 97261797 | + | 3.16 | 9.38E-06 | 5.89 | 9.82E-10 |
| XLOC_019378 | XLOC_019378 | 6 | 28130595 | 28152385 | + | 3.13 | 1.19E-12 | 3.02 | 9.86E-12 |
| XLOC_021993 | XLOC_021993 | 9 | 17500335 | 17508534 | - | 2.96 | 1.85E-04 | 3.78 | 5.25E-04 |
| XLOC_000630 | XLOC_000630 | 1 | 99431405 | 99470823 | + | 2.71 | 7.84E-04 | 6.08 | 6.38E-07 |
| XLOC_016105 | XLOC_016105 | 4 | 21565252 | 21568661 | + | 2.70 | 4.23E-04 | 4.24 | 1.51E-04 |
| XLOC_004542 | XLOC_004542 | 11 | 10238378 | 10414346 | + | 2.67 | 9.04E-08 | 2.77 | 2.00E-06 |
| XLOC_001457 | XLOC_001457 | 1 | 69787582 | 69798682 | - | 2.45 | 2.76E-07 | 2.58 | 4.99E-07 |
| XLOC_008472 | XLOC_008472 | 2 | 42864030 | 42885563 | + | 2.44 | 1.05E-04 | 5.39 | 2.60E-10 |
| XLOC_019219 | XLOC_019219 | 6 | 9404439 | 9406651 | + | 2.31 | 6.86E-04 | 3.85 | 9.76E-06 |
| XLOC_001598 | XLOC_001598 | 1 | 87639003 | 87648180 | - | 2.28 | 7.71E-06 | 3.07 | 9.08E-08 |
| XLOC_012207 | XLOC_012207 | 24 | 6133791 | 6135883 | - | 2.18 | 1.14E-08 | 4.00 | 6.61E-25 |
| XLOC_001407 | XLOC_001407 | 1 | 59138431 | 59163936 | - | 1.90 | 3.05E-06 | 3.75 | 4.29E-17 |
| XLOC_013176 | XLOC_013176 | 28 | 1630125 | 1636400 | + | 1.79 | 3.14E-05 | 6.82 | 2.36E-16 |
| XLOC_001597 | XLOC_001597 | 1 | 87633676 | 87638894 | - | 1.75 | 8.24E-04 | 2.12 | 4.71E-05 |
| XLOC_007965 | XLOC_007965 | 19 | 2081594 | 2131444 | - | 1.58 | 4.17E-04 | 7.92 | 1.01E-33 |
| ENSGALG00000040767 | ENSGALG00000040767 | 5 | 39059424 | 39060649 | - | -1.27 | 8.69E-05 | -1.22 | 7.80E-04 |
| ENSGALG00000043343 | ENSGALG00000043343 | 1 | 6607133 | 6607996 | + | -1.72 | 6.75E-05 | -4.02 | 2.84E-20 |
| ENSGALG00000039412 | ENSGALG00000039412 | 1 | 64560918 | 64571917 | + | -1.89 | 1.23E-13 | -2.55 | 5.00E-15 |
| ENSGALG00000043746 | ENSGALG00000043746 | 1 | 44880217 | 44884298 | - | -1.90 | 3.26E-05 | -3.03 | 4.74E-09 |
| ENSGALG00000042379 | ENSGALG00000042379 | 26 | 4972639 | 4973649 | + | -1.96 | 8.90E-04 | -4.77 | 5.02E-27 |
| ENSGALG00000030439 | ENSGALG00000030439 | 12 | 5255305 | 5257400 | - | -1.98 | 1.19E-14 | -1.44 | 2.03E-04 |
| ENSGALG00000031600 | ENSGALG00000031600 | 1 | 92057881 | 92059559 | + | -2.01 | 2.28E-09 | -1.93 | 7.57E-05 |
| ENSGALG00000038851 | ENSGALG00000038851 | 14 | 6336705 | 6337816 | - | -2.05 | 4.92E-07 | -1.64 | 7.56E-04 |
| ENSGALG00000033595 | ENSGALG00000033595 | 9 | 19636902 | 19640222 | + | -2.22 | 7.51E-23 | -2.22 | 3.13E-13 |
| ENSGALG00000021655 | ENSGALG00000021655 | 26 | 3760197 | 3762945 | - | -2.58 | 1.73E-11 | -2.61 | 7.75E-06 |
| ENSGALG00000035088 | ENSGALG00000035088 | 1 | 33085170 | 33086577 | - | -2.63 | 1.15E-08 | -1.84 | 6.58E-04 |
| ENSGALG00000029916 | ENSGALG00000029916 | 2 | 14507385 | 14509065 | - | -2.77 | 4.30E-05 | -5.36 | 1.02E-11 |
| ENSGALG00000036548 | ENSGALG00000036548 | 10 | 5491116 | 5492084 | + | -2.79 | 1.88E-05 | -3.61 | 4.71E-07 |
| ENSGALG00000034684 | ENSGALG00000034684 | 3 | 33116938 | 33125450 | + | -3.13 | 6.67E-06 | -5.36 | 8.49E-10 |
| ENSGALG00000029404 | ENSGALG00000029404 | 4 | 56619178 | 56620663 | - | -4.62 | 4.60E-40 | -3.56 | 5.56E-14 |
| ENSGALG00000040445 | ENSGALG00000040445 | 6 | 30596635 | 30597795 | + | -4.87 | 1.57E-24 | -5.24 | 1.25E-12 |
| ENSGALG00000032177 | ENSGALG00000032177 | 1 | 93320999 | 93323566 | - | -5.14 | 3.51E-14 | -5.45 | 6.78E-07 |
| XLOC_022036 | XLOC_022036 | 9 | 23434143 | 23437324 | - | -1.16 | 1.25E-04 | -1.25 | 6.28E-04 |
| XLOC_019519 | XLOC_019519 | 6 | 15087318 | 15099944 | - | -1.38 | 2.03E-04 | -1.80 | 1.09E-05 |
| XLOC_023025 | XLOC_023025 | Z | 43898825 | 43915775 | - | -1.90 | 2.95E-04 | -3.64 | 2.54E-11 |
| XLOC_005000 | XLOC_005000 | 12 | 283758 | 431426 | + | -1.95 | 2.35E-06 | -2.82 | 2.41E-10 |
| XLOC_022595 | XLOC_022595 | Z | 24277612 | 24281997 | + | -2.04 | 4.87E-06 | -2.79 | 1.30E-07 |
| XLOC_005557 | XLOC_005557 | 13 | 8607803 | 8611563 | + | -2.23 | 3.52E-06 | -3.83 | 4.85E-09 |
| XLOC_000835 | XLOC_000835 | 1 | 139087443 | 139098712 | + | -2.29 | 1.55E-06 | -3.12 | 1.47E-09 |
| XLOC_018520 | XLOC_018520 | 5 | 42944695 | 43001197 | - | -2.46 | 1.73E-05 | -1.96 | 3.00E-04 |
| XLOC_000513 | XLOC_000513 | 1 | 77596253 | 77602396 | + | -2.52 | 3.25E-08 | -2.88 | 5.83E-10 |
| XLOC_000734 | XLOC_000734 | 1 | 118515513 | 118768849 | + | -2.73 | 5.30E-04 | -7.50 | 5.66E-18 |
| XLOC_000448 | XLOC_000448 | 1 | 71294829 | 71304367 | + | -2.86 | 1.69E-22 | -1.58 | 6.32E-04 |
| XLOC_016303 | XLOC_016303 | 4 | 60457351 | 60473098 | + | -3.49 | 2.18E-07 | -3.99 | 2.70E-04 |
| XLOC_016809 | XLOC_016809 | 4 | 60449281 | 60455137 | - | -4.08 | 5.81E-15 | -2.26 | 5.31E-04 |
| XLOC_001734 | XLOC_001734 | 1 | 120301890 | 120343649 | - | -4.41 | 4.69E-12 | -8.44 | 2.88E-23 |
| XLOC_007641 | XLOC_007641 | 18 | 8149102 | 8214301 | - | -5.12 | 3.73E-35 | -2.49 | 9.10E-04 |
| XLOC_016148 | XLOC_016148 | 4 | 32685636 | 32741041 | + | -5.14 | 1.17E-16 | -4.63 | 4.91E-05 |
| XLOC_000833 | XLOC_000833 | 1 | 138657531 | 138671002 | + | -5.56 | 7.43E-17 | -5.79 | 7.09E-07 |

**S7 Table. Information of ALV invasion-related miRNAs used for constructing lncRNA-miRNA-mRNA regulatory network**

| *miRNA.name* | *Change following ALV infection* | *MiRBase.ID* | *Seed.m8* |
| --- | --- | --- | --- |
| gga-let-7b | up | gga-let-7b | GAGGUAG |
| gga-let-7c | up | gga-let-7c-5p | GAGGUAG |
| gga-let-7c | up | gga-let-7c-3p | UGUACAA |
| gga-let-7d | up | gga-let-7d | GAGGUAG |
| gga-let-7f | up | gga-let-7f-5p | GAGGUAG |
| gga-let-7f | up | gga-let-7f-3p | UAUACAA |
| gga-let-7g | up | gga-let-7g-5p | GAGGUAG |
| gga-let-7g | up | gga-let-7g-3p | UGUACAG |
| gga-let-7i | up | gga-let-7i | GAGGUAG |
| gga-let-7j | up | gga-let-7j-5p | GAGGUAG |
| gga-let-7j | up | gga-let-7j-3p | UAUACAG |
| gga-let-7k | up | gga-let-7k-5p | GAGGUAG |
| gga-let-7k | up | gga-let-7k-3p | UAUACAA |
| gga-miR-103 | up | gga-miR-103-3p | GCAGCAU |
| gga-miR-103 | up | gga-miR-103-1-5p | CGGCUUC |
| gga-miR-103 | up | gga-miR-103-2-5p | GCUUCUU |
| gga-miR-106 | up | gga-miR-106-3p | CUGCAGU |
| gga-miR-106 | up | gga-miR-106-5p | AAAGUGC |
| gga-miR-10a-3p | up | gga-miR-10a-3p | AAUUCGU |
| gga-miR-10a-5p | up | gga-miR-10a-5p | ACCCUGU |
| gga-miR-10b | up | gga-miR-10b-5p | ACCCUGU |
| gga-miR-10b | up | gga-miR-10b-3p | GAUUCGA |
| gga-miR-125b | up | gga-miR-125b-3p | CAAGUCA |
| gga-miR-125b | up | gga-miR-125b-5p | CCCUGAG |
| gga-miR-126-3p | up | gga-miR-126-3p | CGUACCG |
| gga-miR-126-5p | up | gga-miR-126-5p | AUUAUUA |
| gga-miR-128 | up | gga-miR-128-3p | CACAGUG |
| gga-miR-128 | up | gga-miR-128-1-5p | GGGGCCG |
| gga-miR-128 | up | gga-miR-128-2-5p | GGGGCCG |
| gga-miR-1306 | up | gga-miR-1306-3p | GGACGUU |
| gga-miR-1306 | up | gga-miR-1306-5p | CCACCUC |
| gga-miR-137 | up | gga-miR-137-3p | AUUGCUU |
| gga-miR-137 | up | gga-miR-137-5p | CGGGUAU |
| gga-miR-1416-5p | up | gga-miR-1416-5p | CCUUAAC |
| gga-miR-148a | up | gga-miR-148a-3p | CAGUGCA |
| gga-miR-148a | up | gga-miR-148a-5p | AAGUUCU |
| gga-miR-153 | up | gga-miR-153-3p | UGCAUAG |
| gga-miR-153 | up | gga-miR-153-5p | CAUUUUU |
| gga-miR-1552-5p | up | gga-miR-1552-5p | UAGUGCG |
| gga-miR-1559 | up | gga-miR-1559-3p | GUUACAU |
| gga-miR-1559 | up | gga-miR-1559-5p | UCGAUGC |
| gga-miR-15a | up | gga-miR-15a | AGCAGCA |
| gga-miR-1648-5p | up | gga-miR-1648-5p | GGCUCGG |
| gga-miR-1655 | up | gga-miR-1655-3p | UUUACCC |
| gga-miR-1655 | up | gga-miR-1655-5p | CAGCUGG |
| gga-miR-1664-3p | up | gga-miR-1664-3p | CUGUGAC |
| gga-miR-1666 | up | gga-miR-1666 | AACGCCA |
| gga-miR-1684a-3p | up | gga-miR-1684a-3p | AGUAUGA |
| gga-miR-1684b-3p | up | gga-miR-1684b-3p | AGUAUGA |
| gga-miR-17-5p | up | gga-miR-17-5p | AAAGUGC |
| gga-miR-1729-5p | up | gga-miR-1729-5p | UCCCUUA |
| gga-miR-1744-3p | up | gga-miR-1744-3p | CUUCAAC |
| gga-miR-1782 | up | gga-miR-1782 | CAUUCAU |
| gga-miR-181a-5p | up | gga-miR-181a-5p | ACAUUCA |
| gga-miR-181b | up | gga-miR-181b-5p | ACAUUCA |
| gga-miR-181b | up | gga-miR-181b-1-3p | CACUGAA |
| gga-miR-181b | up | gga-miR-181b-2-3p | CACUGAU |
| gga-miR-196 | up | gga-miR-196-1-3p | AAGAACA |
| gga-miR-196 | up | gga-miR-196-2-3p | UACAGCA |
| gga-miR-196 | up | gga-miR-196-5p | AGGUAGU |
| gga-miR-200a | up | gga-miR-200a-3p | AACACUG |
| gga-miR-200a | up | gga-miR-200a-5p | AUCUUAC |
| gga-miR-200b | up | gga-miR-200b-3p | AAUACUG |
| gga-miR-200b | up | gga-miR-200b-5p | CUUACUG |
| gga-miR-204 | up | gga-miR-204 | UCCCUUU |
| gga-miR-206 | up | gga-miR-206 | GGAAUGU |
| gga-miR-20a | up | gga-miR-20a-5p | AAAGUGC |
| gga-miR-20a | up | gga-miR-20a-3p | AUCUACU |
| gga-miR-211 | up | gga-miR-211 | UCCCUUU |
| gga-miR-2130 | up | gga-miR-2130 | CCCAGUG |
| gga-miR-214 | up | gga-miR-214 | CAGCAGG |
| gga-miR-218 | up | gga-miR-218-3p | AUGGUUC |
| gga-miR-218 | up | gga-miR-218-5p | UGUGCUU |
| gga-miR-218 | up | gga-miR-2188-3p | AUAUAUG |
| gga-miR-218 | up | gga-miR-2188-5p | AGGUCCA |
| gga-miR-2188 | up | gga-miR-2188-3p | AUAUAUG |
| gga-miR-2188 | up | gga-miR-2188-5p | AGGUCCA |
| gga-miR-221 | up | gga-miR-221-3p | GCUACAU |
| gga-miR-221 | up | gga-miR-221-5p | ACCUGGC |
| gga-miR-223 | up | gga-miR-223 | GUCAGUU |
| gga-miR-23b | up | gga-miR-23b-3p | UCACAUU |
| gga-miR-23b | up | gga-miR-23b-5p | GGUUCCU |
| gga-miR-24 | up | gga-miR-24-3p | GGCUCAG |
| gga-miR-24 | up | gga-miR-24-5p | UGCCUAC |
| gga-miR-26a | up | gga-miR-26a-3p | CUAUUCU |
| gga-miR-26a | up | gga-miR-26a-5p | UCAAGUA |
| gga-miR-27b | up | gga-miR-27b-3p | UCACAGU |
| gga-miR-27b | up | gga-miR-27b-5p | GAGCUUA |
| gga-miR-29b | up | gga-miR-29b-3p | AGCACCA |
| gga-miR-29b | up | gga-miR-29b-1-5p | GCUGGUU |
| gga-miR-29b | up | gga-miR-29b-2-5p | GCUGGUU |
| gga-miR-30b | up | gga-miR-30b-5p | GUAAACA |
| gga-miR-30b | up | gga-miR-30b-3p | UGGGGGG |
| gga-miR-30c | up | gga-miR-30c-2-3p | GGGAGAA |
| gga-miR-30c | up | gga-miR-30c-5p | GUAAACA |
| gga-miR-30c | up | gga-miR-30c-1-3p | GGGAGAG |
| gga-miR-30d | up | gga-miR-30d | GUAAACA |
| gga-miR-32 | up | gga-miR-32-3p | AUUUAGU |
| gga-miR-32 | up | gga-miR-32-5p | AUUGCAC |
| gga-miR-33 | up | gga-miR-33-3p | AUGUUCC |
| gga-miR-33 | up | gga-miR-33-5p | UGCAUUG |
| gga-miR-34b | up | gga-miR-34b-5p | AGGCAGU |
| gga-miR-34b | up | gga-miR-34b-3p | AUCACUA |
| gga-miR-34c | up | gga-miR-34c-5p | GGCAGUG |
| gga-miR-34c | up | gga-miR-34c-3p | AUCACUA |
| gga-miR-3525 | up | gga-miR-3525 | AGCCAUU |
| gga-miR-3531 | up | gga-miR-3531-3p | CUUGCAA |
| gga-miR-3531 | up | gga-miR-3531-5p | CCUUGUU |
| gga-miR-3532 | up | gga-miR-3532-3p | UGGAGGC |
| gga-miR-3532 | up | gga-miR-3532-5p | UUGCACU |
| gga-miR-365 | up | gga-miR-365-1-5p | AGGGACU |
| gga-miR-365 | up | gga-miR-365-2-5p | AGGGACU |
| gga-miR-365 | up | gga-miR-365-3p | AAUGCCC |
| gga-miR-365 | up | gga-miR-365b-5p | GGAUUUU |
| gga-miR-383 | up | gga-miR-383-3p | CACAGCA |
| gga-miR-383 | up | gga-miR-383-5p | GAUCAGA |
| gga-miR-429 | up | gga-miR-429-3p | AAUACUG |
| gga-miR-429 | up | gga-miR-429-5p | GUCUUAC |
| gga-miR-449a | up | gga-miR-449a | GGCAGUG |
| gga-miR-449b-5p | up | gga-miR-449b-5p | GGCAGUG |
| gga-miR-449c-5p | up | gga-miR-449c-5p | GGCAGUG |
| gga-miR-460b-5p | up | gga-miR-460b-5p | CCUCAUU |
| gga-miR-499 | up | gga-miR-499-3p | ACAUCAC |
| gga-miR-499 | up | gga-miR-499-5p | UAAGACU |
| gga-miR-551-3p | up | gga-miR-551-3p | CGACCCA |
| gga-miR-6552-3p | up | gga-miR-6552-3p | AACAAGA |
| gga-miR-6552-5p | up | gga-miR-6552-5p | UCUGUCC |
| gga-miR-6557-3p | up | gga-miR-6557-3p | GCGCCGA |
| gga-miR-6557-5p | up | gga-miR-6557-5p | CGGAGGA |
| gga-miR-6568-3p | up | gga-miR-6568-3p | CAACCAA |
| gga-miR-6606-5p | up | gga-miR-6606-5p | AGGAGCG |
| gga-miR-6677-5p | up | gga-miR-6677-5p | UCACUGC |
| gga-miR-92 | up | gga-miR-92-3p | AUUGCAC |
| gga-miR-92 | up | gga-miR-92-5p | GGUUGGG |
| gga-miR-100 | down | gga-miR-100-3p | AGCUUGU |
| gga-miR-100 | down | gga-miR-100-5p | ACCCGUA |
| gga-miR-130a | down | gga-miR-130a-3p | AGUGCAA |
| gga-miR-130a | down | gga-miR-130a-5p | CCCUUUU |
| gga-miR-130c-3p | down | gga-miR-130c-3p | AGUGCAA |
| gga-miR-130c-5p | down | gga-miR-130c-5p | CCCUUUU |
| gga-miR-1329-5p | down | gga-miR-1329-5p | ACAGUGA |
| gga-miR-133a | down | gga-miR-133a-3p | UGGUCCC |
| gga-miR-133a | down | gga-miR-133a-5p | GCUGGUA |
| gga-miR-133b | down | gga-miR-133b | UGGUCCC |
| gga-miR-133c | down | gga-miR-133c-3p | UGGUCCC |
| gga-miR-133c | down | gga-miR-133c-5p | CUGGUAA |
| gga-miR-138 | down | gga-miR-138-1-3p | ACUUCAC |
| gga-miR-138 | down | gga-miR-138-2-3p | AUUUCAC |
| gga-miR-138 | down | gga-miR-138-5p | GCUGGUG |
| gga-miR-140-3p | down | gga-miR-140-3p | CACAGGG |
| gga-miR-142-3p | down | gga-miR-142-3p | GUAGUGU |
| gga-miR-142-5p | down | gga-miR-142-5p | CCAUAAA |
| gga-miR-144 | down | gga-miR-144-3p | UACAGUA |
| gga-miR-144 | down | gga-miR-144-5p | GAUAUCA |
| gga-miR-1452 | down | gga-miR-1452 | UGAGAUA |
| gga-miR-1458 | down | gga-miR-1458 | UCCUGUG |
| gga-miR-146a | down | gga-miR-146a-5p | GAGAACU |
| gga-miR-146a | down | gga-miR-146a-3p | CCCAUGG |
| gga-miR-146b-3p | down | gga-miR-146b-3p | CCUAUGG |
| gga-miR-146c-3p | down | gga-miR-146c-3p | GUCCAUG |
| gga-miR-146c-5p | down | gga-miR-146c-5p | GAGAACU |
| gga-miR-147 | down | gga-miR-147 | UGUGCGG |
| gga-miR-155 | down | gga-miR-155 | UAAUGCU |
| gga-miR-1563 | down | gga-miR-1563 | CACAUGA |
| gga-miR-1585 | down | gga-miR-1585 | GUGCCCA |
| gga-miR-1587 | down | gga-miR-1587 | GGCUGGG |
| gga-miR-15b | down | gga-miR-15b-5p | AGCAGCA |
| gga-miR-15b | down | gga-miR-15b-3p | AAUCAUU |
| gga-miR-15c-5p | down | gga-miR-15c-5p | AGCAGCA |
| gga-miR-16 | down | gga-miR-1696 | UGCUGUG |
| gga-miR-16 | down | gga-miR-16-5p | AGCAGCA |
| gga-miR-16 | down | gga-miR-16c-5p | AGCAGCA |
| gga-miR-16 | down | gga-miR-1600 | GGGCUGA |
| gga-miR-16 | down | gga-miR-1601 | GUGUGAG |
| gga-miR-16 | down | gga-miR-1673 | GUGUGAG |
| gga-miR-16 | down | gga-miR-1602 | GGGCUCU |
| gga-miR-16 | down | gga-miR-1603 | GUGGUUG |
| gga-miR-16 | down | gga-miR-1604 | GGGCCCA |
| gga-miR-16 | down | gga-miR-1605 | GGGUUUC |
| gga-miR-16 | down | gga-miR-1606 | GGGGGCA |
| gga-miR-16 | down | gga-miR-1607 | GGGGCGG |
| gga-miR-16 | down | gga-miR-1608 | GGGACAG |
| gga-miR-16 | down | gga-miR-1609 | GGCUGAG |
| gga-miR-16 | down | gga-miR-1610 | GGCUUGU |
| gga-miR-16 | down | gga-miR-1611 | GAGGGCU |
| gga-miR-16 | down | gga-miR-1612 | AAGGAAA |
| gga-miR-16 | down | gga-miR-1613 | ACAUGCG |
| gga-miR-16 | down | gga-miR-16-1-3p | CAGUAUU |
| gga-miR-16 | down | gga-miR-16c-3p | CAGUAUU |
| gga-miR-16 | down | gga-miR-1614-3p | AGGGAGG |
| gga-miR-16 | down | gga-miR-1614-5p | GCAUGGC |
| gga-miR-16 | down | gga-miR-1615 | GGCAGCU |
| gga-miR-16 | down | gga-miR-1616 | GGAUCCU |
| gga-miR-16 | down | gga-miR-1617 | GAGGCCU |
| gga-miR-16 | down | gga-miR-1618-3p | AGCCCGG |
| gga-miR-16 | down | gga-miR-1618-5p | GCAUCCU |
| gga-miR-16 | down | gga-miR-1619 | GAAUUCA |
| gga-miR-16 | down | gga-miR-1620 | CUUUAAU |
| gga-miR-16 | down | gga-miR-1621-3p | GUUCGCC |
| gga-miR-16 | down | gga-miR-1621-5p | CCGGCUG |
| gga-miR-16 | down | gga-miR-1622 | ACAUAUG |
| gga-miR-16 | down | gga-miR-1623 | CAGGCAC |
| gga-miR-16 | down | gga-miR-16-2-3p | CAAUAUU |
| gga-miR-16 | down | gga-miR-1624 | CACCGCA |
| gga-miR-16 | down | gga-miR-1625-3p | CAGCAGA |
| gga-miR-16 | down | gga-miR-1625-5p | GGACCAG |
| gga-miR-16 | down | gga-miR-1626-3p | CUGGAAG |
| gga-miR-16 | down | gga-miR-1626-5p | UUCCAUG |
| gga-miR-16 | down | gga-miR-1627-3p | AGCCACG |
| gga-miR-16 | down | gga-miR-1627-5p | AAGUGAC |
| gga-miR-16 | down | gga-miR-1628 | AGAGCUC |
| gga-miR-16 | down | gga-miR-1629 | GCUGUCG |
| gga-miR-16 | down | gga-miR-1630 | AAGAGGA |
| gga-miR-16 | down | gga-miR-1631 | AACUGGC |
| gga-miR-16 | down | gga-miR-1632-3p | ACUCAUU |
| gga-miR-16 | down | gga-miR-1632-5p | GCUUGUU |
| gga-miR-16 | down | gga-miR-1633 | GCGCUUC |
| gga-miR-16 | down | gga-miR-1634 | GCGACGC |
| gga-miR-16 | down | gga-miR-1635 | GCCCAGG |
| gga-miR-16 | down | gga-miR-1636 | GCAGGUG |
| gga-miR-16 | down | gga-miR-1637 | GAUAGCU |
| gga-miR-16 | down | gga-miR-1638 | UAGUUUG |
| gga-miR-16 | down | gga-miR-1639 | UGUGCAA |
| gga-miR-16 | down | gga-miR-1640 | UGUCACU |
| gga-miR-16 | down | gga-miR-1641 | GAGGAUU |
| gga-miR-16 | down | gga-miR-1642 | GAGAGGC |
| gga-miR-16 | down | gga-miR-1643-3p | AAAAGUG |
| gga-miR-16 | down | gga-miR-1644 | CUGUUGU |
| gga-miR-16 | down | gga-miR-1645 | CUGCUGA |
| gga-miR-16 | down | gga-miR-1647 | CUCUGCC |
| gga-miR-16 | down | gga-miR-1648-3p | GGGAGUG |
| gga-miR-16 | down | gga-miR-1648-5p | GGCUCGG |
| gga-miR-16 | down | gga-miR-1649-3p | UCCGUGC |
| gga-miR-16 | down | gga-miR-1649-5p | CCUGCAG |
| gga-miR-16 | down | gga-miR-1650 | CCUCUGA |
| gga-miR-16 | down | gga-miR-1651-3p | UGCUUUU |
| gga-miR-16 | down | gga-miR-1651-5p | CUAAGAC |
| gga-miR-16 | down | gga-miR-1652 | CCCUGUG |
| gga-miR-16 | down | gga-miR-1653 | AGGAGCU |
| gga-miR-16 | down | gga-miR-1654 | UGCUGGU |
| gga-miR-16 | down | gga-miR-1655-3p | UUUACCC |
| gga-miR-16 | down | gga-miR-1655-5p | CAGCUGG |
| gga-miR-16 | down | gga-miR-1656 | CACCAGC |
| gga-miR-16 | down | gga-miR-1657 | AUAUGUG |
| gga-miR-16 | down | gga-miR-1658-3p | AGCUGUG |
| gga-miR-16 | down | gga-miR-1658-5p | AUACCAC |
| gga-miR-16 | down | gga-miR-1659 | AGGGCAG |
| gga-miR-16 | down | gga-miR-1660 | AGGCCUG |
| gga-miR-16 | down | gga-miR-1661 | AGCAGAU |
| gga-miR-16 | down | gga-miR-1662 | UGACAUC |
| gga-miR-16 | down | gga-miR-1663-3p | GGCAUCC |
| gga-miR-16 | down | gga-miR-1663-5p | ACCACUG |
| gga-miR-16 | down | gga-miR-1664-3p | CUGUGAC |
| gga-miR-16 | down | gga-miR-1664-5p | CAGACAG |
| gga-miR-16 | down | gga-miR-1665 | AAGGCCC |
| gga-miR-16 | down | gga-miR-1666 | AACGCCA |
| gga-miR-16 | down | gga-miR-1667-3p | AACAUGA |
| gga-miR-16 | down | gga-miR-1667-5p | AGAUUCU |
| gga-miR-16 | down | gga-miR-1668-3p | GCUGUGU |
| gga-miR-16 | down | gga-miR-1668-5p | UGGGCUG |
| gga-miR-16 | down | gga-miR-1669 | UGGGCUC |
| gga-miR-16 | down | gga-miR-1670 | UGGGACG |
| gga-miR-16 | down | gga-miR-1671 | UGAGGAC |
| gga-miR-16 | down | gga-miR-1672 | UCAGGCC |
| gga-miR-16 | down | gga-miR-1674 | GGCUAUG |
| gga-miR-16 | down | gga-miR-1675 | GACGCCA |
| gga-miR-16 | down | gga-miR-1676-3p | GAGCGAA |
| gga-miR-16 | down | gga-miR-1676-5p | CUGACUC |
| gga-miR-16 | down | gga-miR-1677-3p | GACUUCA |
| gga-miR-16 | down | gga-miR-1677-5p | CCUGCAC |
| gga-miR-16 | down | gga-miR-1678 | UUGACAA |
| gga-miR-16 | down | gga-miR-1679 | UGUCAGC |
| gga-miR-16 | down | gga-miR-1680-3p | AGGCGGU |
| gga-miR-16 | down | gga-miR-1680-5p | UCCUCCU |
| gga-miR-16 | down | gga-miR-1681 | UCACAUG |
| gga-miR-16 | down | gga-miR-1682 | GGGUCAG |
| gga-miR-16 | down | gga-miR-1683 | CUGGGAC |
| gga-miR-16 | down | gga-miR-1684a-3p | AGUAUGA |
| gga-miR-16 | down | gga-miR-1684b-3p | AGUAUGA |
| gga-miR-16 | down | gga-miR-1684a-5p | GCUCUGC |
| gga-miR-16 | down | gga-miR-1685-3p | CAGUAAU |
| gga-miR-16 | down | gga-miR-1685-5p | GGAGUCA |
| gga-miR-16 | down | gga-miR-1686 | GGAGGCU |
| gga-miR-16 | down | gga-miR-1687-3p | UGCUGGC |
| gga-miR-16 | down | gga-miR-1687-5p | GAGGAAA |
| gga-miR-16 | down | gga-miR-1688 | GAGCAUG |
| gga-miR-16 | down | gga-miR-1689-3p | CGGUGAC |
| gga-miR-16 | down | gga-miR-1689-5p | CUCUGCU |
| gga-miR-16 | down | gga-miR-1690-5p | CCAGAGG |
| gga-miR-16 | down | gga-miR-1691 | CAGCUGA |
| gga-miR-16 | down | gga-miR-1692 | GUAGCUC |
| gga-miR-16 | down | gga-miR-1693 | CAAAGGA |
| gga-miR-16 | down | gga-miR-1694 | GAGGACG |
| gga-miR-16 | down | gga-miR-1695 | AGCACAG |
| gga-miR-16 | down | gga-miR-1697 | UCUCAUG |
| gga-miR-16 | down | gga-miR-1698 | GAGGCUG |
| gga-miR-16 | down | gga-miR-1699 | CAGAGGG |
| gga-miR-16 | down | gga-miR-1646 | CUGCCCU |
| gga-miR-16 | down | gga-miR-1643-5p | CUUAUCA |
| gga-miR-1626-5p | down | gga-miR-1626-5p | UUCCAUG |
| gga-miR-1662 | down | gga-miR-1662 | UGACAUC |
| gga-miR-1668-3p | down | gga-miR-1668-3p | GCUGUGU |
| gga-miR-1668-5p | down | gga-miR-1668-5p | UGGGCUG |
| gga-miR-1674 | down | gga-miR-1674 | GGCUAUG |
| gga-miR-1698 | down | gga-miR-1698 | GAGGCUG |
| gga-miR-1699 | down | gga-miR-1699 | CAGAGGG |
| gga-miR-16c | down | gga-miR-16c-5p | AGCAGCA |
| gga-miR-16c | down | gga-miR-16c-3p | CAGUAUU |
| gga-miR-1712-3p | down | gga-miR-1712-3p | UCAGUUA |
| gga-miR-1728-3p | down | gga-miR-1728-3p | GGCUUCU |
| gga-miR-1742 | down | gga-miR-1742 | AUCGUCG |
| gga-miR-1756a | down | gga-miR-1756a | CCAGUGA |
| gga-miR-1769-3p | down | gga-miR-1769-3p | GUGUGAA |
| gga-miR-1769-5p | down | gga-miR-1769-5p | UUCAGGC |
| gga-miR-1797 | down | gga-miR-1797 | CUUGGAA |
| gga-miR-181a-3p | down | gga-miR-181a-3p | CCAUCGA |
| gga-miR-183 | down | gga-miR-183 | AUGGCAC |
| gga-miR-18a | down | gga-miR-18a-3p | CUGCCCU |
| gga-miR-18a | down | gga-miR-18a-5p | AAGGUGC |
| gga-miR-18b | down | gga-miR-18b-3p | ACUGCCC |
| gga-miR-18b | down | gga-miR-18b-5p | AAGGUGC |
| gga-miR-190 | down | gga-miR-190a-3p | UAUAUAU |
| gga-miR-190 | down | gga-miR-190a-5p | GAUAUGU |
| gga-miR-193a | down | gga-miR-193a-3p | ACUGGCC |
| gga-miR-193a | down | gga-miR-193a-5p | GGGUCUU |
| gga-miR-193b | down | gga-miR-193b-3p | ACUGGCC |
| gga-miR-193b | down | gga-miR-193b-5p | GGGGUUU |
| gga-miR-194 | down | gga-miR-194 | GUAACAG |
| gga-miR-199-3p | down | gga-miR-199-3p | ACAGUAG |
| gga-miR-199-5p | down | gga-miR-199-5p | CCAGUGU |
| gga-miR-19a | down | gga-miR-19a-3p | GUGCAAA |
| gga-miR-19a | down | gga-miR-19a-5p | GUUUUGC |
| gga-miR-19b | down | gga-miR-19b-3p | GUGCAAA |
| gga-miR-19b | down | gga-miR-19b-5p | GUUUUGC |
| gga-miR-20b | down | gga-miR-20b-5p | AAAGUGC |
| gga-miR-20b | down | gga-miR-20b-3p | CUGUAAU |
| gga-miR-21 | down | gga-miR-211 | UCCCUUU |
| gga-miR-21 | down | gga-miR-2126 | GCGGCGC |
| gga-miR-21 | down | gga-miR-2127 | GGGAGGG |
| gga-miR-21 | down | gga-miR-2128 | GCAGUGA |
| gga-miR-21 | down | gga-miR-2129 | AGCAGGA |
| gga-miR-21 | down | gga-miR-2130 | CCCAGUG |
| gga-miR-21 | down | gga-miR-2131-3p | UGUUACU |
| gga-miR-21 | down | gga-miR-2131-5p | UGCAGAA |
| gga-miR-21 | down | gga-miR-21-3p | AACAACA |
| gga-miR-21 | down | gga-miR-214 | CAGCAGG |
| gga-miR-21 | down | gga-miR-215-3p | CUGUCAU |
| gga-miR-21 | down | gga-miR-215-5p | UGACCUA |
| gga-miR-21 | down | gga-miR-21-5p | AGCUUAU |
| gga-miR-21 | down | gga-miR-216a | AAUCUCA |
| gga-miR-21 | down | gga-miR-216b | AAUCUCU |
| gga-miR-21 | down | gga-miR-216c | UCUCUAC |
| gga-miR-21 | down | gga-miR-217-3p | CAUCAGU |
| gga-miR-21 | down | gga-miR-217-5p | ACUGCAU |
| gga-miR-21 | down | gga-miR-218-3p | AUGGUUC |
| gga-miR-21 | down | gga-miR-218-5p | UGUGCUU |
| gga-miR-21 | down | gga-miR-2188-3p | AUAUAUG |
| gga-miR-21 | down | gga-miR-2188-5p | AGGUCCA |
| gga-miR-21 | down | gga-miR-219a | GAUUGUC |
| gga-miR-21 | down | gga-miR-219b | ACAAGAA |
| gga-miR-215 | down | gga-miR-215-3p | CUGUCAU |
| gga-miR-215 | down | gga-miR-215-5p | UGACCUA |
| gga-miR-22-3p | down | gga-miR-22-3p | AGCUGCC |
| gga-miR-222a | down | gga-miR-222a | GCUACAU |
| gga-miR-222b-3p | down | gga-miR-222b-3p | GCUACAU |
| gga-miR-222b-5p | down | gga-miR-222b-5p | GCUCAGU |
| gga-miR-301a | down | gga-miR-301a-3p | AGUGCAA |
| gga-miR-301a | down | gga-miR-301a-5p | CUGACAA |
| gga-miR-301b-3p | down | gga-miR-301b-3p | AGUGCAA |
| gga-miR-30a-5p | down | gga-miR-30a-5p | GUAAACA |
| gga-miR-30e | down | gga-miR-30e-5p | GUAAACA |
| gga-miR-30e | down | gga-miR-30e-3p | UUCAGUC |
| gga-miR-34a | down | gga-miR-34a-5p | GGCAGUG |
| gga-miR-34a | down | gga-miR-34a-3p | AAUCAGC |
| gga-miR-3523 | down | gga-miR-3523 | CGCGCAG |
| gga-miR-3535 | down | gga-miR-3535 | GAUAUGA |
| gga-miR-3536 | down | gga-miR-3536 | UGCAUAC |
| gga-miR-451 | down | gga-miR-451 | AACCGUU |
| gga-miR-454-3p | down | gga-miR-454-3p | AGUGCAA |
| gga-miR-456 | down | gga-miR-456-3p | AGGCUGG |
| gga-miR-456 | down | gga-miR-456-5p | AGGCAUC |
| gga-miR-458a-3p | down | gga-miR-458a-3p | UAGCUCU |
| gga-miR-490 | down | gga-miR-490-3p | AACCUGG |
| gga-miR-490 | down | gga-miR-490-5p | CAUGGAU |
| gga-miR-6542-3p | down | gga-miR-6542-3p | CGGGACA |
| gga-miR-6546-5p | down | gga-miR-6546-5p | GGAGCGC |
| gga-miR-6548-5p | down | gga-miR-6548-5p | ACAACAG |
| gga-miR-6553-3p | down | gga-miR-6553-3p | ACACUAC |
| gga-miR-6614-3p | down | gga-miR-6614-3p | CAGGACA |
| gga-miR-6631-5p | down | gga-miR-6631-5p | AAGAGAA |
| gga-miR-6649-5p | down | gga-miR-6649-5p | UUAUCAC |
| gga-miR-9-3p | down | gga-miR-9-3p | AAAGCUA |
| gga-miR-99a-3p | down | gga-miR-99a-3p | AAGCUCG |
| gga-miR-99a-5p | down | gga-miR-99a-5p | ACCCGUA |

Notes: These miRNAs were identified being differentially expressed in spleen post ALV-J subgroup virus infection in a previous study. The information of name and expression change direction following ALV infection for these miRNAs were obtained from the original paper. Seed sequence information were obtained from TargetScan database and both -5p and -3p of the indicated miRNAs were included.

**S8 Table. Primers used in ddPCR validation**

| **Gene name** | **Primer name** | **Sequence (5’ – 3’)** | **PCR product size (bp)** |
| --- | --- | --- | --- |
| *ENSGALG00000043746* | linc-1-F | CAACTTCACTCCTTAGGCAC | 157 |
|  | linc-1-R | CACATGCTCGTTCTATCCTC |  |
| *ENSGALG00000040445* | linc-2-F | GGCCTGATCTACTGATACTGG | 109 |
|  | linc-2-R | GCCAACTCTGTCTACTCATAGC |  |
| *ENSGALG00000029404* | linc-3-F | CCTGGAAGAGGAAAGAAGGA | 149 |
|  | linc-3-R | GCTATAGGATAGAGGCTCCTG |  |
| *ENSGALG00000042116* | linc-4-F | GAGAAGACGTCTCTTCCATAGG | 89 |
|  | linc-4-R | TATTCCCAGACTGAACCCTC |  |
| *ENSGALG00000042098* | linc-5-F | GCCAGAAGGTTCAGTACATC | 80 |
|  | linc-5-R | CGTATTACACACTGCTCCAG |  |
| *XLOC_005557* | linc-6-F | CCTATATCTCAGGGATGAGCTG | 173 |
|  | linc-6-R | CTTTTCTCGCTCTCTGATCC |  |
| *XLOC_022595* | linc-7-F | CCCAGCTAGATGCAAACAG | 183 |
|  | linc-7-R | GAGGAGACCTATCCTACCTAAG |  |
| *XLOC_001407* | linc-8-F | GACTCTATCCCTTCCTAACCTC | 92 |
|  | linc-8-R | CTTGCCAACTACTCCAAGAG |  |
| *SOCS2* | PSG-1-F | CATTCCGAGTATCTGCTGAC | 161 |
|  | PSG-1-R | ACGTAGTACTCGATCAGGTG |  |
| *PLPP4* | PSG-2-F | CTCTAGCTCTGAATGGAGTCTG | 176 |
|  | PSG-2-R | GGAAGAGTGGATACTCGGAAAG |  |
| *UGT8* | PSG-3-F | GAGTATCTTCTCTGGGAGACTG | 152 |
|  | PSG-3-R | TCTCATTGGGATCTACCAGG |  |
| *FUT9* | PSG-4-F | CTAATCCTGTGGAATCAGCC | 181 |
|  | PSG-4-R | GAGCGGTCAATAGTCAGATG |  |
| *BMPER* | PSG-5-F | GTCATTAAGCAGAGAGGAGC | 141 |
|  | PSG-5-R | CTCTATTATCACTCCCTCCTGG |  |
| *SH3TC2* | PSG-6-F | GGCAGGAAGGCTGTATTATC | 79 |
|  | PSG-6-R | CAGAGCAGTCTCAATAGCTG |  |
| *ENC1* | PSG-7-F | CAGTGTGAGAACAGATGGAC | 115 |
|  | PSG-7-R | CAGAGAATTCCGTATCTCCACC |  |
| *BICD1* | PSG-8-F | GCAGAGGATACGAATGAAGG | 83 |
|  | PSG-8-R | CTTCAAGTTCGGTGTAGTCC |  |


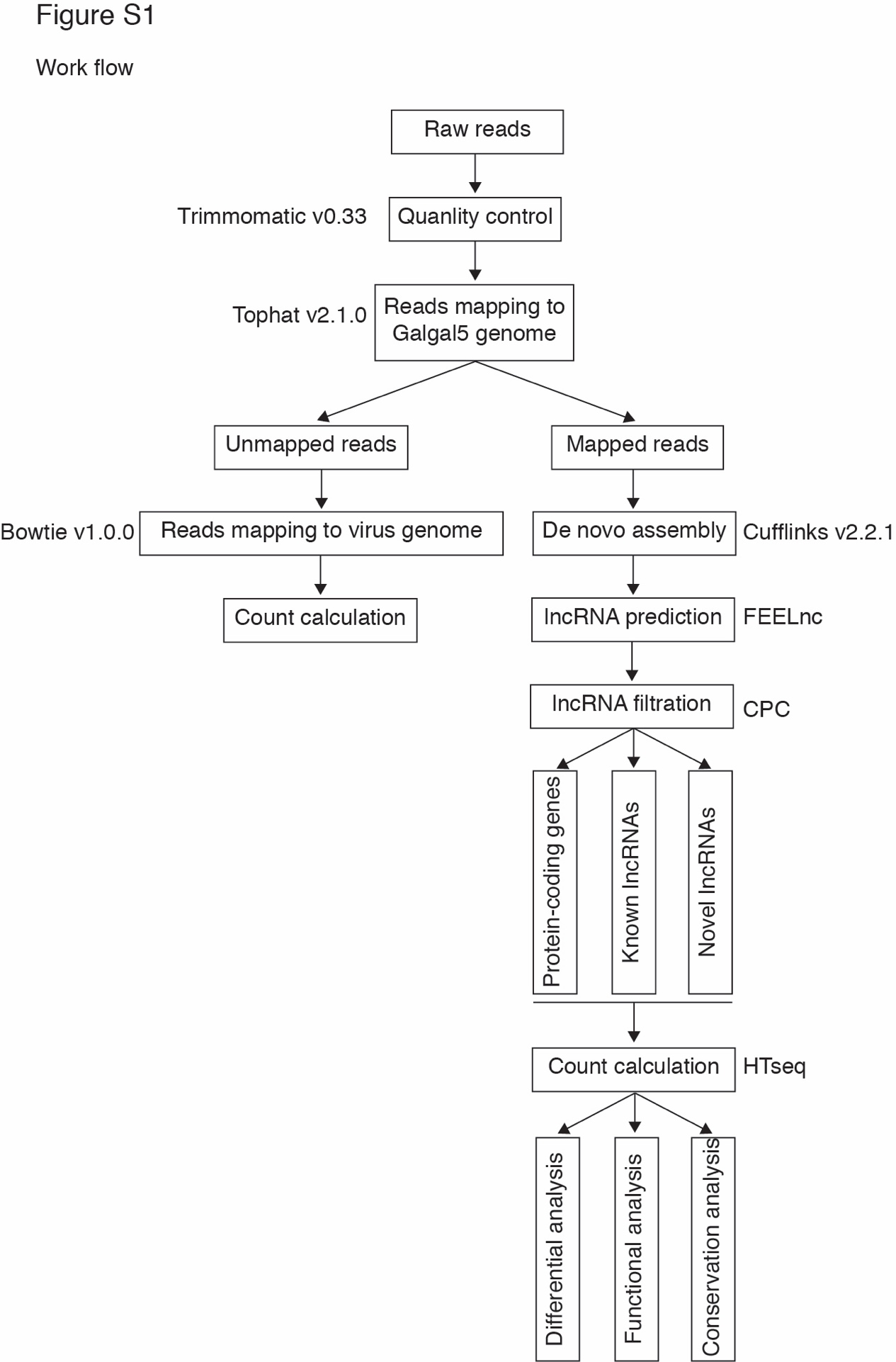


**S1 Fig. A chart of workflow of the RNA-seq analysis**

**
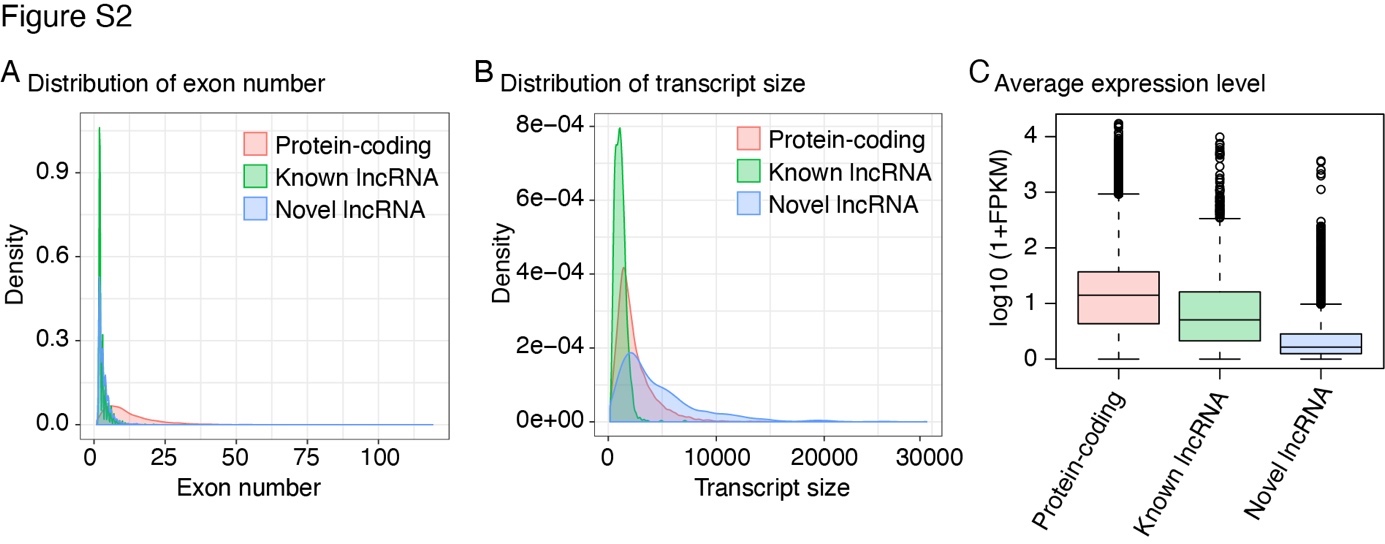
**

**S2 Fig. General features of novel lncRNAs as compared to protein-coding genes and known lncRNAs. (A)** The distribution of exon numbers; **(B)** The transcript size; and **(C)** The average expression level of novel lncRNAs as compared to protein-coding genes and known lncRNAs.


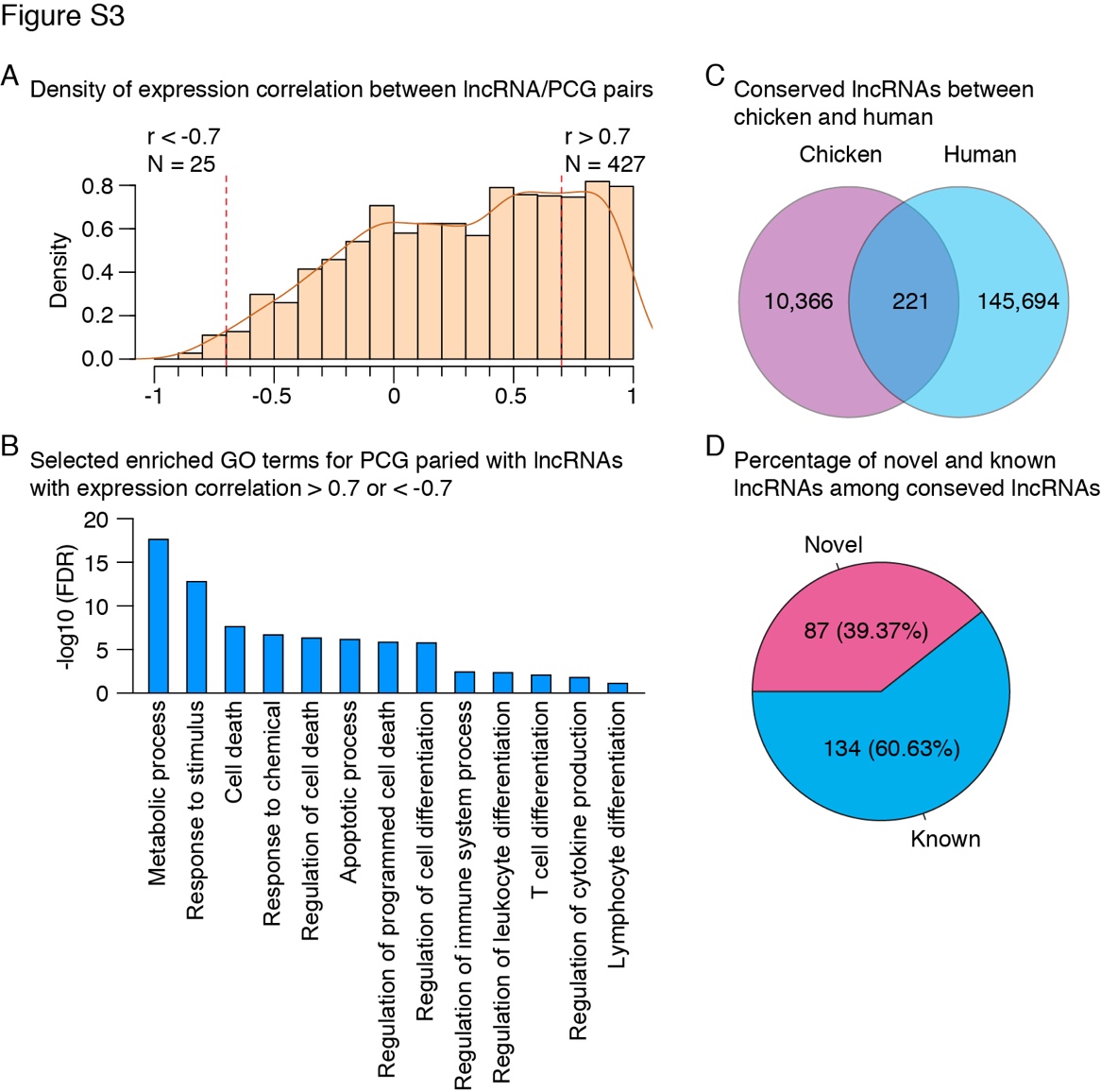


**S3 Fig. Analysis of protein-coding gene partners and conservation of lncRNAs in human. (A)** Density of correlation (r) between the expression of lncRNAs and their protein-coding gene partners; **(B)** Selected significantly enriched gene ontology (GO) biological processes for protein-coding genes paired lncRNAs with their expression correlation > 0.7 or < -0.7; **(C)** The number of conserved lncRNAs between chicken and human; **(D)** The number and percentage of novel and known lncRNAs among chicken lncRNAs that are conserved in human.
